# Discovery of Chemosensory Genes in the Oriental Fruit Fly, *Bactrocera dorsalis*

**DOI:** 10.1371/journal.pone.0129794

**Published:** 2015-06-12

**Authors:** Zhongzhen Wu, He Zhang, Zhengbing Wang, Shuying Bin, Hualiang He, Jintian Lin

**Affiliations:** Institute for Management of Invasive Alien Species, Zhongkai University of Agriculture and Engineering, Guangzhou, Guangdong, People’s Republic of China; United States Department of Agriculture, Beltsville Agricultural Research Center, UNITED STATES

## Abstract

The oriental fruit fly, *Bactrocera dorsalis*, is a devastating fruit fly pest in tropical and sub-tropical countries. Like other insects, this fly uses its chemosensory system to efficiently interact with its environment. However, our understanding of the molecular components comprising *B*. *dorsalis* chemosensory system is limited. Using next generation sequencing technologies, we sequenced the transcriptome of four *B*. *dorsalis* developmental stages: egg, larva, pupa and adult chemosensory tissues. A total of 31 candidate odorant binding proteins (OBPs), 4 candidate chemosensory proteins (CSPs), 23 candidate odorant receptors (ORs), 11 candidate ionotropic receptors (IRs), 6 candidate gustatory receptors (GRs) and 3 candidate sensory neuron membrane proteins (SNMPs) were identified. The tissue distributions of the OBP and CSP transcripts were determined by RT-PCR and a subset of nine genes were further characterized. The predicted proteins from these genes shared high sequence similarity to *Drosophila melanogaster* pheromone binding protein related proteins (PBPRPs). Interestingly, one OBP (BdorOBP19c) was exclusively expressed in the sex pheromone glands of mature females. RT-PCR was also used to compare the expression of the candidate genes in the antennae of male and female *B*. *dorsalis* adults. These antennae-enriched OBPs, CSPs, ORs, IRs and SNMPs could play a role in the detection of pheromones and general odorants and thus could be useful target genes for the integrated pest management of *B*. *dorsalis* and other agricultural pests.

## Introduction

Chemoreception plays a crucial role in insects such as agricultural pests, disease vectors and social insects. These insects use two sensations, olfaction and gustation, to evaluate and locate food sources, shelter, mates, and oviposition sites as well as to avoid predators and other dangers [1–5]. The major molecular components of insect olfaction include odorant-binding proteins (OBPs), odorant receptors (ORs), ionotropic receptors (IRs), sensory neuron membrane proteins (SNMPs) and odorant-degrading enzyme (ODEs) [6], and the major gustatory or contact chemosensation-related proteins are gustatory receptors (GRs) [7,8]. In addition, chemosensory proteins (CSPs) are also found in olfactory and gustatory organs of insects and are involved in the detection of chemicals [9–14].

Chemosensory proteins are widely used by tephritid fruit flies to locate host plants and thereby cause major losses in fruits and vegetables worldwide. Because of their devastating impact on agriculture they are often the target of intense insecticide applications in order to protect commercial production of agricultural crops. The oriental fruit fly, *Bactrocera dorsalis*, is the main fruit fly pest in tropical and sub-tropical countries, and is reported to feed on >117 host species, in 76 genera and 37 families [15]. Since sporadic outbreaks of the pest have been reported worldwide, this fly has been the target of global integrated pest management [16,17,18].

Previous studies have shown that *B*. *dorsalis* exhibits sexually dimorphic behavior, which is influenced by olfactory cues expressed during specific developmental stages [19,20,21]. When the male flies reach sexual maturity, they are strongly attracted to and compulsively feed on Methyl eugenol (ME) (non-host compounds), and this behavior is used to control *B*. *dorsalis* via male annihilation technique through mass trapping [22]. Behavioral assays with electro-physiologically active compounds from mango as a host plant, revealed that γ-octalactone induced oviposition by gravid *B*. *dorsalis* females [20]. In addition, the pest control strategy based on the behavioral manipulation of *B*. *dorsalis* still relies on ME based male lure [23]. However, the molecular mechanisms of behavior-based pest control are not clearly understood. Thus, there is a need to understand the molecular basis of chemoreception in tephritid fruit flies.

Currently, understanding of the molecular components in the *B*. *dorsalis* chemosensory system is limited with only 10 known OBPs [24], and reports that ME increases the gene expression level of OR co-receptor [25] and that BdorCSP2 is involved in the chemoreception of Rhodojaponin-III, an antifeedant [11]. Recently, using “computational reverse chemical ecology,” one *B*. *dorsalis* OBP protein (GenBank ID: ACB56577.1) expressed in the antennae of gravid females was shown to be an attractant to semiochemicals [26]. Therefore, systematic research on chemoreception may provide valuable information that could be used for the rapid screening of potential semiochemicals. In this study, we applied a transcriptomic approach to identify a large array of candidate chemosensory genes in *B*. *dorsalis*. We used next generation sequencing (454 Life Sciences) and evaluated the presence of chemosensory genes in all the developmental stages of *B*. *dorsalis*: eggs collected within 24 h of oviposition; larvae (first, second and third instars); pupae (1 d-old, 4 d-old and 7 d-old pupae) and newly-emerged adult chemosensory tissues, including antenna, leg and head (within six days of eclosion) in a 1:1 female: male ratio. Our results show the presence of a number of chemosensory gene transcripts in *B*. *dorsalis* and the presence of antennae-specific OBPs that could be used effectively towards the control of agricultural pests.

## Materials and Methods

### Ethics Statement

The oriental fruit fly, *B*. *dorsalis* is not included in the ‘‘List of Endangered and Protected Animals in China” because it is a major fruit fly pest in tropical and sub-tropical countries. All experiments were performed in compliance with the general ethical guidelines in order to minimize pain and discomfort to the insects.

### Insect Rearing

The oriental fruit fly, *B*. *dorsalis*, was obtained from a laboratory-reared stock colony (Institute for Management of Invasive Alien Species, Zhongkai University of Agriculture and Engineering, Guangzhou, PR China) maintained at 28°C, 70% relative humidity, and a 14: 10 (L: D) photoperiod for the past 8 years. Adult flies were reared on an artificial diet mix described previously [27], and newly hatched larvae were reared on banana in the laboratory [28].

### RNA Extraction, cDNA Library Preparation and Sequencing

Total RNA was isolated from the following developmental stages: eggs collected within 24h of oviposition; larvae (first, second and third instar larvae; ratio 1: 1: 1); pupae (1d-old, 4d-old and 7d-old pupae; ratio 1: 1: 1); and adult chemosensory tissues, including antenna, leg and head (within six days of eclosion; ratio 1: 1: 1) in a 1:1 female: male ratio. All samples were snap-frozen in liquid nitrogen and stored at –80°C until total RNA was extracted. Construction of normalized cDNA libraries from the four *B*. *dorsalis* samples, and 454 pyrosequencing were carried out as follows. First, total RNA were extracted from each sample using TRIzol reagent (Life Science Technologies-Invitrogen), and the quantity and quality of RNA were assessed by spectrophotometry and gel electrophoresis. Then, mRNA was isolated from 20 μg of each total RNA using the Oligotex mRNA Mini kit (Qiagen, CA). First strand cDNA was synthetized from 1μg mRNA with SuperScript III reverse transcriptase using dT_15_VN_2_ primer (Invitrogen) under the following conditions: 5 minutes at 65°C, 2 minutes at 4°C, 1 h at 42°C and 10 min at 70°C in a PCR machine (Bio-Rad). The second strand was synthesized from 1 μl of the first strand cDNA reaction mix using DNA Ligase, DNA polymerase I and RNaseH from *E*. *coli* according to the manufacturer’s instructions (Invitrogen). T4 DNA polymerase was added and incubated for 5 min at 16°C in a PCR machine. The synthesized double stranded cDNA were purified with the QIAquick PCR Purification kit (Qiagen, Valencia, CA, USA), and the yield was determined using the TBS 380 Fluorometer (Turner Biosystems). Subsequently, cDNA was fragmented by sonication and the cDNA samples ranging in size from 100 bp to 800 bp were purified on a 2% agarose gel. Then, DNA concentration in each cDNA sample was determined using the Bioanalyzer DNA1000 kit (Agilent, USA). Each purified cDNA sample was then used to synthesize single-strand template DNA (sstDNA) libraries using the GS20 DNA Library Preparation kit (Roche Applied Science) following the manufacturer's recommendations (1/4 run for each sample). Library quality was assessed on an Agilent Bioanalyzer High Sensitivity DNA chip. Finally, each library was normalized in equimolar concentrations and diluted to 1x10^6^ molecules/μl. Emulsion based clonal amplification and sequencing were performed on the 454 Genome Sequencer FLX Titanium system according to the manufacturer’s instructions (454 Life Sciences, Branford, CT). The raw data from 454 reads are deposited in the NCBI Short Read Archive under the accession numbers SRX862648, SRX862768, SRX862771, SRX862773, respectively.

### De novo Assembly

The raw 454 sequences in SFF files were extracted using the Python script sff_extract.py developed by COMAV (http://bioinf.comav.upv.es). All the raw sequences were then processed to remove low quality and adaptor sequences using programs SeqClean Lastest86_64 [29], Newbler 2.5.3 [30] and LUCY 1.20p [31]. The resulting sequences were then screened against the NCBI UniVec database and bacterial genome sequences to remove possible contaminants. Cleaned reads shorter than 50 bp were discarded. *De novo* assembly of the high quality 454 sequences from each *B*. *dorsalis* sample was performed by Newbler version 2.5.3 using default parameters under the cDNA option (Roche, Branford, CT, USA).

### Gene Annotation

Amino acid sequences predicted from the assembled 454 sequences were compared to protein sequences in the NCBI non-redundant (nr) protein database on a local server using the BLASTALL program with the cutoff e value of 10^−5^ [32]. GO annotation was performed using Blast2GO. GO association was done by BLASTX comparison against the NCBI nr database [33,34]. To specifically annotate OBPs, CSPs, ORs, IRs, GRs and SNMPs in *B*. *dorsalis*, assembled sequences were analyzed using TBLASTN and TBLASTX programs against custom-made databases consisting of insect sequences processed using the BioEdit program [35]. Sequences whose best TBLASTN hits corresponded to OBPs, CSPs, ORs, IRs, GRs and SNMPs were then retained as candidate *B*. *dorsalis* chemosensory transcripts and their translation was manually verified and corrected if needed. Finally, families of all candidate *B*. *dorsalis* chemosensory protein sequences were analyzed on Pfam [36].

### RACE-PCR, Cloning and Sequence Analysis

To obtain the full-length coding sequences of the candidate transcripts, the SMART RACE-PCR kit (Clontech) was used with gene-specific primers (S1 Table) designed using Primer Premier 6 (PREMIER Biosoft International, CA, USA) following the manufacturer’s instructions. The amplified products were separated on a 2% agarose gel prior to purifying the products using the Agarose Gel DNA Purification Kit (TAKARA, China). The amplified fragments were then cloned into the pMD20-T vector (TAKARA, China) and sequenced from both directions. Then, open reading frames (ORF) in the assembled full-length unigenes were identified using the ORF finder (http://www.ncbi.nlm.nih.gov/gorf/gorf.html). The signal peptide of OBPs and CSPs were predicted using SignalP 4.0 [37]. Transmembrane domains of candidate ORs, IRs, GRs and SNMPs were predicted using TMHMM 2.0 [38]. The deduced protein sequences were further confirmed by searching the Pfam database with default parameters and e-value 1.0 [39]. Based on these searches, putative chemosensory genes in the *B*. *dorsalis* transcriptome were named after their *Drosophila* homologues. Transcripts with the highest similarity to the same *Drosophila* genes were differentiated with a numerical postscript (S2 Table).

### Comparative Analysis of Chemosensory Genes between *B*. *dorsalis* Developmental Stages

Following assembly, transcripts were assigned an RPKM [40] value based on the number of uniquely mapping reads aligning to each transcript using SOAP software (release 2.21). The RPKM of chemosensory gene transcripts from eggs, larvae, pupae and adults were compared the differential expression of chemosensory genes in the various developmental stages.

### Phylogenetic Analyses

Phylogenetic analyses of the *B*. *dorsalis* chemosensory genes were reconstructed based on the amino sequences after removal of the signal peptides and the data set collected from NCBI. The OBP data set contained 52 sequences from *D*. *melanogaster* [41,42,43], 16 sequences from *Ceratitis capitata* [44], 15 OBPs from *R*. *pomonella* [45] and 9 OBPs from *R*. *suavis* [46]. The CSP data set contained 47 sequences from 12 *Drosophila* sp. [41] and 5 sequences from *Glossina morsitans morsitans* [14]. The OR data set contained 63 OR sequences from *D*. *melanogaster* [47,48] and 76 ORs from *A*. *gambiae* [49]. The iGluR and IR data sets contained 66 IR sequences from *D*. *melanogaster* and 55 IR sequences from *A*. *gambiae* [50]. The SNMP data set contained 26 SNMP sequences identified in Diptera and Lepidoptera [51].

For all proteins analyzed, their respective amino acid sequences were aligned using MAFFT v.6 (E-INS-I parameter set for OBPs and CSPs; FFT-NS-2 parameter set for ORs and IRs) [52]. For each data set, the best-fit model of protein evolution was selected by MEGA 6.0 using the Akaike information criterion (the LG+I+G model of OBP data set; the LG+G model of CSP data set; the LG+I+F model of OR data set; the LG+G model of IR data set; the LG+I+G model of SNMP data set). Dendrograms were calculated using maximum likelihood analysis with MEGA 6.0 [53] with both SPR (Subtree Pruning and Regrafting) and MP (Maximum Parsimony) methods for tree topology improvement. Robustness of the branches was assessed with 1000 bootstrap pseudo-replicates. Dendrograms were viewed and edited in FigTree (http://tree.bio.ed.ac.uk/software/figtree/).

### Analysis of Chemosensory Gene Expression by RT-PCR

RT-PCR was employed to investigate and compare the expression of candidate chemosensory genes in different *B*. *dorsalis* tissues. Total RNA from different tissues was extracted as described above and treated with DNase I (TAKARA, China) to remove trace amounts of genomic DNA. Then, first strand cDNA synthesized using the First strand cDNA synthesis kit (TAKARA, China) was used as a template in PCR reactions with gene-specific primers designed using Primer3 (http://primer3.ut.ee/) [54] (S3 Table). The *B*. *dorsalis* α-tublin gene (GenBank Acc. GU269902) was used as the control [55]. Because OBPs and CSPs are not restricted to the olfactory tissues and are known to participate in other physiological functions [56,57,58,59,60,61], two schemes of RT- PCR analyses (RT-PCR and qRT-PCR) were used (S1 Text). Each RT-PCR was repeated three times using three independently isolated RNA samples. PCR amplification products were separated on a 1.5% agarose gel and verified by direct DNA sequencing (Invitrogen, China).

### qRT-PCR Analysis

qRT-PCR was used to quantify expression levels of the OBP genes and CSP genes that were antennae-rich or antennae-specific. Total RNA isolated from 100 antennae pairs and two whole bodies of male and female flies was used to synthesize first strand cDNA as described above. Primers for the OBP genes were designed using Primer3 (S3 Table). The reactions were performed with 2 μl of the cDNA as template in a LightCycler 480 System (Roche Applied Science) using the SYBR Premix EX Taq (TAKARA, China). The *B*. *dorsalis *α-tublin (α-TUB) (GenBank Acc. GU269902) was used as an internal control for normalization. Negative controls without cDNA template or transcriptase were included in each experiment. To check reproducibility, each qRT-PCR reaction had three technical replicates and three biological replicates. Relative expression of the genes in the various tissues was estimated using the 2 ^-ΔΔCT^ method [62]. Statistical analyses of the relative expression data were performed using Prism 5.0 (GraphPad Software, CA). Statistical significance of the temporal expression was analyzed by ANOVA followed by a Tukey multiple comparison test. A value of P<0.05 was considered statistically significant.

## Results

### Sequencing, Assembly and Annotation

**Fig 1:**
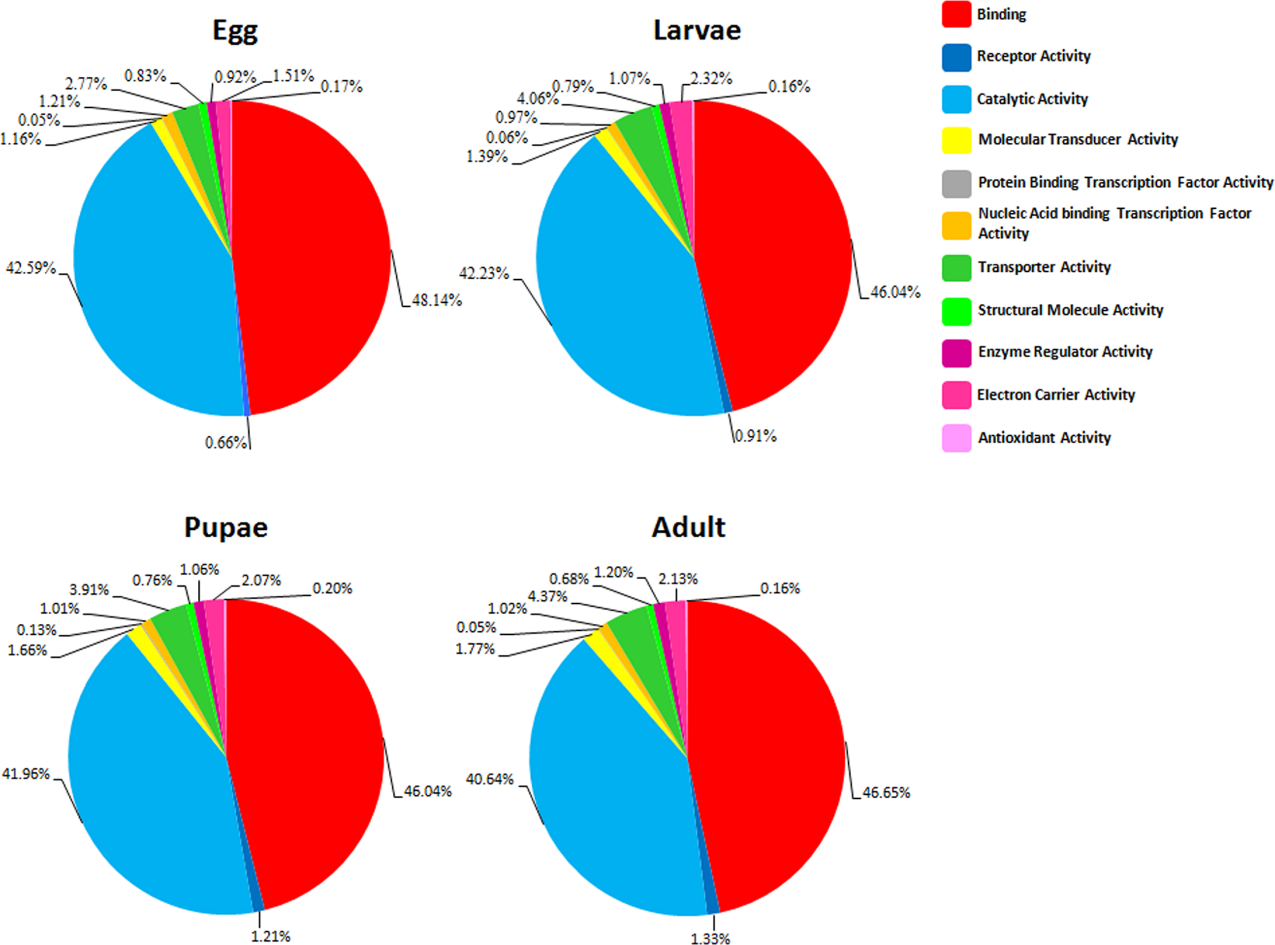
GO analysis of the molecular function category in *B*. *dorsalis* eggs, larvae, pupae and adults.

The transcriptome of *B*. *dorsalis* eggs, larvae, pupae and adult chemosensory tissues generated using the GS/FLX 454 technology yielded a total of 1,122,242 raw reads (Table 1). After assembly, 22,934 unigenes were generated in each sample (see Table 1). Among the unigenes, the majority of *B*. *dorsalis* transcripts were assigned to the “binding” and “catalytic activity” in the molecular function GO category (Fig 1) in all four developmental stages with each category having 1561 to 2280 reads (S4 Table). The category “Transporter Activity” had 117 to 201 reads while the rest including “Receptor Activity,” “Molecular Transducer Activity,” “Protein Binding Transcription Factor Activity,” “Nucleic Acid binding Transcription Factor Activity,” “Structural Molecule Activity,” “Enzyme Regulator Activity,” and “Electron Carrier Activity Antioxidant Activity” had less than 100 reads each (S4 Table).

**Table 1:** Summary of data used for transcriptome assembly.

	Eggs	Larvae	Pupae	Adult
**Raw reads**	284753	325382	217265	294842
**Clean reads**	253727	284676	187171	234173
**Clean read mean length**	362	286	282	352
**Size range (bp)**	50–627	50–658	50–565	50–782

### Identification and Characterization of Chemosensory Genes

In order to identify chemosensory genes from *B*. *dorsalis*, we performed transcriptome sequencing of four developmental stages in *B*. *dorsalis* (eggs, larvae, pupae and adults chemosensory tissues). Using homologous searches, a total of 78 putative chemosensory genes were identified, including 31 OBPs, 4 CSPs 23 ORs, 11 IRs, 6 GRs and 3 SNMPs (S5 Table and S6 Table, fasta format file in S2 Text). The complete mRNA sequences of these genes were obtained by performing RACE-PCR.

Among these were 8 OBPs (GenBank accession no. KC559112.1, KC559113.1, KC559114.1, KC559117.1, KC559118.1, KC559119.1, KC559121.1) [24] and 1 CSP (KC897022) [11,24] previously reported in *B*. *dorsalis*. However, our transcriptomes did not contain three previously reported *B*. *dorsalis* OBPs (KC559115.1, KC559116.1 and KC559120.1). The remaining OBP and CSP sequences were considered as unique and their predicted protein sequences were named as BdorOBPs and BdorCSPs, respectively.

The length of the complete BdorOBPs ranged from 134 (BdorOBP56e) to 274 amino acids (BdorOBP83ef). Prediction of signal peptide revealed that these BdorOBPs were secretory proteins, except for three (BdorOBP56e, BdorOBP57c and BdorOBP69a), which were intracellular proteins. Based on the presence of conserved cysteine profiles, 20 BdorOBPs were classified as classic OBPs, with the six conserved cysteine residues characteristic to insect OBPs [43,63]; five were identified as minus-C OBPs, which encoded putative polypeptides with four or five conserved cysteine residues [43]; two were Plus-C OBPs, with two conserved cysteines plus one proline; two were dimer OBPs, with two six-cysteine signatures; and two were atypical OBPs, with 9–10 cysteines and a long C-terminus (S5 Table). Among the classic OBPs, BdorOBP84a-1 was slightly different with one extra cysteine residue located between C5 and C6, despite having a highly conserved OBP secondary structure. Similarly, BdorOBP50c and BdorOBP50e had the general characteristics of Plus-C but also had one additional cysteine before two successive conserved cysteine residues (S5 Table).

The length of the complete BdorCSPs ranged from 111 (BdorCSP4) to 156 amino acids (BdorCSP2). Four BdorCSPs were predicted as secretory proteins and all four had the characteristics of insect CSP gene families, with four high cysteine profiles.

Among the ORs, BdorORCO, BdorOR7a-1 and BdorOR63a-2a belonged to the highly conserved OR family with seven transmembrane domains, which is a characteristic of insect ORs, while the others belonged to a divergent member of the OR family with 4, 5 and 8 transmembrane domains (S6 Table). Among the IRs, BdorIR41a, BdorIR75d and BdorIR100a belonged to the highly conserved ionotropic glutamate receptors (iGluRs) family with three transmembrane domains (S6 Table), and others belonged to a divergent group of IRs. Bioinformatic analysis identified 6 candidate GRs. The insect GRs contained seven transmembrane domains. TMHMM2.0 predicted 1 candidate GR (BdorGr63a) with seven transmembrane domains (S6 Table). Not surprisingly, three SNMP sequences, BdorSNMP1-1, BdorSNMP1-2 and BdorSNMP2 that shared high amino acid identity (86 to 91%) with the conserved insect CD36 family were also identified.

### Expression profile of Chemosensory Genes

**Fig 2:**
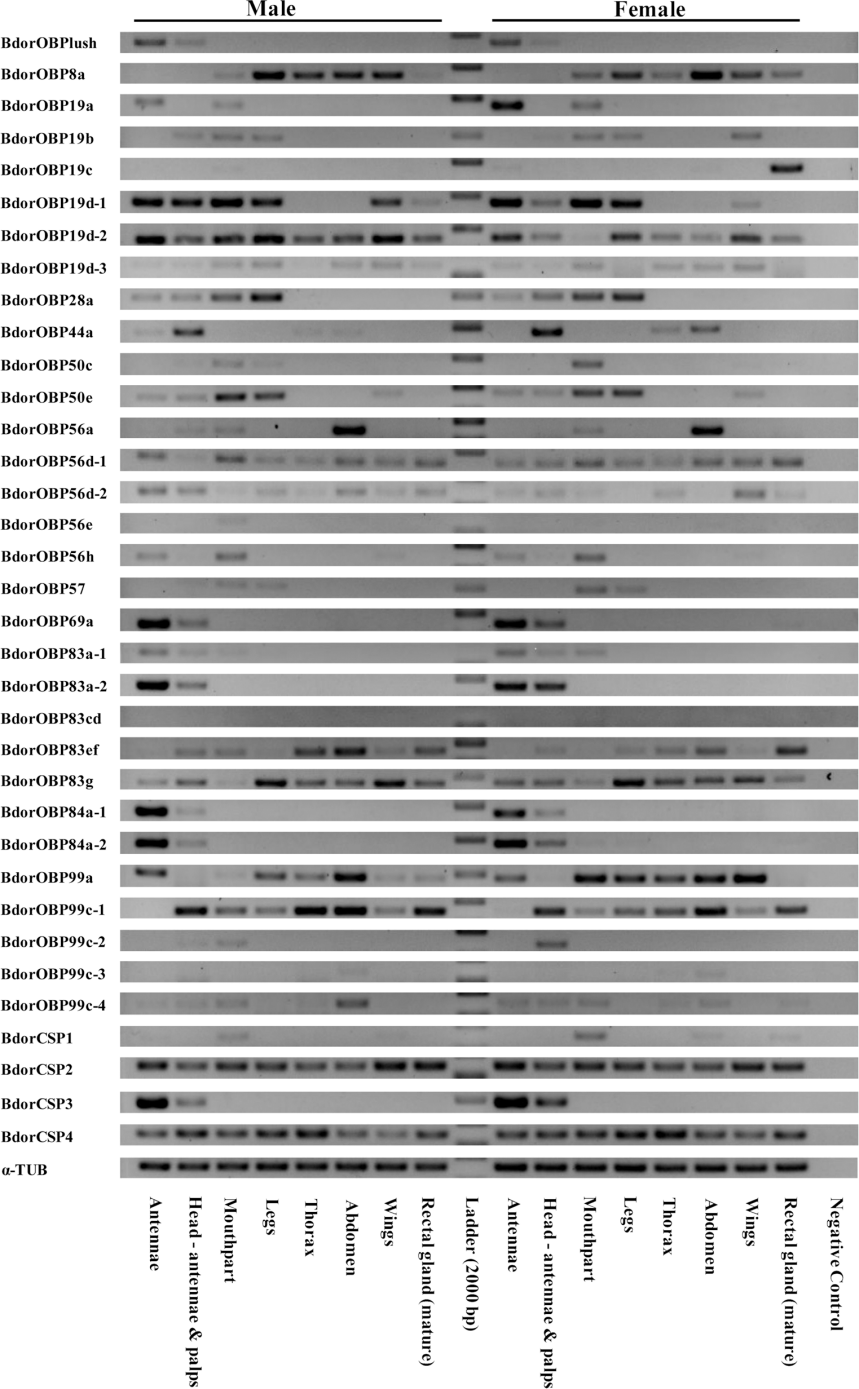
Tissue- and sex- specific expression of candidate *B*. *dorsalis* OBP and CSP genes.

**Fig 3:**
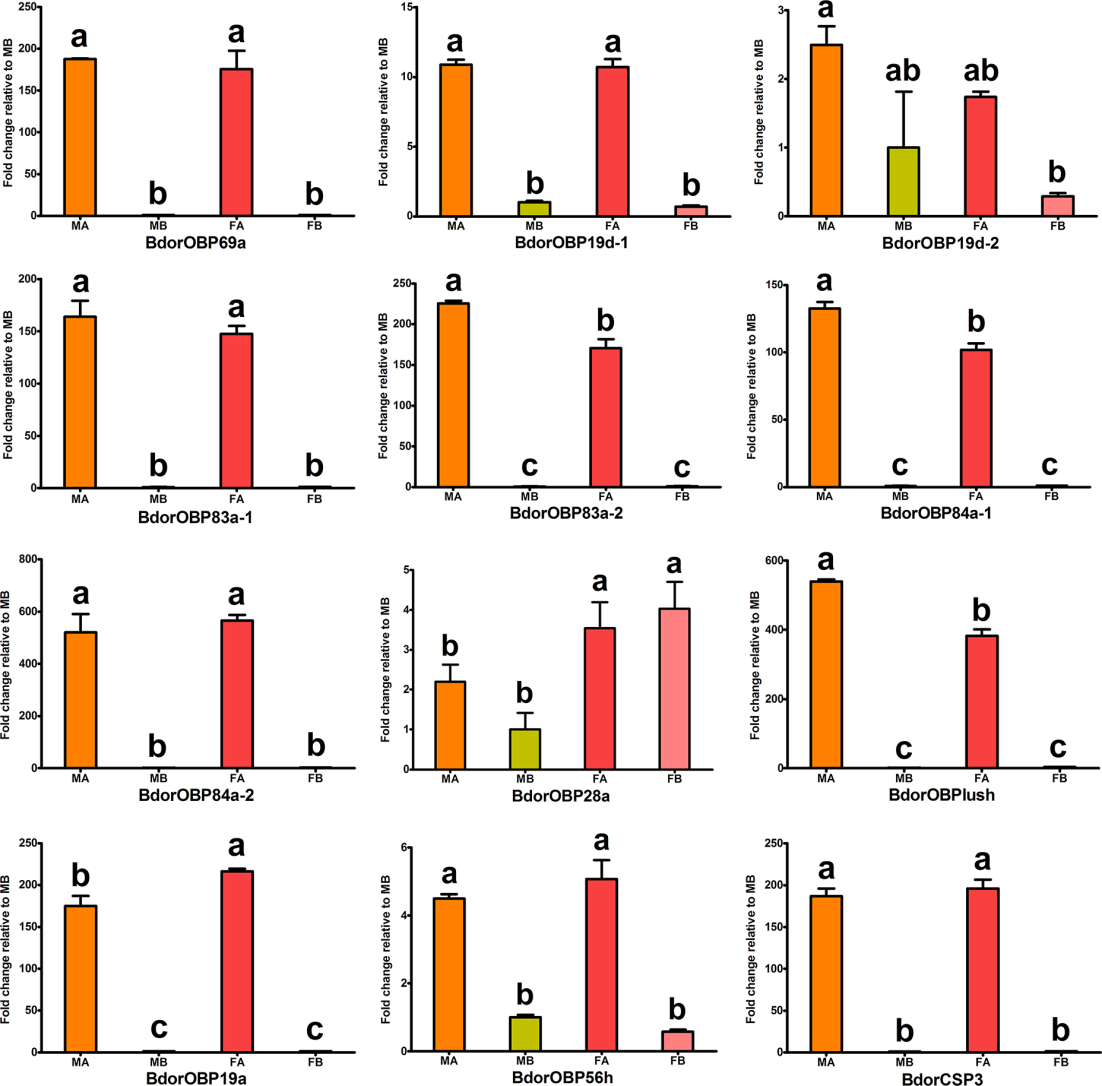
Transcript levels of *B*. *dorsalis* OBPs and CSPs in different tissues measured by RT-qPCR. MA: male antennae, MB: male body, FA: female antennae, FB: female body. Error bars represent standard error. Different letters (a, b) above each bar denote significant differences between samples (p<0.05).

Based on the RPKM value, it was evident that all these candidate odorant-binding proteins were expressed at a high level, while the candidate chemosensory membrane proteins were expressed at a low level in the different development stages (S7 Table). To provide functional clues, the tissue distribution of the OBP and CSP gene transcripts were examined using semi-quantitative RT-PCR (Fig 2). The results showed the robust expression of 9 OBP genes (BdorOBPlush, BdorOBP19a, BdorOBP56h, BdorOBP69a, BdorOBP83a-1, BdorOBP83a-2, BdorOBP84a-1 and BdorOBP84a-2) and BdorCSP3 exclusively in the male and female antennae. Although the PBRP homologs with *D*. *melanogaster*, BdorOBP19d-1, BdorOBP19d-2 and BdorOBP28, were present in the antennae, they were also abundant in other tissues (Fig 2). Based on such differential expression of these OBPs and CSPs in male and female antennae, we presume that these OBPs in males and females may be involved in detecting sex pheromones while in females they may play important roles in locating suitable host plants and oviposition sites. We further quantified the *B*. *dorsalis* OBP and CSP gene transcripts using qRT-PCR and compared their expression levels in different tissues between sexes. The results suggested that 3 OBP gene transcripts (BdorOBPlush, BdorOBP83a-2, BdorOBP84a-1) were expressed higher in the male antennae than in the female antennae, while BdorOBP19a was expressed higher in the female antennae than in the male antennae (p<0.01) (Fig 3). In addition, the results suggested that 4 OBP gene transcripts BdorOBP69a, BdorOBP19d-1 and BdorOBP83a-1 and BdorCSP3 were expressed equally in adult male and female antennae. However, two OBPs, BdorOBP56e and BdorOBP83cd, were not found in adult tissues but rather in other developmental stages i.e. eggs, larvae and pupae (S1 Fig). Interestingly, BdorOBP19c appears to be a pheromonal gland OBP present in the rectal glands of mature females, but not in immature female rectal glands (Fig 2 and S1 Fig).

**Fig 4:**
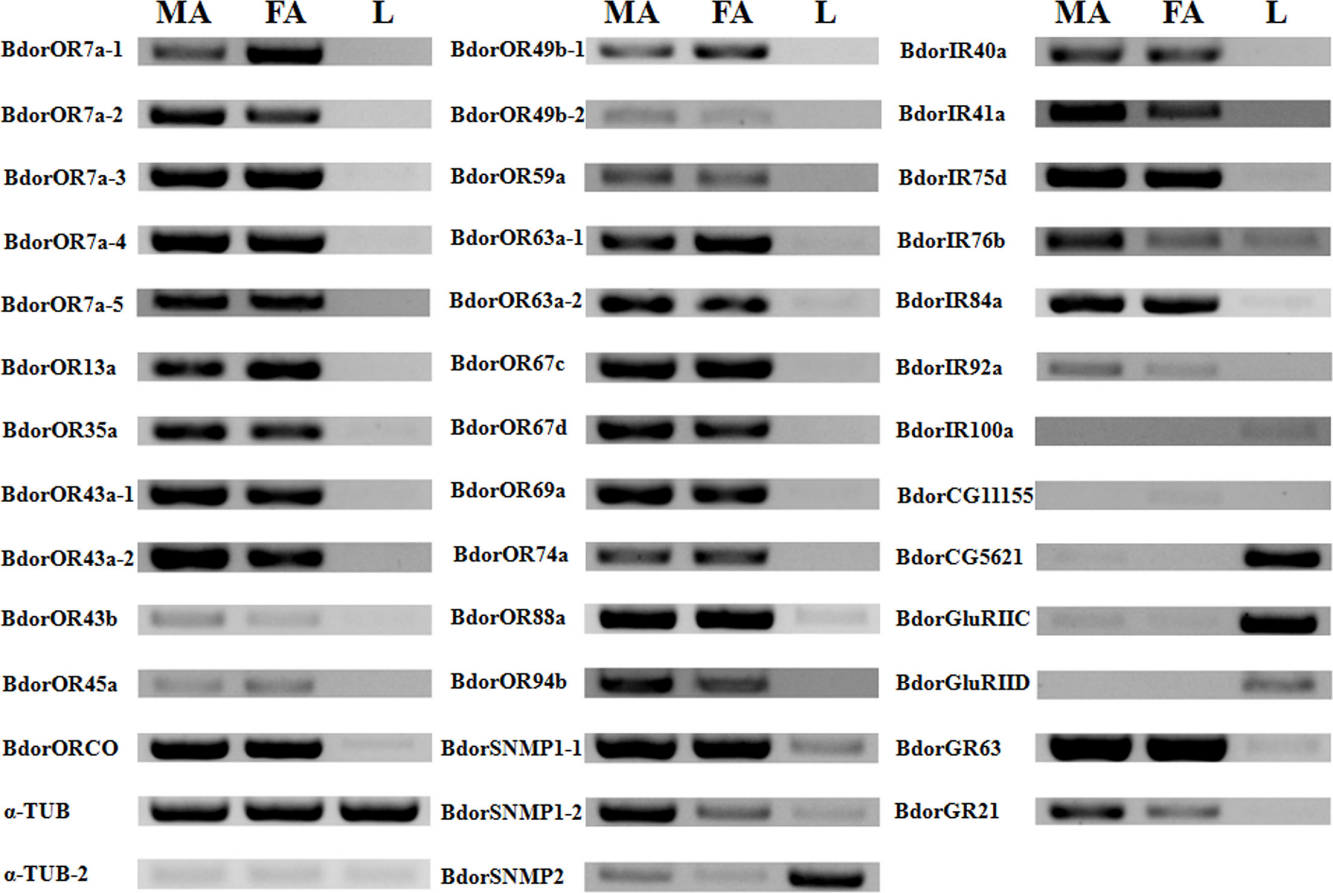
Tissue- and sex- specific expression of candidate *B*. *dorsalis* OR, IR, GR and SNMP genes. MA: male antennae, FA: female antennae, L: legs.

Expression patterns of 23 ORs, 3 SNMPs, 11 IRs and 2 GRs in male antennae, female antennae and legs were analyzed by semi-quantitative RT-PCR. The results demonstrated that all 23 ORs, 2 SNMPs (BdorSNMP1-1 and BdorSNMP1-2), 6 IRs (BdorIR40a, BdorIR41a, BdorIR75d, BdorIR76b, BdorIR84s and BdorIR92a), and 2 GRs (GR63 and GR21) were specifically expressed in antennae (Fig 4) suggesting that these receptors may play important roles in the detection of odorants. RT-PCR analyses also showed that the receptors for all 33 above proteins (ORs, SNMPs, GRs and IRs) were expressed in both sexes with some differentially expressed in male or female antennae.

### Phylogenetic Analysis of Chemosensory Genes

**Fig 5:**
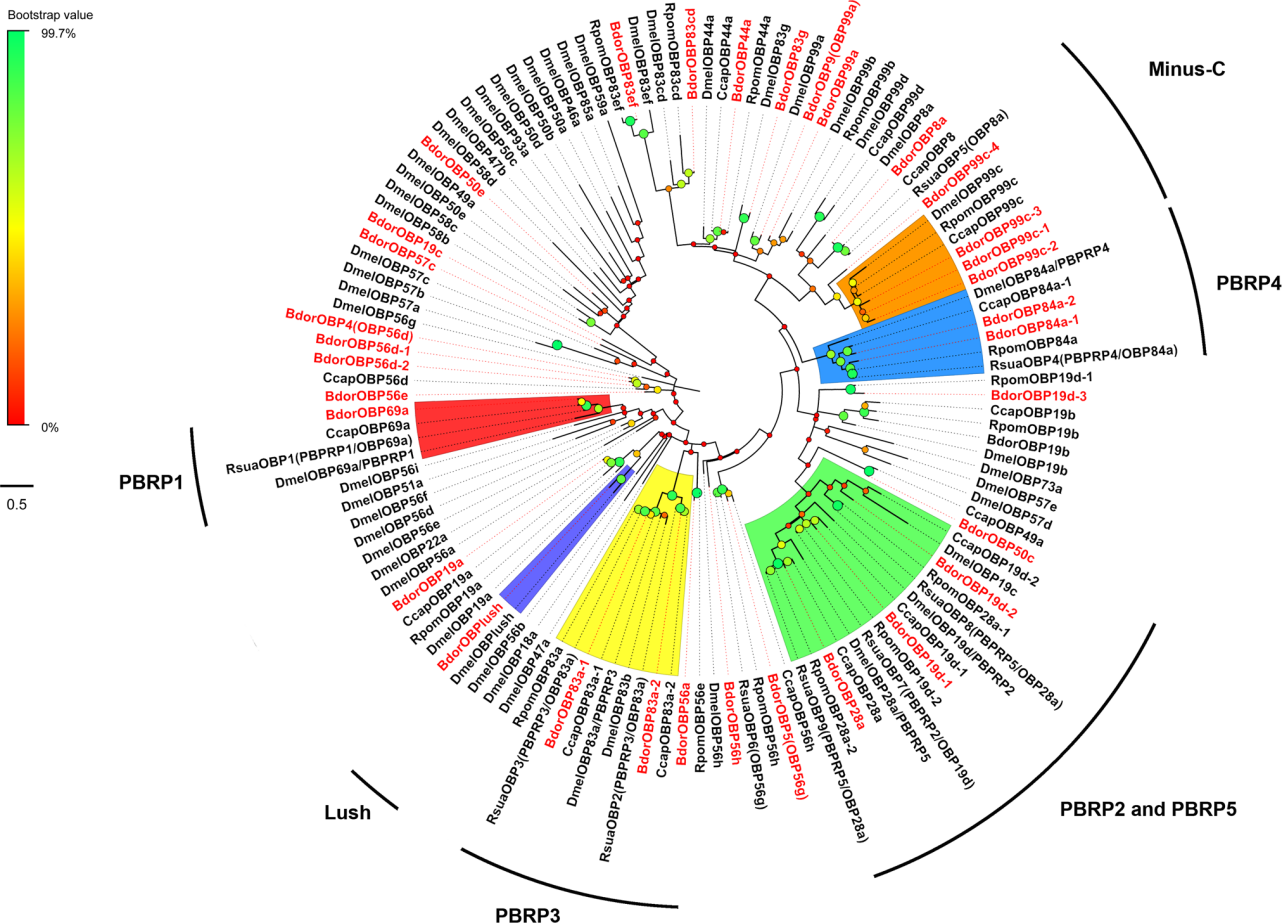
Maximum likelihood tree of candidate OBPs from *B*. *dorsalis*, *D*. *melanogaster* and other tephritids.

In order to assign putative functions to chemosensory genes, we determined the phylogenetic relationship between the 31 BdorOBPs identified in this study, and 52 OBPs previously reported in *D*. *melanogaster* and other tephritid species (the Mediterranean Fruit Fly, *C*. *capitata*; the Northern walnut husk fly, *Rhagoletis suavis*; and the apple maggot *Rhagoletis pomonella*) [41,42]. The results are presented as a Maximum Likelihood mid-point rooted tree in Fig 5. As expected, the BdorOBPs clustered together with orthologous OBPs from *Drosophila* and other tephritids with the best BLASTP hit. The classic OBPs from *B*. *dorsalis* shared phylogenetic relationships with homologs OBP from *Drosophila* and other tephritid species, which were previously classified as PBPRPs [44,64]. The nine *B*. *dorsalis* PBPRPs (BdorOBP69a, BdorOBP83a-1, BdorOBP83a-2, Bdor19d-1, Bdor19d-2, Bdor19d-3, BdorOBP28a, BdorOBP84a-1 and BdorOBP84a-2) were distributed in four well distinct clades together with homologous genes from the tephritid species. Bdor19d-1, Bdor19d-2, Bdor19d-3 and BdorOBP28a grouped with DmelOBP19d/PBPRP2 and DmelOBP28a/PBPRP5 because of the high degree of similarity between DmelOBP19d/PBPRP2 and DmelOBP28a/PBPRP5 [64]. In both cases, the four *B*. *dorsalis* Minus-C OBPs (BdorOBP99c-1, BdorOBP99c-2, BdorOBP99c-3 and BdorOBP99c-4) clustered with the *D*. *melanogaster* and other tephritid Minus-C ortholog clade. BdorOBPlush and BdorOBP19a, which had robust expression levels in the antennae, also clustered together with homologous OBPs.

**Fig 6:**
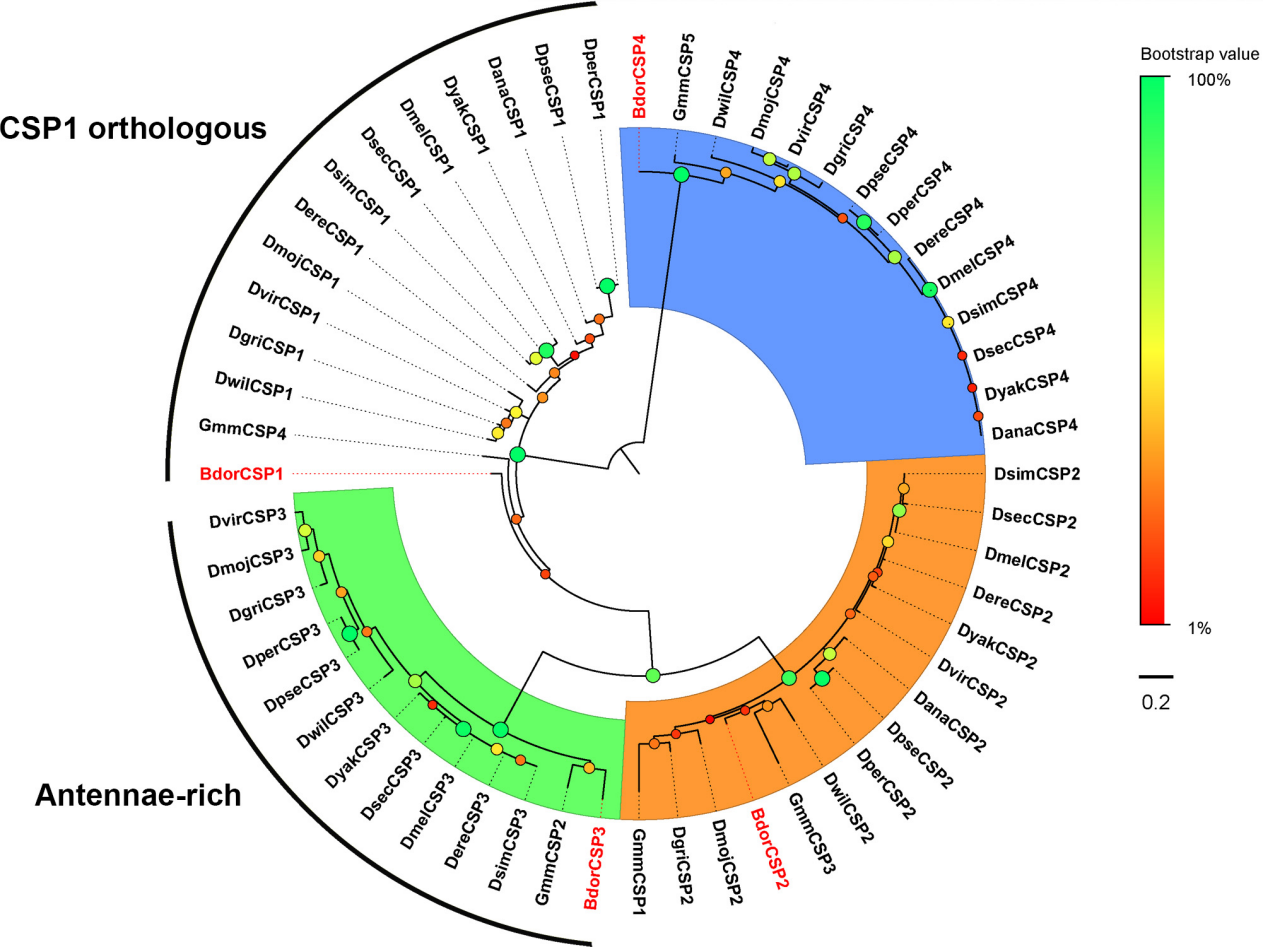
Maximum likelihood tree of candidate CSPs from *B*. *dorsalis* and other *Drosophila*.

Further, the phylogenetic relationship between four BdorCSPs identified in this study, and previously reported 5 GmmCSPs [14] and 47 *D*. *melanogaster* CSPs [41] are shown in the Maximum Likelihood mid-point rooted tree in Fig 6. The four BdorCSPs (BdorCSP1-4) were distributed separately into four well distinct clades together with tephritid homologs. Interestingly, the antenna-specific BdorCSP3 appeared to be closely related to GmmCSP2, which was previously reported to be involved in olfaction [14]. Moreover, the two antenna-specific CSPs BdorCSP3 and GmmCSP2 were orthologs of DmelCSP3 (DmelA10 or OS-D), which is expressed in the antennal segment 3 of *D*. *melanogaster* sensillum coeloconicum [65,66]. BdorCSP1, GmmCSP4 and 12 Drosophila CSPs diverged from the rest of the genes.

**Fig 7:**
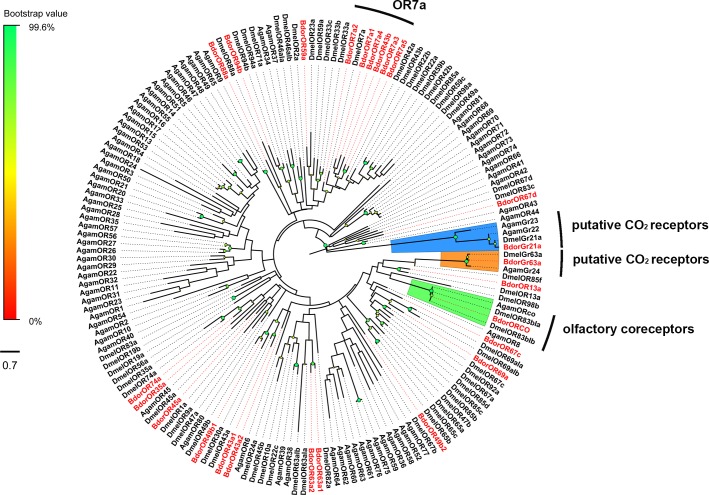
Maximum likelihood tree of candidate ORs and GRs from *B*. *dorsalis*, *D*. *melanogaster* and *A*. *gambiae*.

Sequence similarity analysis of the *B*. *dorsalis* ORs revealed that BdorORCO grouped into a conserved clade containing olfactory co-receptors from *A*. *gambiae* and *D*. *melanogaster* (Fig 7). The other BdorORs clustered together with the *Drosophila* ORs that produced the best BLASTX hits. In addition, BdorGR21 and BdorGR63 were found in a clade with two carbon dioxide receptors from *A*. *gambiae* [67,68] and *D*. *melanogaster* [69,70], respectively (Fig 7).

**Fig 8:**
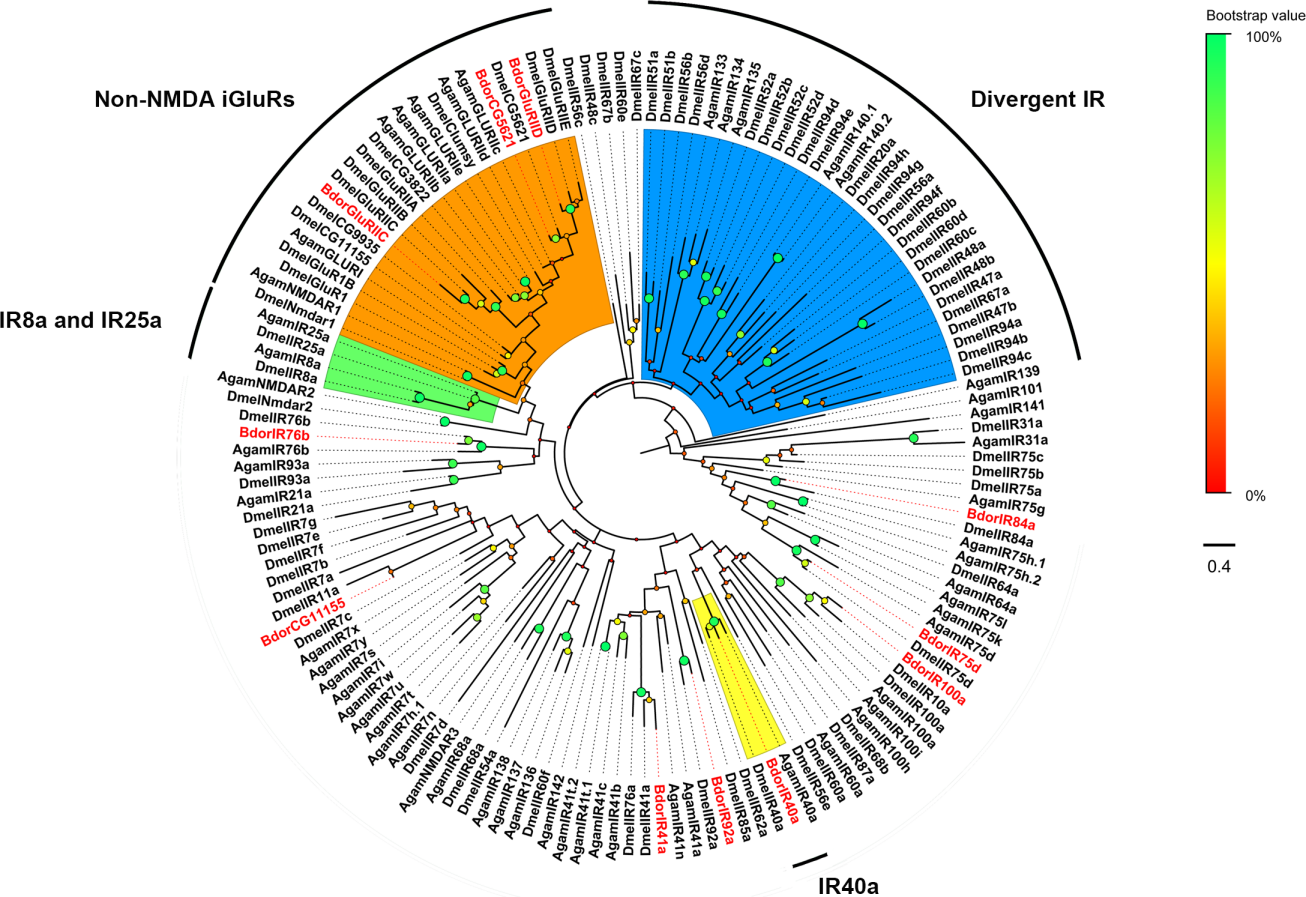
Maximum likelihood tree of candidate IRs from *B*. *dorsalis*, *D*. *melanogaster* and *A*. *gambiae*.

Phylogenetic analysis of all IRs generated an ML tree (Fig 8), which showed clustering of BdorIRs with ‘‘divergent IR” or “Non-NMDA iGluRs” in the clade. A limitation here is the possible identification of false positives due to low expression of some transcripts in the whole insect. Such low expression may have resulted in the lack of identification of two conserved IRs (IR25 and IR8a) typically found in all insects.

**Fig 9:**
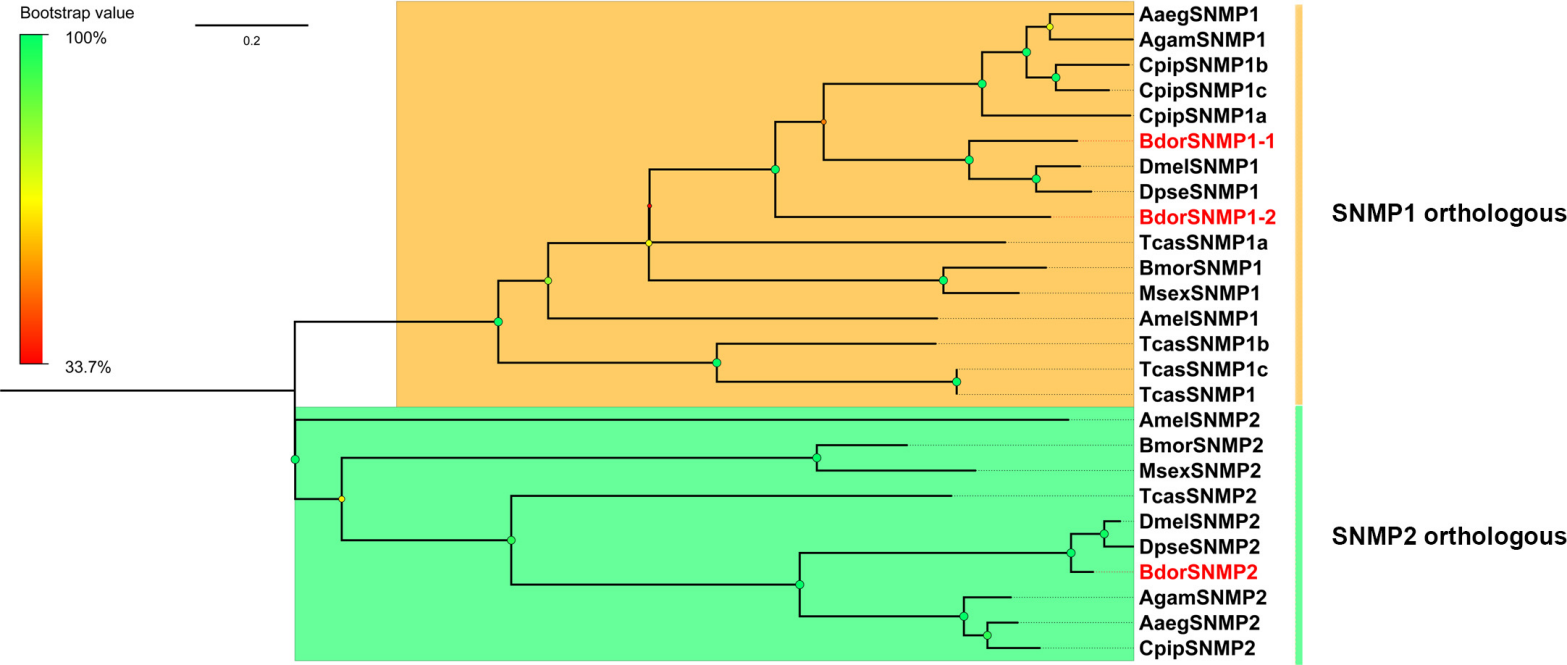
Maximum likelihood tree of candidate SNMPs from *B*. *dorsalis* and other insects.

The BdorSNMPs grouped together with orthologs from *Drosophila* sp. (*D*. *melanogaster* and *D*. *pseudoobscura*) (Fig 9), BdorSNMP1-1 and BdorSNMP1-2 were found in a clade with *Drosophila* SNMP1, and BdorSNMP2 was found in a clade with SNMP2 from *Drosophila* sp.

## Discussion

The *B*. *dorsalis* transcriptomes reported thus far have focused on genes related to development [71,72], digestion, detoxification[73,74,75], sexual dimorphism and reproduction [76]. However, the *B*. *dorsalis* chemosensory genes have not been characterized previously at the transcriptome level. To identify the chemosensory genes in *B*. *dorsalis*, we sequenced the transcriptome of all the developmental stages including adult chemosensory tissues. Our study identified 31 OBPs, 4 CSPs 23 ORs, 11 IRs, 6 GRs and 3 SNMPs. It is noteworthy to mention that in addition to the new OBPs and new CSPs in our study, we also identified chemosensory membrane proteins (23 ORs, 11 IRs, 6 GRs and 3 SNMPs) previously not reported. Interestingly, we did not identify any IR-coreceptors (IR8a and IR25a) likely due to the limited transcriptome coverage in our study (one total Run for RNA-seq) and/or the low abundance of IR-coreceptors in this species. To some degree, the RNA-seq data would only provide limited reference information.

Based on current research, insect OBPs and CSPs have been assigned two different functions: olfaction and non-olfaction [61]. Most olfaction-related OBPs and CSPs are abundant in the sensillum lymph of olfactory organs (antennae and maxillary palp), and play a critical role as solubilizers and/or carriers of odorants and pheromones[63,64,77–82]. However, non-olfaction-related OBPs and CSPs have been found in the pheromone gland secretions involved in the delivery of semiochemicals (example, pheromones)[83–89], and secretions of the reproductive organs involved in egg and embryo development[90,91]. The expression patterns of both olfaction and non-olfaction related *B*. *dorsalis* OBP and CSP genes determined in this study could provide insights to the functions of these proteins. Importantly, nine BdorOBPs and BdorCSP3 showed the highest expression in antenna, suggesting an olfactory role for these genes with antenna being the major olfactory organ. The PBRP homologs, BdorOBP19d-1, BdorOBP19d-2 and BdorOBP28, were also highly expressed in the antennae and in non-chemosensory tissues (Fig 2) indicating that these OBPs may also have other non-olfactory functions. qRT-PCR analyses of antennae-rich or antennae-specific genes in adult males and females revealed that BdorOBPlush, BdorOBP83a-2 and BdorOBP84a-1 were expressed higher in the male antennae than in female antennae (Fig 3). These genes are more likely to play a role in the odorant perception of sex pheromones or as male attractants.

Recently, it was shown that the GOBP protein (100% amino acid sequence similarity with BdorOBP84a-1) extracted from gravid *B*. *dorsalis* female antennae had a high affinity to the male attractant, ME, which is used widely as a lure along with insecticides to attract insects for pest control. However, ME is a powerful attractant for *B*. *dorsalis* males rather than females. In contrast, BdorOBP19a was expressed higher in the female antennae (Fig 3) suggesting that it could play a role in odorant perception of sex pheromone or oviposition behavior. This OBP can be a potential target to attract female *B*. *dorsalis* flies.

In general, flies express four CSPs and the *D*. *melanogaster* CSP3 homolog has been demonstrated to play a role in the detection of odorants in *Glossina morsitans morsitans* [14]. We found that BdorCSP3 had antennae-specific expression profile, which may be critical for the perception of some host volatiles as reported previously through binding assays and RNAi coupled with electrophysiological tests [10]. In addition, BdorOBP56e was expressed only in specific developmental stages (eggs to 1d-pupae) (S1 Fig) suggesting its involvement in egg and pupal development. Interestingly, BdorOBP19c was specifically expressed in the mature female pheromone gland rather than immature pheromone gland indicating a possible involvement of this OBP in the binding and transportation of female specific sex pheromones and their precursors [61].

Compared to OBP and CSP transcripts, ORs are highly restricted to the antennae and are expressed in low levels. Consistently, we found that all the *B*. *dorsalis* ORs were also specifically expressed in the antennae of both sexes. However, a new member of the chemosensory receptor family, Ionotropic receptor, did not follow this expression pattern but was expressed in other tissues. Among 11 BdorIRs we discovered, 6 IRs (BdorIR40a, BdorIR41a, BdorIR75d, BdorIR76b, BdorIR84s and BdorIR92a) were specifically expressed in the antennae of both sexes suggesting that they could be involved in odorant detection.

Moreover, phylogenetic analysis indicated clustering of BdorIR40a with *A*. *gambiae* and *D*. *melanogaster* IR40a, which could directly detect DEET and is a target of insect repellents [92]. Thus, BdorIR40a could be a good target for pest control. In addition, the extent of this shared “DEET repellency” could account for the low degree of species-specific diversity of IRs among the dipterans analyzed here. In addition, two GRs (BdorGR63 and BdorGR21) homologous to insect CO_2_ receptors [67–70] were specifically expressed in the antennae of both sexes suggesting their involvement in the detection of CO_2_. Interestingly, two SNMP transcripts, BdorSNMP1-1 and BdorSNMP1-2, displayed a high antennae biased expression profile while BdorSNMP2 was highly expressed in the legs. It is plausible that the two SNMP1s are important for chemosensory function, while SNMP2 may have functions in addition to chemosensation.

## Conclusions

By sequencing the transcriptome from various *B*. *dorsalis* developmental stages, we identified a variety of genes potentially involved in olfactory signal detection and pheromone biosynthesis in this notorious fruit fly pest. Expression profile analysis revealed that 9 OBPs, 1 CSPs, 23 ORs, 2 SNMPs, 6 IRs and 2 GRs are specifically or mainly expressed in the male and female antennae. The antennae-enriched OBPs, CSPs, ORs, IRs and SNMPs could play a role in the detection of pheromones and general odorants. The identified OBP (BdorOBP19c) could play a role in the binding and transportation of female specific sex pheromones and their precursors. The chemosensory genes identified in our study will provide the basis for functional studies.

## Supporting Information

S1 Table Primers used in RACE. (XLSX)

S2 Table BLASTX analyses of *B. dorsalis* chemosensory genes compared to *D*. *melanogaster* peptide database and suggested chemosensory gene names. (XLSX)

S3 Table Primers used in RT-PCR and qRT-PCR. (XLSX)

S4 Table GO analyses of the transcriptome data from different *B*. *dorsali* developmental stages. (XLSX)

S5 Table List of *B*. *dorsalis* chemosensory genes putatively involved in odorant binding. (XLSX)

S6 Table List of *B*. *dorsalis* chemosensory genes putatively involved in chemosensory reception. (XLSX)

S7 Table The RPKM value of candidate chemosensory genes in different *B*. *dorsalis* development stages. (XLSX)

S1 Fig Developmental stage-specific expression of BdorOBP19c,BdorOBP56e and BdorOBP83cd. (TIF)

S1 Text Two schemes of RT- PCR analysis. (DOCX)

S2 Text Chemosensory sequences identified in *B*. *dorsalis* in Fasta format. (DOCX)
