# Supplementary figures and images for: Discovery of Chemosensory Genes in the Oriental Fruit Fly, Bactrocera dorsalis
Source: PLoS One. 2015 Jun 12;10(6):e0129794. doi: 10.1371/journal.pone.0129794 (PMC4466378; doi:10.1371/journal.pone.0129794)

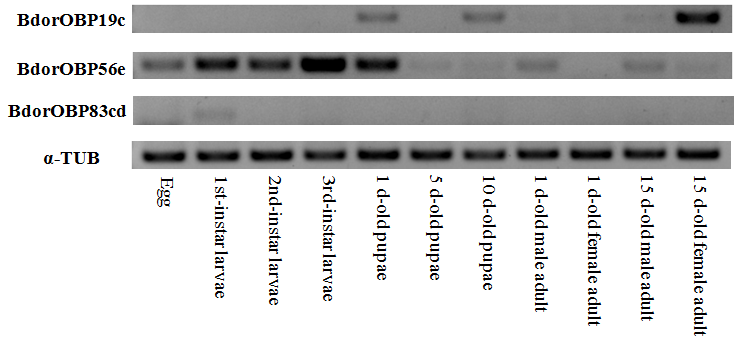

Supplement: S1 Fig — (TIF) [file pone.0129794.s008.tif]
