# Supplementary material for: Discovery of Chemosensory Genes in the Oriental Fruit Fly, Bactrocera dorsalis
Source: PLoS One. 2015 Jun 12;10(6):e0129794. doi: 10.1371/journal.pone.0129794 (PMC4466378; doi:10.1371/journal.pone.0129794)
Supplement: S1 Text — (DOCX) [file pone.0129794.s009.docx]

1. For RT-PCR analysis of OBPs and CSPs, RT-PCR was performed using cDNAs prepared from antennae (100 pairs), heads with antennae and palps (20), mouthpart (40), legs (25sets), thorax (10), abdomen (10), wings (40 pairs), and genitals (40). Amplification was performed by denaturing 95℃ for 3 min, followed by 34 cycles of 95℃ for 30 s, 59℃ for 30 s, 72℃ for 1min, with a final extension at 72℃ for 10 min.
2. For RT-PCR analysis of ORs, SNMPs and IRs, RT-PCR was performed using cDNAs prepared from male antennae (100 pairs), female antennae (100 pairs) and legs (male and female mixture, 25set). Legs were used as a control to verify that the candidate receptors were antennae enriched. Amplification was performed by denaturing 95℃ for 3 min, followed by 34 cycles of 95℃ for 30 s, 59℃ for 50 s; 72℃ for 1min, with a final extension at 72℃ for 10 min. The cycle number was reduced to 28 and 30 for α-tublin and BdorOrco amplifications because of their high expression level.
