# Supplementary material for: Discovery of Chemosensory Genes in the Oriental Fruit Fly, Bactrocera dorsalis
Source: PLoS One. 2015 Jun 12;10(6):e0129794. doi: 10.1371/journal.pone.0129794 (PMC4466378; doi:10.1371/journal.pone.0129794)
Supplement: S2 Text — (DOCX) [file pone.0129794.s010.docx]

>BdorOBPlush

ATGTATTTGAAAATAAATGCATTAAAATATTTTTTAACTCTCTTAGCGTGCACAGCTGTTAGTGCGGTAACAATGCAGCAATTTGAAACCTCTCTAGACATGATGCGCAATGGTTGCGCACCCAAATTCAAAATCGCCACCGAAATTTTGGATAATTTAAGAGCTGGCGAATTTATCGAAAACAATGGCGACTTGAAGTGTTATACCAGATGCATAGCTCAGCTGGCAGGCACGGTAACTAAAAAGGGCGATTTTAGCGTGCAAAAAGCGTTAGCACAAATTCCAATTATATTGCCACCTGAGATGCAGGACCCAGCTAAGGAAGCGTTGAATGCTTGTAAGGATGTGCAAAAAAACTATAAGGAATCTTGCGATAAGGTCTTCTACACAACGAAATGTGTGCGCGACTTTGATCCAGCTACATTTAAATTTCCATAA

>BdorOBP8a

ATGCCACAAGCAATGAAGTGCACAGCGTTCGTTTTCATTATTCTCTTCGCTGCATTATTCGCGCACATCAACGCCGATTACGAGGAGAAGACGGAGGATGATTTCTTAAGCGCCGGCGAGCGTTGTTTCCAGCGTGAGCGTTTGGCCGCCTCCTATCAGCGTCGCTTCGACAACTTTGATTATCCGGACGAGGAGCCGGTCCAGCGTTATGTGCACTGCATTTGGACAGAGTTGAAATTGTGGAATGATCGCACCGGTTTCAACGTCGAACACATAGCGGCACTTTATCGCGATAAGGCGAATACGGAAGTGTTGGTGCCAATATTGAGCGACTGCAATCGGAATGCACAGAATGAGCCGACCCTCAAGTGGTGTTACAGGGCCTTCAAGTGTGTGCTAAACAGTCGGGTGGGTCAGTGGTTCAAGGAGGATGTCGGTCGCAAATTGGAAGAGCGGCGTGTGGGAAATCATGTCGCTTGA

>BdorOBP19a

ATGTTGAATAAAATAAATTCTTTCGTCCTTGCAACCGTATTCATCGCGTTGGTTTTACATTCGGATCAAGTTTCTGGCGGTGCTACCGAGGAGCAAATGATATCTGCTGGAAAGCTAATGCGAGATGTCTGTCTGCCAAAATTTAGTAAAATATCACCAGAAGTGGCAGATGGCATCAAAGAAGGTAATGTTCCAGACACGAAGGACGTAAAATGTTATATTAATTGTATCATGGAAATGATGCAAACGATGAAGAAAGGCAAGTTTCTATATGAAAGCGCATTGAAGCAAATCGATTTGCTGATGCCGGACGATTACAAGGATGATTATCGCAATGGACTAGCAAAATGCAAAGATGTAACAAGTGGCATAAAGAACAATTGCGACGCGTCGTACGCCCTTTTGATTTGTATGCGCGACAATATATCGAAGTTTCTTTTTCCTTAA

>BdorOBP19b

ATGAATAAAGTGTTAACAGTTTTTGCGGTCGTTGTTTTAATGATTGCCACAAGGAAAGTGCTGGCAGAGAAAATAATGAAGGCACCTATGGGTCTAGCGCTTGAAACCCTCGAACCGTTTGCCATGAACTGCAAGCTTAAGCCTGAAAAGGCGCATATGGAAGAGCTTATCTTGAATAAAGAGGACGCGGACTTAATAACGAAATGTTTACGTGGCTGCCTCATGGAACAGTTCCAATTGTTCGTAGAAGACAGCACTGATGTGAACACGGAGAAGTTACTGAGCTGGATGGTACTCTTGTATTCCGAAAAAATTGATGAACTCAGGGCAATATCCAATGGTTGTAATGCGAAAAATGTTGAAATGGGCATTACTGATAAATGCGAGGTGGCGCATTCGTATGCCATGTGCATGCTGAAGGAAATGAAAGAGCGCGAGTATGAGATACCCGAAGTGGAACAATGA

>BdorOBP19c

ATGGTTAAGTCAAGTAGCGCATTACTAATTGCTGGCTTTATTTGTTTGATGAGTCTACAAAGTCTCACTGCACTCAGTGAAGACGCTGACAAGTTATCTGAAAAGCGCAAGCCGCTGATGACGCGCGAGGATCCTTCAACTTTGGAAGATTACAAACGCACCAAACGCCAATTGCCACAACCGCTGCAAGAGTTTCAAGATTTTGTAACAACTTCGAAGACCCAATGCGCAAAGGAAATGAATATTAATCCGAATGAATTGCAAAAATCGTTGCTTTACGAGGATCAACCGACTTCCATGGAGAAGTGTATGATGGAGTGTGTTTTGAAGCGCATAGAAGTGATGAGCAAAGACGACACGTTGTCGACGACGACGATTGGACACATCGCTGACATCATTGGCGATAATAATGCCTTAATAACGTCAATTGCTATGGCCTCAGCTGAGAATTGCAAAAAGTTCATCACTGCGGAAGATTCCTGTGAGCGCGCTTTTCAAATTAATAAATGCATCGCCGCTGAAATGAAAATGCGCAAAATTAAGCTCATATATTAG

>BdorOBP19d-1

ATGAAGATCCTTAATATTTGTTTGATTGTTTGTGTAGCGCTTATATCAAACGCTAAGTGCAATTACGAAGAGGCGAAAGCAGTTGCAAATGAGTGTAAGGAAGAAGTTGGTGCAACAGATGATGAGCTTGAGACGATTTTGAAGATGGAAGCAGCTGAGACTACGACGGAAAAATGTCTGGGTGCATGCGTCATGAAAAGATTCGGTGCGATGAATGGTGATGGTAAGTTTGATAGAGAAAAAGCCATGGAGATTTTGGCAATTATCGCCGACGGCAATGATGAGCAACATGCCTTGGGTGTGGAAGTTCTAGACGCATGTGCTGATATTGACGTAAACGAAGATCATTGTGAAGCAGCTGAGGAATATCGCACTTGCATGCACGCCAAAGCAAAGGAAATTGGCTTTGTAGTGGGGCGTGTTTAA

>BdorOBP19d-2

ATGAAGTATTTCGTAGTCTTCCTGGCTATCTGCAGCTTTGCTATCTCCTTTAGTGAGGCAGATGAATTTGGGGAGAAAGTTAAGAAAATCGCTGAAGAATGTAAAGGGCAAGTTGGTGCAAGTGATGACGATGTAGCACGATTGTTCAAATATGAGCCCGCCGCTAATGACAAAGCAAAGTGTCTTACTGCTTGCACTATGAAAAAACTCGGCACTATGGATGAAAATAACAAAGTGGTCGAAGCTGGAGCTATAGCTTACATAAAGCAGCTCTCCGGTGGCGATGCAGAGTTTGAGAAGTTGAGCTTGGAGACTTATAACGAATGCAAGAGCACCCCGGAAAGTAGCAATGAATGTGAGTACGCCGAAGCTTTTCGTCAATGCGTTCTCGAGAGTGCGAAGTCCAAAGGCCTTAAGATTTTACCTCAAGTTTAA

>BdorOBP19d-3

ATGGAAATTCTTATCGTGCTACCGATTTTAGCAGTGTCTTCAGTATTCACAATAACAATAGCTGCTGCTGAATCTGAACCGCCACATTATAGTTCACTTCGAGTTATGGCCGAGGCTGCGATTGAAGATTGCTACGAGGATTCGGCTCAGAGTGTCAAAGTGCAGATAACCGATGAGAGTTTTGATGAAATATTGAAGGGTTCTCGCACTAATTTATCTCACAATGCGAAGTGTTTACGATACTGTATTATGCGCAAAAATGGCTTGTTAAGTATGGATAACTCAATCGATGAGGAAAACATTTTGCAAATATTTGGAATAATACATCCTCAAATTAAAAAGGATTCACTACTGGATGTGCTACATAAATGTGCGCGTGAAACGGATAAGCAAACTGATAATTGTGAGCGCGCTTTCGTGGCAACCGGCTGTATTCTCCGAGAGCTGCAAGCAGATGGTGTAACCGATATTTAA

>BdorOBP28a

ATGGCCAAACTCATCCTATTCGCCGCCTTATGTATTTTGAGTGCCGCTGTCTCCAATGCTGCTTTCAACAAAGAAGAGGCTATAAAGAATTTTATGACTAAAGCGGAGGAGTGCCGTGGCGAAGTGGGTGCTGCTGACTCTGACATTCAGAACATAGTCGCAAAACTACCAGCGGCCAGTAAAGAAGGCAAATGCTTGCACTCCTGTTTGATGAAAAAATATGGCGCGATGGATAGTAATGGCAAGTTTGTCAAGTCGGTCGCCGATCAGCATGCACAGGACTTCACTGACGGTGATGCAGATAAATTGAAGACAGCTCGTGAAATTATCGACGCTTGCGCCGATATTGCAGTACCAGATGATCATTGCGAGGCAACTGAAGTGTATGGCAAATGCTTTATGGATCAAGCGAAAGCTCACGGTATTCAAAAGTTTGACTTTTGA

>BdorOBP44a

ATGAAATACATTGTCGCTGTTTTACTCGCCGCATTGGTGGCTATGGCCGCTGCCGAGGAGTACAAGATACGCAATCAGGATGACTTGTTGAAGGCGCGCAAAGAATGCATGGAAGCGAAGAAGGTGCCCACCGAACACATTGAGAAATTCAAGAAATTCGAATTCCCCGATGATGAGGTGACGCGCTGCTACATTGAGTGCATCTTCAACAAATTCCAACTCTTCAGCCCCACCGAAGGTTTCAAGACACAAAATCTGATTGCACAACTCGGCCAAAATAAGGAGAATAAGGACGCTGTGAAGGCTGATATTGAGAAGTGCGCCGATAAGAATGAACAGAAATCGGACTCATGCACATGGGCATATCGTGGATTCAAGTGTTTCATCAGTAAGAATTTACCGCTGGTGCAGGAAAGCCTCAAGAAGAATTAA

>BdorOBP50c

ATGAAGTGTTTTCTGGTCTTTCTTTTGTTATTTGTGTTGTTGTGTGTGAATGCCTATGACTTTGATGATAGTGCTTTCAATGAATATCTCTTCAAGGAGCTGCAGTCTCATTACGAAGAAGACGAAGTCTCACATAGAACACGCCGTGAGGCCGCAGATGCCAACGAGTGTTCTAAGCGTAATTGGAAAAAGGATATGCAGTGTTGTAAGGGGGGTAATGTGAACGGTGATCAATTGGAACTCTTTAAGAGCGTGAAGAAACAATGCATTGCCGATTTGAAAGGCGAACCTGCTGATGACGCAGTAGATCCCTTCGATTGTGAGAAGATGCAACAAGTTAAAGAAAAAATGATTTGTATAACCGAATGCGTAGCGAAAAGTTTTAAAAGTCTCGATGAGCATGGCGAGCTGCAACGTGATGCTATTTTGGAGGGGTTGCGAGGACAAATTGGTACGGTACAATGGAAGCTCGATGCGATTGAAGGCTATGTGGACACCTGCTTGGCTGAGGTGAAGGAGAAGCGTGAACAAAAACAAAAGGCGGGTGAATTGAAGGAGGAGGGTTGTAGTCGTTCGCCGTTGGCTTTCCATAGTTGTATGTGGCGTCAGTTTTGGAATGGTTGCCCGACAGATTTACGTGTCGACTCTCCAAAGTGCAACAAATTGCGTGAACGCGTGGCCAATGGTGATACACGCTTCTTTGGGAAACACTTCCTTCACAAATACTACCCTAATCCACACGATGAGGAGTAA

>BdorOBP50e

ATGAAGGCGTACTTTGGATATTTTGTAGTGATTTTTGTATTTGTGTGCCACGCCAGCGCGGATGATGAGACTGTGGATTGCACTAAGCCGCCACGTTTTGTTCCGCCCCACATGTGCTGTCCAGTGCCAGATGTCAGTACTGATGAACTGAAGGAGCAATGCGCGGAATATAATAAACCGCCACCACCACCGCCAATGGGACGTGGCGGTCCACCAAAGTTTGACCGTCGTCATCATCCGCATCATACACCTCCTTGTGTGATCGATTGCATTTTCAACAACACCGAAGTTATGGGGGCGAACGGCGAGCCCGATGTTGACAAATTTAGCGCGTTACTCGACACAGCCGTAAAGGATAATGAAGAAATGGCTGCAGTTATGGAAGAATCATTCGAGTCTTGCGTCGGGATGCTCAGCGAGTTGAAGGCTAAAATGGCTGAAAAGGCTAGCAAACACCCGGAATTTGCGGATAGAATGGGCAATTGTTCGCCAGTAAGCGGCATGCTGATGATGTGCGTCAATATAGAGACATTCAAAAATTGCCCAGCCTCTGCTTGGAATGACAGTACAGAATGTAATGCAACTCGTAACTTCTTCAAGCAGTGTAAATTTCCCAAAGACGGCAATTAA

>BdorOBP56a

ATGAAATCCTCTATCATCTGCTGCATACTGGCTACAGTAGTGCTCAGCCTGTGCGTTTTCAACGCGGACGCGGGTCTGCGCAAGCCCAAGAAACTCACACCGGAATTGGAAGCGAAGTTCGAAGTTTTGACCGCTTGGATTGCCTATCGTCTCAATTTGAAACACGCCAAGGAAGCTTGCGTGGGCGAATATGGCTTTTCGGATGAGCTTGCCACAAATCTGGTCAAAATAAAGGTGGCGAACCCAAGTGATCGCGAGAAGTGCTACGTGAACTGTCTCTACAATAAATTGGTATTCTATAAGGATGATGCCATCAACAAACAGGCCATGAAGGAGTCGCTTTATGAAATTGTGGGTGAACAACGCTTATTGAATATTGTTGATGGTTGTCTAAACGCTGGCGGCACCAATGCATGTGATAAGGTGTATAAATTCCATGCTTGCGCCTCACCTGAATTCGACAAAGTACGCAGTGATATCTTCTTACCCGATGAATAG

>BdorOBP56d-1

ATGAAATTTTTCGCTGTTGCTGTTTTGCTAGCCTTCGTCGCCGTCGCTGTTGCGCAAGAGGGCGTCGGCAAGTTGACCGAGGAGCAGAAACAAAAGGTACACGCTGCAGCTGCTGAATGCTTTAAGGAAACCGGTGCTTCCGAAGACGCGGTTCGTGCTCTCCTCAAAGGTGATGACAGCCAAGTCGATGGCAAAGTCAAATGCTTCGCCAAGTGCACACTAGGTAAATTGGATTTACTGCAGAATGGTAAGGTCAATGAGGAAAAGGTACAGAACATTTTGGGCAAATTGATCGGCGAGGAGAAAGCTAAGGCTGCTCAAGCTAAGTGCAATGGTTTGAAGGGTACCGACGAATGTGACACCGCTTACCAAATTCGCCAATGTTATGCAGCTGGGCATGAGAGCTTTGTTTTTTAA

>BdorOBP56d-2

ATGAAATTCTTCGCTGTTGCTGTTTTGCTAGCCTTTGTCGCCGTCGCTGCTGCGCAAGAAGGCGTCGGCAAGTTGACCGAAGAGCAGAAACAAAAGGCTCGCGCTTTGGGCACTGAATGCTTGAAAGAAACCGGTGCTTCCGAAGAGGCGATTCGTGCTCTCATCAAAGGTGATGACAGTCAAGTTGATGGCAAAGTCAAATGCTTCTCCAAGTGCATGCAAGAGAAATTGGGTTTTGTAGAGAATGGTAAGGTCAATGAGGAAAAGGTACAGAACTTTTTGGGCAAATTGATCGGCGAGGAGAATGCTAAAGCTACTCAAGCTAAGTGCAATGATTTGAAGGGTACCGACGAATGTGACACCGCCTTCCAAATTCGCCAATGTTATGCAGCTGGGCACGAGGGCTTGGATTTTTAA

>BdorOBP56e

ATGAAATCGTATAATTTTGCTAGCCGCTTTAATTTCACTCAGCGTCTGTCGCGGCTCTATGAGTTGAAGCCCAATAAATCCACTCAAGTCCACCAGTTTTACGAGGATTGCTTGAAAGAGTCTGGAGCAAGCGCCGCGCAGTTGGATGCTTTGAAGAAGGGTGATTTCAACGCGGTCGATGATAAAGCGAAATGTTTTCTAAAATGTCTGCAGAACAAGAAAGGAATTCTAGAAAATGGCGTGCCAAATGAAGCAGCTATCCATAAGGTAATGACGCCGGCGGTTGGTAATAGTCCACCAAAAAATACGCTAGCCAAATGTAATGGCTTGAAGGGTGCCAACGAGTGTGATACCGCCTTTCAGATATATAAGTGCTATCGGCAAGAGCATGTTGGCTTGATTTGA

>BdorOBP56h

ATGCAAAAGTTCCACATTTTGACAATAATTGCGGCTTTAGTCACGCTGGCTGTGTGTCAATTGCCAGCGGATTTAGAGAAATTCCACAAAGCTTGTATGGACGAAGCCAAAGTCACGGATGAGCAGATGAGGCAATTCTTTCAAAACGGCATGAAAGCTAGCGATGCCACAGAGAATATAAAGTGTCAAATGAAATGTATGATGCAAAAGCAGGGCATATGGAAGGATGGCGTCTTTGATGCTGATGCCAAAATTAAGGAATTAGTGCAAAATCCAAAATTTAAAGGAAAGGAAGCAGACCTAACAAAAGCTATAAATAATTGCAAGAATGAGAAAGGCGCAAATGAATGTGATACAGTATTTAAGATTAGCATGTGTATCAAGGAATTCATGACACAGAATAATCTCTAA

>BdorOBP57c

ATGTATCAATTTGGTGCACATGAAAAACGCGCAACAACAACAGCAACAATGAGCGCTACTGTGTTGGCTGCGGGCGGCGGCAAAGGAAAGTCAATACCTGGACTCACTTGGTTGGTGTTGTTGGCGGTTATTGTGGTATTTGCGTTGCCACCTGGCGCCGTCGCATTGACACCAACCGCACCCACCCGCAGCTTCGTGGAGGCCTGCCAAGTAAAACACAACATTACCTTGCAGGAACTGGATGAATTTCCCACCGATCCGAGTCCCGAAGATATCGACATGAAATTCAAATGTTATGCCGATTGTCTGCTAAATGGCATGGGCTTCATGGATTCAAATGGAAAATTGGATGCCGAGGGATTGCATGAATGGGGCATTCTGAACGATGAAAGCTACGAGAATATGCTCGAGTGTAAGGCCGCCAATGACATGGAGGATGATCCGTGCGAGTATAGCTTTGGCATGATGTTATGTGCGCGTATGCTCAACTCAGAGGAGGAGAACTACTACAGTGATGAGGTGGATGAAGCGGCGGAGGAAAGGCGTAGGAAATGA

>BdorOBP69a

ATGAACACCAAACAGTTTGTATTTCTCGTAATTATTTATCAATACACATTCTACACAGGAGTGACCACTCTGGAGGTGCCCAAACATATGGTATCAGGTGTAAAGAAATTGACTAATATATGCATAAAGGAATCTGGTGCCAGCGAAGATTTATTTAAGGATATCAGGGCGACGGGAGAATTGCCCAACAATCAAAATTTAAAGTGTTTCATGCATTGTGTGCTGGACAAAATTGGATTGATTGATGATGACAACATCGTGCACTTGGATAATCTCATTGAAATAATGCCACCCGATTTTGTGCCGATAATCGAACAGTTGCACACAACATGTGGCACAAAAAGTGGCGCTGATGGCTGCGAAACGGCATTTTTAACAATAGAGTGTTATATAAAAAAAGAGCCAATCATATCAAAAATGTTGTTTTCAACATTCGCGGATTGA

>BdorOBP83a-1

ATGGTTTTGACTGGCACTCGGCGCGGCCAAGCGTTCCACGCATTTCTAATTGTCGCGCTGTCGTCATCGCTGACTCTGATGCACGTACAGGCACAGGAACCGCGACGTGACGACAAATGGCCGCCACCAGCCGTCCTGAAAATGGCCAAAATATTCCACGATATTTGCGTTGAAAAAACTGGGGTCACAGAAGAGGCAATTAAGGAGTTCAGTGATGGCCAAATACACGAAGACGAGGCGCTCAAGTGCTACATGAATTGCTTGTTCCACGAAATCGATGTGGTGGACGATAACGGTGACGTTCATCTAGAGACGCTCTTCAACACCGTACCGGGTACCGTACGCAACCAACTGATAAATATGGCGAAGGAGTGTGAGCATCCAGAAGGCGATACGCTGTGCCACAAAGCATGGTGGTTCCACCAATGCTGGAAGAAGGCCGATCCAGTGCATTATTTCCTACCATAA

>BdorOBP83a-2

ATGCACTCCCGAAAGACTCTCCTGGGCACATTGCTGTGGATcGGTTTTCTGTTGAACcTTATTTGGGCGCAAAAGGAGCTGAGACGTGATGAAACCTATCCACCCCCGGAGCTGCTGAAGGAACTACGGCCTGTTCACGATTCCTGCGTTGCCAAGACGGGAGTGACAGAAGAGGCCATAAAGGAGTTCAGCGATGGGGATGTTCACGAAGACGAGCTGTTGAAGTGTTACATGTATTGTGTGTTTGAAGAAACGGATGTCtTGCATGAGGACGGTGAAGTGCATTTGGAGAAGATACTGGACAAATTACCGGAATCCATGCATGTCATAGCTTTGCATATGGGCAAGAAGTGTTTGTATCCCAAGGGCGATAATAAGTGTGAACGCGCGTTTTGGTTGCATCGCTGCTGGAAGGaAGCGGATCCAAAGCATTATTTCTTGATCTAA

>BdorOBP83cd

ATGGAATTGATTTTAATTTTCCTATTCGCAGCGACAATCATATCTCCTGCGGTGGCAGTTGTCCAACCCAATGCCGAGGAAATTCGCACACTTTCCGATTGTCTTCAAAGTTATGGCGGTATAACGGAGGAGAACTCGAAGCGTTTGGCGCGTTTTAAGGACCGGTCGGAAACGTATGAAGAGATACCATGCTTTACAAAGTGCTACATCAAGAATATGTTTAACATGTTCGACGAGTCTGTGGGATTCAACGATGAGCAGGTGATCAAGCAGTTCGGCCAGCCGCTCCACAAGGCATGCAAACATCGTATGGAACCTGCGGCAGATAGTTGTCAGCAGGCATATAATGGATTCCATTGTCTCGTCAATTTGGAGGATGACCCTTTCGTGATAATCGAGAGCATGAAAAACGTCAGCACCGAGGCGAAAACCGCCATGAAGGACTGCCTGCACCGCTTTGATCAGTACGAGTGGGAGCGCGTCAAAGACTACTCCAAAAATCCGGTACGCGAACCCATACCCTGCTTCACCAAATGCTTCATCGACCGCTTGCAACTTTATAGCCAGCAAACTCGCCAGTGGAACATACCGGCGCTAACGGCCAAATTGGGGGTGCCTGCCGCCGGAGCAAATATACAACACTGTCTGAAGCAGAGGCGCAACCGGAATGCCTGTGTGTGGATGTATCAGGAGTTCACCTGTTTTGTATTGGCTCACGATTAA

>BdorOBP83ef

ATGAATTTTCGCGAAGCGTCACAAGTGGTTTTATTAATCGCAGTCGCGTACTGTATACAAATGGTTAGCAGCGCAGCGACAACAAAGGCGGCGGAACAAGACGTCGACTCCGATACAGAGATATTACGAAAATGTTTACGCGAAGTCGGCAGCAAAGATTTGGTCGGTGAGCTACAAAAAGTTGCACGCTACTCGAAATGGACGTCGGAGGAGGTGCCCTGTTTTACACGTTGTCTCGCTTCGATGAAGCACTGGTTCGATGCGGACGAAAGCAAGTGGAACAAGCAGCAGATCGCCGATGACCTGGGCGCAGATATGTACAACTACTGCCGCTATGAGCTCGATCGCTACAACGAAGATAGTTGCGAGTTCGCATACACTGGCCTACGCTGCTTGAAACAGGCGGAGCTCTATACTCTGGAAACCTATAAGAACATTGTGAGTTGCGCCAGCGAGCTAAATGTCACCATGAAGGAACTACAAAAGTACGCCGCTTTTCCGACCAAGGAAGTTGTGCCCTGTCTCTTTCAATGTTTGGCAGAGAAGATGAATTTTTACACGCCAACTTATGAATGGAATTTGGATAACTGGGTCCAGGCATTTGGACCGATGCGCCAAGATCGCACGGCTTCAAATGTCTGCAAAGTGAGCGCTGAGCAAATGAAGACACGCGACAAGTGCGAATGGATGTACGAGGAGTACAACTGTTTGGAACGCTTGAACTACAACACAGACGGTTCTTATCCATTGGAAAGCACAACATTGAGTAGCGTTATCGCCCGTAAAACGGCAGCGTCAGTCGAAGACAAGACAAAAGCCTCCTAA

>BdorOBP83g

ATGAATACCCAGCTGATCTTGTTGCTGGCCTGTGTGGCATTGGTTGCAGGCAAATTCCAGATTCGTACAGCTCAAGATGCGCTTGACGCACACGAGGCGTGCCATGAGGAGTATCGCGTACCGGAAGACATTTATCAGAAGTTTTTGAACTACGAGTTTCCAGCGCATAAACGTACCAACTGTTATGTGAAATGCTTTGTGGAGCGAATGGGACTCTTCACCGAGGAGAAGGGCTTCGATGAGAAAGCGATTATAGCGCAATTTACTGCGAAGAGCTCAAAGAATTTGGCCAAGGTATCACACGGACTGGAGAAGTGTCTTGATCACAACGAACACGATTCGGACACTTGCACTTGGGCTAATCGCGTATTCTCCTGCTGGATATCCGTGAACCGACCGATTGTGCGCCGGACCTACATTGAAAACTAG

>BdorOBP84a-1

ATGATCAATCATCGACTGCTCAGTCTTGCTCTCTCACTGGTCCTACTTGGTTTTCTTGCTGGAACTAGGGCACATCCTGAAACAGATGCAACGGATAACAAATTGGATAAGCAACAGTCCATGGAGCCGACCACACCGACTGCAGGTGCTGCGAACGAGACGGGCTTTGACTTTGAGGAAGTAGTGCGCACTTGTAATGCCAGTTACACAATACCGTTAGAATACATACAGCAATTCAATGAAACCGCAGAATTGCCGAACATAACGGACAAAACGGGCATGTGTTTCCTGAAATGCTACATGGAGAAGACGGGCCTTTTGCGAGATTGGCAGCTGAATCCAACACTGATCCGGCAAACTATGTGGCCAGCCACTGGCGACTCCTTACCCGTCTGCCAGAATGAAGGCTCTCGTGAGACCTGTCCTTGCAAGCGCACCTATGCTATCGCTAAGTGCCTGACGTTACGCGCTCTCGTGGATGCCCGCAATAAGCCGTTGGTTTGA

>BdorOBP84a-2

ATGAGTAACGGTAATGTATTTGTGATGTTACCGTTAACGATAATACTGCTTTATTGTGGCATTATGGTGTCGGCACAGGATCGCGCCAAGGATAATGGTGACATTTTTGTGCAGCACAAGGAGCAGCGAGAATGTGTTGCGCCAATTATGGTGCAAGCCAATGGCAGCGTCAGCAGTGAGGGCATGGATGTGGCGCATATATGCAATAATAGTTTTTCCATACCAAGCGATTATATTGTGCAATTCAATAGGAATGGCGACCTGCCAGAAATAGTTGACAAAACCGGCATGTGTTTCATACGCTGCTACTTTGAAAAGGCAGGGTTGATTAAAAACTGGCAATTAAATAAGGATTTGATTATGCAAACTATGTGGCCGATAAAAGCTGATTCAATTGCGATTTGTGAACCAGAAGCAAAACAAGAAATGAACGCTTGTGTACGTAGTTATGCCATCGCAAAATGTTTGATGAAACGTGGCTTTCAAGATACCTGCAATGATACTGTTGCCTAA

>BdorOBP99a

ATGAAATTCTGCTTAGCTCTGCTGTCGCTGCTGATGGTTGTGGTCTTCGCCGTCGCAGATCATGCCAGTCACTCCGATTATGTGGTCAAAACCAATGAGGATTTGATACGCTATCGCGATGAATGTGTCTCGAAGTTGAGTATTCCCTCGGATCTAGTGGATAAATACAAGGAGTGGAGCTTTCCAGATGATGAGAAAACTCACTGTTATTTGAAGTGCGTTTTGGAGAAGTTCGAATTGTTCGATGCGGCAAAGGGCTTCGATGTGCACAAGATACATCACCAATTAGTGGGCGCTAATGCCGATCACTCGGACGCTACGCACGGAGCTATTGAGAATTGCGCAAAGAAAGCCGCTGGCGATGACGCATGCGTTCGTGCATATAATGGCTTCACCTGTTTCCTTAAGAACAACGCACAATTGGTGCAAGCTGGCGTCGAAAAAAGCAGCAAATAG

>BdorOBP99c-1

ATGAAATTCTTCATTGTGATCCTTGCAGTCGTTGCATTGGCATACGCCGACGAAGAATGGGTGCCGAAAAATGTTGCCCAAATCAAGGCTATCCGTCAGGAATGCATCAAGGATTTCCCACTCAGCGAGGAATACATTCAAAAAATGAAGAACTTCGAATATCCCGATGAAGAGCCCGTACGCAAGTACTTGCTTTGTACCGCTAAGAAATTGGGTGTCTTCTGCGAACATGAGGGTTATCATGCCGATCGTGTTGCCAAGCAATTCAAAATGGATTTGGATGAAGCTGAGGTCATTGCTATCGCTGAGGGTTGCGCAGACAAGAACGTCGAGGGTAGCAGCGCTGATGTGTGGGCCTACCGTGGTCACAAGTGTGTGATGGCCAGCAAGATCGGTGAACGTGTGAAGGCTTACATCCAAAAGAGCGTAGAAGAAGCCAAGAAACATTAA

>BdorOBP99c-2

ATGAAATTCTTCCTTGTGATCCTTGCAGTTGTTACACAGACTTACGCTGAAGATGAATGGAGACCGAAAAATATGGCAGAGCTTAATGCCATCCGCCAAGAATGTTTTAAGGAATACCCACTCAGCGAGGAACAACTCCAAAAAATCAAGAACTTTGAATATACTGATGAAGAGCCCGCACGTAAGATCTTGCTTTGTACCGTAAAGAAATTGGGTGTCTTTTGCGAGCGTGAAGGCTATAATGCCGATCGTGTTGCAAAGCAATTTAAAATGGATTTGGATGAGGCTGAGGCCCTTGCCATTGTTGAGGGTTGCTTGGATAAAAACTTAGAAGGCAGCAGTGCCGATGTATGGGCCTATCGTGGTCACGAGTGTGTGGTGGCCAGCAAGATCGGTGATCGTGTCAAGGCTTACTTTCTTAAATCAAAAAAATAA

>BdorOBP99c-3

ATGAAATTCTTCATTGTGATCCTTGCAGTCATTGCACTTGTATACGCTAAAGATGAATGGGTGCCGAAAACTGAAGCAGAACTTAAAGTTATCGTCAAGGAATGCCTCAAAGATTTCCCACTTAACAATGAACAACTCCAGAAATACACAACCTTCCAACAACCCGATGAAGAGCCCATTCGAAAGTACATGCTTTGTACCGCTAAGAGAGTGGGCTTCTTCAGCGAACATGAGGGGTGTCATGTCGATCGTGTCGCCAAGCAATTCAAATTGGATTTGGATGAAGCTGAGGTTGCTGCCATCACTGAAGGTTGCGCTGACAAGAACGCCGAGGGTAGCAGCGTTGACGTGTGGGCCTATCGTGGTCACAAGTGTGCGATGGCCAGCAAGATCGGTGAACGTTTGAGGGTTTATATTCAAAATCTCAAAAAAGAAGCAAAAAAACATTAA

>BdorOBP99c-4

ATGAAATACTTTATGtTTATTGTGATCCTTGCAGTGGTTGCTCTGGTACAAGCCGATGACTGGAGTCCAAAGACCGTTGATGATATTAAAAAAATTCGCGAGGAATGTATGAAACAAGTGCCATCTAGTGATGAAGAATTCCAAAAGAGGAAAGAAAATGACTATCCCGACGTAGAGTCCGTACGGAAATATGCGCTTTGCAATAGCAAAGGATGGGGCCTcTACAAAGAGGGTAAAGGTTTTTATCCCGACCGTGTTGCCGAGCAATTCAAAGACGATATGCCGGAAGACGAGATCAAAGCCATTGTTAATGATTGTGATGAAAAGACCAAAGAGGAGACCGATGATGAGAGATGCTACCATTTGTTGAAATGTGTTATGTCAACCAAACTCGGTGATCATATTAAGGACCTCGTAAAGCGATTAGAGTAA

>BdorCSP1

ATGAAAGCGGCCATAATATTTTTGATAGTTGTTGCGGTTCAGTATGCAGCAGCGCAAAAGCAGTACACAAATAAATTTGATAACGTCGATGTGGATGGTGTTTTGTCAAATAATCGTATTCTAACTAACTATATTAAATGTCTTATGGATAAAGGTCCTTGCACTCCAGAAGGTCGCGAGTTAAAAAAATTACTACCTGATGCCCTACAGACGGATTGCTCAAAATGTACAGATACCCAAAGAAAAAATTCACAAAAGGTAATAAATTTCCTTCGTGTGAACCGTCCAGGCGAGTGGAAATTGCTACTAGACAAATACGACTCCAGAGGAGTTTACAGATCTAAGTATGAGAAACAAGGTTAA

>BdorCSP2

ATGAAACTCTTTATATTGGCCGGCGTTTTAGCTTTAGCTTATTTCACCACTGCCGAAGATAAATACACAACTAAATATGACAATATTGACGTTGATGAAATATTAAAGTCAGATCGTTTATTCAATAATTATTTTAAATGTTTGATTGAAACGGGCAAGTGTACACCAGAGGGACGAGAACTAAAGAAGACGCTTCCAGATGCACTGAAAACTGAATGTAGCAAATGTAGTGAAAAACAGAAACAAAATACCGACAAAGTTATACGTTATGTCATTGATAACAAACCCGATGAATGGAAACAACTGCAAGCTAAGTATGACCCTGAGGGTATATATGCAGCCAAATATAAGAAAGAAGCGGAGAAACAAGGCATCACAATTTAA

>BdorCSP3

ATGTTGCGCTTCATAGCCGCTTCGGTGCTGATTTGCGTCGTTTACCATGTAGCAACCACAAGTGCTGCACCGCATCCACCGACCACAGCAGCGCCGTTGGTGGCTAATCAGGCCGCCTACGACACGAAATTCGATAACATCGATCTGGACGAGGTTTTGAACCAAGAGCGTCTGTTGCGCAACTACATTAAATGCCTGGAGAACACAGGTCCCTGCACGCCCGATTCCAAAATGTTGAAAGAGATACTCCCCGACGCTATTTCAACCGATTGCGCCAAATGCTCGGAGAAGCAGAGGCTTGGCTCAGCGAAGGTGACGCACTTTTTGATCGACAATCGTCCGGAGGATTGGGCACGTTTGGAGCAGATATACGATCCGCAAGGCAATTACCGTTTGAACTATTTGGCGGCGAAGGACAAAGGCGATGGCGTGGAGAAAACTACGGAAACGGTTACTAAGACACAAGCCTGA

>BdorCSP4

ATGAAAATTACTAACGTGACAAAAGATATTGGATTTAAATGTATATTTTTGCTATTGGTTATATGTACTTGTGAAACTGACTCAGATGACAAAAATATAAACAAACTTCTTAATAATCAAGTTATTGTGAGCAGACAAATCATGTGTGTTTTGGAAAAAAGCCCTTGTGACCAACTCGGTAGGCAGTTGAAAGCTGCTCTCCCGGAAGTTATTTTACGAAACTGTCGTAATTGTTCGCCACAACAAGCTCAAAATGCTCAAAAATTAACAAATTTTTTACAAGCTAGATATCCAGATGTTTGGGCAATGCTTCTAAAGAAATATCAAAATATTTAA

>BdorORCO

ATGCAGCCCAGCAAATATGTGGGGCTTGTGGCCGACTTGATGCCCAATATTCGTCTTATGAAATACTCGGGCTTATTTATGCACAATTTTACCGGCGGTTCAGGGCTCTTCAAGAAGATTTACTCGTCCGTACACTTGGTGCTGGTTCTGGTGCAATTTCTATTGATACTGGTGAATTTGGCGTTGAATGCGGAGGAGGTGAATGAGTTGTCCGGCAACACGATTACGGTGCTCTTCTTCACACATAGCATAACGAAATTCATCTATCTGGCCGTGAGTCAGAAGAACTTCTACAGAACATTGAATATCTGGAATCAGGTAAACTCGCATCCATTGTTCGCCGAGTCGGACGCACGCTACCATGCGATCGCCCTCGCCAAGATGCGCAAACTATTCACCTTGGTGATGCTGACGACCGTCGCCTCGGCTGTGGCTTGGACCACCATCACCTTCTTTGGCGAGAGTGTGAAGTTTGCTTTTGAGAAGGAGACCAATTCGACCATCACCGTGGAAATCCCGCGTTTGCCCATCAAGTCGTTCTATCCATGGAATGCCGGCGCGGGCATGTTTTATATTATAAGCTTCGCTTTTCAGTGCTACTATCTGCTTTTCTCCATGGTGCATGCGAACTTGTGCGATGTACTTTTTTGCTCATGGCTGATTTTCGCCTGCGAACAGCTGCAACATCTGAAAGGCATTATGAAGCCATTAATGGAGCTGTCAGCCTCGCTGGACACCTATCGGCCAAATTCGGCGGCCCTCTTTCGTTCATTATCCGCCAACTCGAAGTCGGAATTAATCAACAATGAGGAGAAAGAACCCACTGATCTGGACATCAGCGGCGTCTATAGCTCGAAAGCCGATTGGGGCGCACAATTTCGTGCGCCATCGACTCTGCAAACCTTCAATGGCATGAACGGTACAAATCCGAATGGTTTGACCAGAAAACAGGAAATGATGGTGCGCAGCGCTATAAAATACTGGGTCGAGCGGCATAAGCACGTGGTTCGATTAGTGGCAGCAATTGGTGACACTTACGGCGGCGCTTTGTTGTTGCACATGTTGACATCCACCATTATGCTGACGTTGTTGGCATATCAGGCCACCAAAATCACCGGCGTGAATGCGTACGCTTTCACAACCATCGGCTACTTGGGCTATGCCTTGGCGCAGGTATTTCACTTCTGCATATTTGGCAACCGGCTCATTGAAGAGAGCTCATCTGTCATGGAGGCAGCCTATTCGTGCCACTGGTATGATGGCTCCGAGGAGGCCAAGACTTTTGTTCAGATCGTTTGCCAGCAGTGCCAAAAGGCGATGTCCATATCGGGAGCGAAGTTCTTCACTGTCTCGCTGGACTTGTTTGCATCGGTTCTTGGCGCTGTTGTCACCTACTTCATGGTGTTGGTGCAATTGAAGTAG

>BdorOR7a-1

ATGTTTGAACTAATAACCGGACGAGGTATACGAAATGCTGCCTCAAAGGATGCATTCATCTATTTCTTCAAGGGTTGTACTATTGTGGGGATAAGTCCACCAAAGTATGCTGGTCTGTTATATTATATGTGGTCGTTTCTGGTTAATACAATCTGTATAGTAATTGGACCAATTACCGCCACAGTTGGCTTTGTTATAAAATATATGCAAAACATCATTTCAACTGTACAATTTCTGAGCGGTTTACAGGCCAGTTTAAATCTGATTGGCTTACCCGTGAAGTGTTTAACGGTGACAAGCGCCTTGAACCGACTGCGCGGCATGGAACCAACTTTGGCGGCGTTGGATGCACGTTACACCAGACCCGAAGATATGACTTTAATACGGAAAGCTGCAGTCATGGGCAATAGATTGGTTTTCTTTTTTGGCACCTCATATCTGATGTATATGCTGTTTACGGTCATACCGCCCTTAATCAATGGTAAAGCGCCACTTTCGGTATGGATTCCGTTTTACGATGAGCATCAGTCCACAATGCATTTCTTCGGACAAATAGTTTATGACTTGTTCTTGATGGGTTTCGTATTATTTCATCAAGTCCTCTACGATTCATATGGATCGGTATATATTTATGTTATAAGTACGCATTTACAGTTATTAGTGCGTCGGGTCGGTCGTTTGGGCACAGATGCTACGAAGAGTAAGGATGACAACTTGAATGAGCTGGTGGATTGCGTAGTTACGCATCAACAGATACTAGAATTGCTGGCTACAATTGAACCGATTATTTCCAAAACAATTTTTACACAATTCCTCATTATCTCTTCGATTTTGTGTGTGACCATGGTGAATATGTTCTTTTTCGCCGATCGCAGCACCCAAATCGCTTCGACTCTCTACTTCTTGTGTGTTCTATTGCAGACGTCGCCTTGTTGCTATTTCGCTACCGAATTGAAGGCCGACAGCGAGAAATTGCCGCTGGCCATTTTCCATTGCAATTGGCCTGAGCAGGATCGGCGTTTTCGCAAAGTGATTCTATATTTCATGCATCATGCGCAATTGTCTATTGAATTGATGGCCATGCAGTTGTTTCCCATTAATGTGGCAACAAATATTTCGCTGGCTAAATTCTCTTTTACACTTTTTACATTCATCAAGGAGATGGGCATTGGTCAGGAAGCATAA

>BdorOR7a-2

ATGGGGAAGACATTTGAGATTCGCTGCAATATGAGGCAGTCACCGCGACAAGCCACTGCTGAAATGCCAAAAATGGGACTTGCATTAGCAATACACAATGAAATCGCAACTCAGCGGGATATTAAGATCAAATCCAGTAGTTCAGCGAAAGCTTTAAGTGAAATTATTGAGGAGGAAGCTGCTGAACAGCATGTGAGTAGCCAAGATACGACGAAGTATCTCTTCAAATCTGCGTTCGGAATGGGCTTAGTCATGCCTTCGCGCTATCGGGCACTGTACATCATTTATGGATTTTTGGTCAACTTCTTTACCACTTTCTATTTTCCCATTGGATTTACCTTGATATTGTTCACCTTGCCAGATGATGTCAACGTTAGCAATCTGCTAACTTCGTTGCAGGTCACATTCGACGTATACGGCGGTTCGGCGAAAATCATCATAATGAAGTTTGTATTGGAAAAACTGCGTGCAACACAAATACTCACGCAGCGACTTGATAAGCGTTGCCGTGCATCGGATGAAGTCGAGGAGTTGCGACAAATGATACGGTTTGGTAAAAAGGTCGTTATTTTTTATTTGACCATCTTTCTGTGCTACTCGGCCAGCACATTTCTGGCCTCGGTTTCTTCGGGATATCCCCCGTATTCGTTATATTTTCCCTTTCTCAAATGGCGACGCTCCCGCACGGAGTTTGTAATTGCGTCACTCCTTGAATTCATTATAATGGACTTCGCTTGCCTGCAACAAACTGTAAACGATGGCTATCCCGTCATCTACATTAATATGTTGCGCTGCCACATGAAAATTCTACAGTTTCGTGTGGAGAAACTGGGTACTAATCCAACACTAACGCAGGTAGAACATCTCAGTGAATTGAAATTGTGCATCAAGGACCATCAATTGTTGATTGAGCTCTATGACACTATAGCGCCCATCATCTCGATAACGCTTTTCATACAATTCGCGCTCTCGGCCGTGTGTATTGGGACAGCACTGATCAACATTGTCATCTTTGCGAATGAGTTCCAAACACAAGTGGCCTGCAGCTTCTTCATACTGGCCGTGCTGATAGAGATTTACCCAGCTTGCTACTTCTCCCAGTGCCTTATCAATGAGAGTGACAAATTGGCGGACGTGATTTTCCACTCAAATTGGATTGAGCAAAGCCCAGAGTATCGCAAACTAATTATTTTCTTCTTGCAACGCTCACAACGTCCCATGTTTCTGACCGCTGGGAAACTGTTTCCCGTCACGCTAAGCAGTTTTATTGCAAAGTTCTCTTTCTCCCTTTACACTTTCATCGAAAAAATGAATTTGAAGGAACGATTCGGGATCGAGTGA

>BdorOR7a-3

ATGCGTAAAATCGCCGATTTATTTTATGGCCGCGGCAAGGATGACTTCGAAACGACTGAATCATTTGTACTTTTGTTTCGTGGTTGGGCTGCAGTTGGTTTTTTACCAAAAATACCGAAGCGTATTGTTGACATAATTCATCAGATTATTTGCTGGTGCTCCATATTAACCTGTCCTGTTTGGTATTTTGCGGGATTAATAGATATGATGGACGATTTGCCCATAACACTCCTATTGTCTAACTTGGGAGTCGCTATTAATTGTATCGCATTACCATTGAAAGCGATTTACATTAAAGTCAATATGAATCATTTGCATGACATCAATTTGCTGTTCAAACGATTGGATGAACGTTATCAGACGCCAGAAGAAAATATACAAATCAGAGAGTCAGTTAAAATTAGCACACGGATCTTTGCAGCATGTTGCACTCTGTATTGGTTCTTCGGTATTTCAAGTGGATTGGTACCGCTTTTCGCACATGAATATCCGCATGGCAATGTATTTCCATTCATTGATTGGCTGCCAGAAGGGAATTTTCAATATTGGCTTCACTCCATTGTGGAGATGGTCAATTTACAATATTTGCTGCATTTACAAAGTATAAATGATTCATTTCCGGCAGTTTATATACGTAATATTCGTACGCATATCAGACTCTTGACGAATCGTGTGAGTCGTTTAGGCTTAGATCCCGATTTAAGTGATCAACAGAATTTTGAGGAATTAGTCGATTGCATCGTTTCACATCAGGAAATACTTGTGATTTCCGATACCGTTGGGCCGATCCCTTCCCTCACAACGTTCTTTCAATTCACTGTCTATGCGGCCCTTATCTGCGTCTGCATGCTTAATATGTTCATATTTGGCGATTTGAAGGTGAAGGTGAGTACGTTGATATATCTCATACCTGTCGTCTGGCAGACTGTGCCGACCTGTTATCAAGCTTCGATGCTCGAGACGGACTGTAGTAAATTACCTGAGGCAATTTTCCATTGCAATTGGCTGGCTTTGGATAAACGTTGCCACAAATTGATCATCTATTTCATGCAATGCACCCAGGAGGAGATCTGTTTTACCGCAATCAAATTGTTTCAAATTAACCTGGGAACTAACTTGTCGATTGCCAAGTTCTCGTTTACTTTGTATACCTTCATAAAGGAAATGGGTTTGGATGCACACTATAACCAGAAGTGA

>BdorOR7a-4

ATGTTCGATTTGATAAAAGGACGCGGTCGCACAGTATTCGCCTCACGTGATGCAGTCATTTATCTGTTCAACAGTTTCAGATATCTGGGCATCAATCCACCGGCCAAATATCGTTTGCCATATTTCATGTACTCGGCCATCATAACATTCTTCGCGGTGCTCTTTTCGCCGGTGATTTTCAATGTCGGCTGGTTGCGCGATCGCAACAAGCTCTCTGTTATGGAAATCTTAACTTGCGTGCAGGCCTCCTTGAATGTCATGGCAGTGCCACTTAAGTGCATCACGCTGGCTATGGCGCAGAAGCGTTTGCGGGGCATTGAGCCAATGGTGACCGAATTGGATGAGCGCTTTCCAACGCAGGAGGATAAGGCGAAGATCAAAAAGTGTGCCGTAACGGGTAATCGACTGGTATTCGGTTTTGCTGTTTCATATTTTATGTATGAAACTTTGACTGTGGTCTCCGCCTTGGTGGGTGGCCATGCACCGCTCTCGCTTTGGATACCCAATGTCGATTGGCATCGCTCCACTTGGGAGTACTGGCTGCAAGTTAGCTTCGATGCAGCTGTGCTTTTCTTTTTGTTATATCATCAAGTTTTGAACGACTCATATCCGGCCGTTTATATTTACATAATACGCACACAGGTGCAACTACTGACAAGTCGTGTGGAAGAGTTGGGTTATGATGAACAAAAGAGTGTCGATGAGAATTACCAAGAGCTCTTAGAGTGCATCGTAATACATCAGAAAATATTGAAAATTGTCAAAATTGTTGAGAGTGTCGTCTCAATAACGGTATTCACACAGTTTCTAGTCGCAGCAGCTATACTTGGCGTTACCATGATTAACATTTTCATATTCGCTGATCTCACGACGAAAATCGCTTCGGTCACTTATTTCTTCTGCGTATTGCTCCAGACTTCGCCGACATGCTATCACGCCTCATATTTGCTGGACGATTGTGATCAGTTGCGCATTGCTATTTTCCAATGTAATTGGATTGCGCAAAATAAACGCTTCAATAATTTGCTGATCTATTTTTTGCATCGCTCGCAGGATTTTATGCCATTTTTCGCTCTAAAATTGGTGCCAATTAATTTGGCGACCAATTTATCGATCGCTAAATTTTCGTTTACGCTCTTCACTTTTATACAAGAGATGGGTCTCGGTGAGAATCTTAAGGGTTAG

>BdorOR7a-5

ATGCGTAAAATCGCCGATTTATTTTATGGTCGCGGCAAGCATGACTACGAGACAACTGAATCATTTGTACTCTTGTCGCGTAGTTTTGCTGCAATCGGTTTTGCACCCAAGAGACCGAAACGTATTGTTGACGTAATTCATCAGCTTATTTGTTGGAGTTGCATTTTCAGCTGCCCTTATCTGTTTGTTTCTGGAGTGGTAAAAACTATGCACTCTTTACCGATAACAATCGTACTTGCGCATTTAGGAGTCGCTATTAATAGTATTGTATTCCCTTTGAAAGCTGTTTATATTAAAGCCAATATTGACCGTGTGGATGACATCGGAAAGATTTTCAACGCTTTGGATAAACGTTATCAGCGACCGCAAGATCAGATGCAAATCAGAGACTCAGTCAAAACTTGCACACGCATATTtGtTGTTTTTTGCATTGCGTATTGGTTGTTTGGCATATCAAGTTGGGTGGTAGCGATTTGCATACATGAATATCCGCATGGCAATAACTTACCATTCATTGATTGGCTGCCAGAATCAAATTTGAGATTTTGGCTTCACTTCATCTTTGAGGTAGTATTTCTGCACCAACTGCTACAAATGAGTTTGACAATGGACTCGTTACCTGCTTTATATATACATGCTTTGCGTACACATATGAATCTCTTGACGGATCGTGTGAGTCGTTTGGGCCTTAATCCCGATTTTAGTGACCAGGAGAATTTTGAAGAATTAGTCGATTGCATCGTTTCGCATCAAGAAATACTGCAGATTTCCGATACTGTTGGCAAGATCCTTTCTCTCACCACCTTCTTTCAATTTACTGTATATGCCGCTATACTCTGCGTCTGTATGCTTAACATGTTCGTATTTGGCGATGCATCGACCAAGCTGGTCACGTTAGTTTATCTGTTACCCGTATTTGGGCAGACCACTCCCACCTGTTATCAAGCTTCGATGCTCGAGGCGGACAGCGCTAAGTTACCTCTAGCAATTTTCCATTGCAATTGGTTGGCTTTGGATAAACGTTGCCACAAATTAATCATCTATTTCATGCAACGCGCTCAGCAGGAGATCTCCTTTACCGCAATCCAATTGTTTGTGATTAATTTAAGAACCAACTTGTCGATCGCCAAGTTCTCGTTCACTTTATATACTTTCATAAATGAGATGGGCTTCGGAGAGACATTAAAGGATAGACTAGAGTAA

>BdorOR13a

ATGTTATTCAATCCGAAACCGTCGAAGGATCCAAAAAACTTCAGATTTCCCTTACAATGCATCTGGTTAAAATTGAACGGTTCGTGGCCACTGAAACCCAAAGTGACTGGTGAGTTTCAGAAATACTTGCGTTTGCTGTACAGCATTTGGGCCTGGTATGTGGTGGCCATGGTTGGCATAACCATCGGTTTTCAGAGCGCATTCCTGCTCAAATCCTTCGGCAATATAATGGTCACCACGGAGAATGGTTGCACCACATTTATGGGTGTGTTGAACTTTGTGCGTTTGCTGCATTTGCGACTGCATCAACGGGACTTTCAACAATTGTTAGCACAATTCGTAAAGGATATCTGGATTACGAGCTCCTCCCACCCCACCGTTGAGCGCGCTTGTGCCCGCAACATGCGTGTCTTCCAAGTGATTTCGGTGCTCCAGTCCAGCCTCATTACCATGTACTGTATTCTGCCCTTGGTGGAACTTTATATGCTAACGTTGAATGTGGAACCCGATGTCTTGGATAGCATGCCGAAGCCATTTCCTTATAAAATGTTATTCCCTTACGACGCAAATCATGGCTGGCGTTATGCGCTCACCTATTTATTCACCGCTTGGGCCGGTGTTTGTGTGGTGACCACACTCTTTGCTGAGGATTCGCTATTTGGCTTCTTTGTATCCTACACTTGTGGCCAGTTTCGCATACTACACACGCAAATCGATAATATTATACCTGATTCGTATGCAGCGACGCGAGCTGGGCGTGGCACAGAGGTGGTCTTCCAACGGGAATGCATTCGACGTCTGGATAAAATCGCCAACAAACATTGTGTATTATTTAATTTCGTCAGTCGCATGGAAGAGTTCTTCAGTCCAATTTTGTTGGTGAATTTCTTGATTTCGTCGGTTCTAATTTGCATGGTGGGCTTTCAATTGGTGACGGGTCAAAACATGTTCATTGGTGATTACGTAAAGTTTTTGGTTTACATTCTCTCATCGCTCTCTCAATTATTTGTCCTTTGCTGGAATGGTGACAATACAATTCAAAACTCGCTTGAGATGGCCAACCACTTGTATGCCTGCAACTGGGAGAGTAGTGTGAAAGTAGCTGCCGATGAAGAAACAAAGGAATCCTTTCCAATAGTTTCTTATTCCACCAGTGCCGCTTTTCGCAAAAACTTGCAATTCATGATTATGCGTAGTCAACGACAGACATGCATAACGGCGATGAAGTTCTCAATATTGTCGCTGAACAGCTTCTCCGGCTTAATCAGTTCGTCGATGAGCTATTTCGCACTCTTGCAAAGTTTCTATGAGAACGAAGAAAACTAA

>BdorOR35a

ATGGATTACTTTGTGCCTTTGCAGTTCGGCAATCGGCCAATAAAACTACCCATACAAGTAGCGGGTTACAAATTCAATTTCCTGTGGCCACTGAAGGAGGATGCCGGCATCTTAAGCCGCCTCGTCAATAACATATGCCTCAGTGTGAGCGTGCTCTGCTACATTGGCACCATTGTGGGCGAGTTTACCTTCATAGGTGAGAATATCGCTGACATAGCAGCCGTGGCTGAGTGCCTGTGCACCTCTTTTATGGGCGTGCAATACATAATACGAATATTTGTGCTGCTCAGTCGACAGCGCGCATTACGAAAACTACTTCGAAATTTCTATCGTGACATTTACTTTACGCCCGCTGACGATGCAGGGCTGTACAAGGAAATCAACTCCATAATGCGATTTATGAATATATTTACGCAATTCTACTATGTGCCCATGATGTTGATACTGGTGCTGTACGTGTACGATGTGGCAAGCGTGGGGTTGGCGTCACCTGACAAACCATTTATCTATCGCATGTCCTTTCGTTGGTATGACGCACAGGTGCCGCTGCAATTCATCATCACCGCCATCTATTCGGGTTGGCTGACGATCTCTTGTGTGACAATATGGACCGCTGAGGATTATACGCTCTGCTTGGTGCTCTGCCATGCCAGTTTTCGCTATAAGAAATTACGCTTGGATTTGCAGCAGCTACTGGAGATGGCACGTGCTGATCTGAAGTGCGGCGAGACACCTTGCACGAATCAAAATTTGCATATAGCCTTTCGGCGACGTCTTCGTGAGATTTTTCGACGGCAGCAACGTTTGAATGGCTTCGTCGCCGAGGTCAAGGCTCATTTCACCCACCAAATATTCTACATTATGTCCTTTGGCGTTTTGCTGCTGTGCGTTGTCTCCTTTCAATTTCAGAGCGGTCCAATCACGGTGGCGTCGAGCAAATACATTTCCTGGCTCATTTCGCAAACTGCGCAGTTTCTGTTAATTGGCTATTTCGGTCAAATGCTGATGGATGAGACCACGGAGCTGCGAAATAGCTTCTACTGTTGCCGCTGGGAGGATTTGTTGGTGCTCGGCGATCCGCATAGCAATAAATTGTTGTTGGGCGATGTACAATTTGTCATCATGAATTCGCAGGAACCCATCGTTTTCGATGGCATGAAATTTTTTCCACTCACTTACAGCACGGTGAGTGCGGCTCTTCGTTCGGCTGTGTCGTATTTTATGTTTCTGAACACTATGAATGGCGAGAATTGA

>BdorOR43a-1

ATGGTCACCGCGGTCGTAGACAACCCGATGCTCTCAGTCAATGTGAAGCTGTGGCAGTTCCTCTCCGTGCTCTTTGCACGCGATTGGCGGCGCTGTGTCGCTTTAGTGGCACCCGTCTGCCTAATGAATGCAATGCAGTTCGTTTATTTGTATCAACAATGGGGTGATTTGTCCACTTTCATATTGAATACCTTCTTTGCGGTCTCCGTTTTCAACGCCTTGCTGCGTACGTGCCTTATTATTAAGAATCGAGATAAATGTGAAGCGCTGATGGAAGAATTGGTAACATTATACGACGATATACAAGATTCGGATGATGATTATGCGAAGAGCGTGCTGGCTGCAGCCACAAAAAGCGCACGAAACATTTCCATTTTCAATTTGTCAGCGTCGTTCTCCGATTTAATTGTGGCAATGGCATATCCACTTTTTCAGCAACAGAGAGTTCATCCTTTTGGCGTTGCATTACCCGGCATCGATGTCACACGCTCTCCACTCTATGAGCTCATCTATATCGGTCAATTATCTTTCCCGTTCACTCTGTCCAGCATGTATATGCCCTATGTAAGTTCATTCGCCACTTTCTCGATGTTCGGAAAGGCAGCACTACAGATATTACAAAACAACCTCAGAAATTTATGCGATAATATGAAAAGTAAAACCGAAGAAGAGCTCTTCGAAATACTACGAAAGAATATCGCCTATCATGCGAGAATCGCTAGATATGTGAGTGACTTCAATGAATTGGTGACCTATATGGTACTCATCGAATTTCTGCTCTTCAGTTGCGTTATTTGCTCGCTGCTCTTTTGCATCAATATTACAACCTCTACGGCAGAGAAGATTTCCATTGTCATGTACATTGGCACAATGCTGTATGTGCTCTTCACCTATTACTGGCAAGCCAATGGAGTTTTAGAAATGAGCCACCTCGTCTCAGATGCGGCTTACGAAATGCAATGGTACGATTGTAGTCCGCGTTTCAAGAGAACTCTACTCATATTTATTGCACGCACGCAAAATCCTTTACAGATCCGCGTTGGTCAGATGCACCCAATGACAATGGAAGTATTTCAATCATTGCTTAATAACGCGTACTCCTATTTTACGCTTTTGCATAATCTTTATAATGATTAA

>BdorOR43a-2

ATGACCAGCTACGAAAATCTACCGTTATATGCAGTAAATGTGAAAGTTTTTGTTAAAGTGGGACTAATCAACTCAACTGACTGGACGAAGGGACTTCTATTTTTCTTAATTCTAATAGTAGCATATGTAGGGCAAATCATAAATTTGTGTAAGTCATGGAATGAGGACATTGGCGAGACAACCATGAATTTTCACTGTTTTCTCTTTGTGACACATTGTTTAATTCGTTTATGGATCGTCGTGAAGAAGAAGAATAAATTTGAGCGATTCTTTCAATGTGTCGAGCAGTGGCACAGAGAAATCGAGCGAAACGACGATCCACAAATGGTCGGTATACTACAGGAAATCACCAAACGAACTCAACTGCTAAGCAAAATGACCATCTATGTAGCCGCTGGCGGAACACTAGCCGCTATCTTCTATCCATTATCCTTTGATGGACGCAAACATATGATAACCATGCAATTCCCACACTTCGATGTCCTACAAACACCATTCTATGAAATATTTTTTCTCATCGGAGTGACGTGGTTAACGCCGGCCTTTTTAGTTATATCTCTCCCGTTTACGAATATTTTCCTCATATCCCTCATGTTCGGTGAACTGGTCTTAAAGGATTTGTGTGTGAAGCTAAGAAATATACGTAGTGAGAATGAGGAAACCATGCTACAGGAGTTCAAGGAATGCATTGCGTATCACCAAAAAATTATCGATTTGTGTGATGATCTCCAAGATTTGCTTTCAATAGATGGATTCTTTCACCTTGCGCTCTTTGGTATGATGCTATGCATGCTGCTATTCTTTCTTTCAATGATACATGATCTCCGACTTATTCTAGCGGCACTGTCATTTGTCAGTTTCACTACTTATATGCTCTTCATTACCTATTATTATGCGAACAAACTAGTAACTGAGAGCCTTGAAGTGGCCAACGCCGCCTACGACACACCTTGGTATAGAGGAAATTTGGAAATGCGTAAATGTGTCATTACTATGATCGCAAGATGTCAGAAACCTCTGCAAATGACGGCCGGTGGAATATATCCGATGACTATGGAGACTTTCCAAGCCATTTTACGTGTTTCATATTCATATTTTTCCCTACTACAAGGTCTCAATCAACAGTAA

>BdorOR43b

ATGGGTTATCTGCACACTTTGGAAACGGAGCCTATAACTATTCAACTTGGCATTTTGCAAGCAATATTCAATATTCTAGGACTGCCAATGAAGGCGATTGTTATTACAATTTTGCTAACGCATTTACGAAGTGCCGAGCTGATCTTTTCACGTTTGGATGCACGCTACCAGAGCATCGCGAGTCGGGAGCAGATAAAAAATTGTGTTATCATCAGCACACGCCTACTCAGCAGCGTCATATTTGTGTTTCATTTTTATGGCAGCGCCACATATCTCCAAGCGCTGCTCACAAATGGTTATCCACTGAATACATGGTTGCCATTCACCGACTATATCCCTCAGCCAACCATAAGATATTGGGCGCATTTTATTTTCGAGGTTTTTCACTTGATTTTCCTATTGACGGTACAAGCCGCAATGGATGCGTTCCCGGCAGTTTATATACGAAATTTGCGCACACACTTAAATCTGTTGACGGAACGCGTTAGTCATTTGGGCGAGAATGCAGAGCTTACTGAGGAAGAGCACTTTGAGGAATTGGTGGACTGCATTGTTACCCATCAGGAACTGTTGGAGGCGAAAAATATTGTTGAATCCGTCTGCTCGATAACATTATTTATTCAATTTGTGATTGTTGCCGTAGCACTCTGCGTTTCGATGCTAAATTTTTTCGTATTTGCCGATCGCCAACAGCAGGTGGTGACGGTAACCTACTATTTAGGCGTCATGTTGCAGATCATGCCCACATGTTATCAAGCGTCAATGATAGAAGCTGACAGTGCCAAGTTGCCCGACGCCATTTTCCATTGCAATTGGTTGGCCATGGACAAGCGTTGCCGCAAGCTCATCATCTATTTCATTCATCGTGCACAGGAGGACATCACCTTTGTTGCACTCAAACTCTTCAACATCAATCTGACTACTAACCTGTCGATTGTCAAATTTGCGTTTTCATTATACACATGGATGAGCAACATGGGCTTTGGGCAGAATTTAAAGGATCTATTGGAATGA

>BdorOR45a

ATGACGACGAGAAGTGCTCGCGTGATCAAAATATATTTTTTGACTCTACAAATCATCGCCTTGGCCACGATTCTAATACCGATCGCAGTTTGCAGTTGGCAACACATACAGGAGATTGTCGAGGTGACCAATGCAATGGCGCCCTTTATGCAGGCCACCATATCGCTTTGGAAGATTTGGCGTGTCATTTATAGACGTAAGGAAATGGCGCAAATGGCTGAGAATATTTATCTGATATCGACCAGAGCCTCTGCGAAGGAACTAACTCACTTGATACAGGAGAATAATCGTGAGCGTCTAATGAACACCGCTTATTACTACTCGGTATTGAATACCGGCGTGCTCGCGCTTGCGGCCCCCGTTTTGGTGAGCTTCATACAATATCTGCGTCTCGGCGAATTCAGTTACATTGTGGTGCTCAAGGCCACCTACCCGATTGATTATGCGCATCCGCTAAATTATTTTCTGATATGGCTGTGGACAGCGGTTGCCATCTATGGCGTCATTTATGGCTCAGTTTCCGTGGATAGTCTTTACTCTTGGTATATACACAACTTGGTGGGAAACTTTAAGATACTCCAGTCGAAATTGGTCACAGCCGAGTCGGCCAGCGAGCTGAGCGAACGTCGCGAGCTAATTTACTATTGTATTGCGTATCATCAGCGCATTATTGCCATGACCGAGCAATTGAATATTATTTATCAGCCAATTGTGTTTGTGCAATTCTCACTAAACGCTTTGCAGATCTGTTTTCTAGCTTATCAGATTGGCAGCGGTGTTGTGGACACGGTGGATTTACCATTTCTATTCTTGTTCATGATTTCGGTGGGTATACAACTGATGATCTATTGCTATGGTGGTCAGCATTTGCAGAATGAAAGCGTGAATGTTTCGAAATCCATTTATCAAACTATAAACTCGTCGTCCTGGCCGAACGAACTACGCAAAGTGCTGTTAATCTCGATGATGCGCGCACAGAAACCCAGCAAACTGACTGGCATATTTTTCGATGTTGATTTGCCGCTCTTTTTATGGGTTTGGCGCACTGCTGGCTCTTATGTGACACTTTTGCGCAGCGTGGACCAGAAAACTATGTGA

>BdorOR49b-1

ATGTTCGACGATCTGCAATTGATCCACATGAGTGTGCGAATTTTACGTTTTTGGTCACTGATCTATGAACACACCTGGCGACGTTATGTTTGCCTGTCGATGACGACCTTCTTGGTTTTCACACAGCTGTACTATATGTTCCGCACCAGCGAAGGTATCGACTCCATCATAAGGAACTCTTACATGTTGGTTCTATGGTTCAACACCATACTTCGGGCGTACCTATTGCTGTATGATCGGGAAAAGTACGAAAAGCTGTTGAGCGATTTGGAAACATTTTACTATGATTTGAAGCGATCAAAGGATTCTTATATACAGGACCTACTGGTGGAAGTCAACACGACAGGCAAATATATGGCACGAGGTAATCTCTTCCTTGGGCTACTCACCTGCTTTGGCTTTGGCTTCTATCCACTTTTTGCCACGGAAAGAGTTTTGCCCTTTGGAAGCATGATCCCGGGTGTTGAGGAATACAAAAGCCCGTTTTACGAGTTCTGGTATATCTATCAAATGGTAATCACGCCCATGGGCTGTTGCATGTACATTCCCTATACAAGTTTGATAGTGGCTTTCATCATGTTTGGCATTGTGATGTGCAAGGCACTGCAGTTTCGGCTGAAGACGTTGCACCGCGTGAGGCACATTGAATCCCTCATTCATAAAAACGTCCGGGAATGCATACGCTATCAACTGTCCATTATTGATTACATTGCGCGCGTAAATGCTTTGACGACTTACATATTTCTCTTGGAGTTCCTAGCCTTCGGCACACTGCTATGCGCCTTACTGTTCCTGTTAATCATTGTTGACTCCTCGGCTCAGGCGATTATAGTTTGCGCTTATATTGCCATGATATTTGCACAGATCTTATCGTTGTATTGGTATGCGAACGAGCTGCGTGAGCAGAATCTCGCTATCGCAGCCGCCGCCTACGACACCGAATGGTTCACATTTCCAATACcGGTGCAAAAATATATTCTTTTGATGATCTTGCGCGCACAAAAGCCACCAGCGATAATGGTGGGCAATACTCAACCGATTTCGCTGGAGCTGTTCCAGTCCTTGCTGAATGCCTcttACACTTACTTCACTTTGCTAAAGCGAGTTTACACTTAA

>BdorOR49b-2

ATGACCATAGGTCGCGCCTTTATTGTGATGTGCAAGTCAAAGAAGTTCTTGAACTTTTTCGAAAGTGTTGATGAGTGGTACCAAGAGCTGCATGTGCGTTTTGCAGGCGGATCAAGCCTTTTTTTATTCAGCTACTACTTACTTACTTTTCTACACTTTCAGCGCGAAGGTGATGACGTAACATTGAAGAAAGCACACGAATATACTAAAAAGATGAAAAAGACTTCAAAAACAGTATTAATCCTTACGGGTATTACAATATTTTACGTTATGTTCGTACAGTTATTATCAACGGCTGGTGTTGGATATAAAAAACTAATCCTTGATGTCGCGTTTCCGGGTGTCGATTTTTATGAAAGTCCTTTATGGGAGATGATGTCCATTCTGCAAGGCTTATGGACTGCGCCTATTGTAATCGTTTCGTATGTCTCATATTTATGTTTGACTTTAATAGCCATTGCGTTTGGCATATTCTTGATGAAAAATCTACAGAGCAAACTGGAAGGTATGAACGAAATGACCGACGAGGAAGCATTGAAATGTATTAAGAAATGCGTTAAGGATCATGTTATGATTATAAAGTATCACAGAGATTTGGAAGTATTATTTTCGGTTAATAGCTTTGCCGATGTTTGCATTTTCGCTGTAATACCTTGTGTCATAATTATTATTTCAACCATGGACCATGATATGTCAATGTTGATTGGAGACATTCAGCTGTCTATTATGGTCATGATTTCGACCTTCCTTGTCTTCTGGGTGGGTAATAATTTCTGTTACGAGAATGAAAATATAGCTAAAGCGGCCTATAACTGTAATTGGGAAAACCGTAATAAGGAATTTCGTAAACACATTCCACTAATTATCATTACTAGTCAGAGGCCACTGCAGCTCACTGCTGGTGGTTTAAAGCCGATTAATATGGAGTTTTTCCTAACAATAGTGCGCTGCACTTATTCGTTCTTCACCGTTCTATTTACAATGACGACCGAAGGCGATTCTTAA

>BdorOR59a

ATGTCGCCATCACCATTGTCGTTACCGCAACAGGCGTTAGCCGCAGTTGATACCCGTTCATTTTTCAAACTTCATTGGACGTGCTTCAAGGTGCTCGGTATTAACGCGTCCACCTCCAGCGCCTATTATCTTGGCTATTCGTTGCTGCTGCAGGTGCTCGTTACCCTCTGCTATCCACTGCATCTCGCCTTGGTACTCTTCGATAGCGCTGATGCCTCGAAGAATATACAGAACCTCGCCATATGTGTTATCTGTGTGGTCTGCAGCGTGAAATTTGCTATCTACGCCGCGAGAATGTCGCGCATACGTGTGTTGGAGTCCATTATTGCTACGTTGGATGCACGTGCCCAGAGTCCGTGTGAGCGCCGTTATTTCGTAGAGATACGTAAAGAAATTCGTCGCATTACCCTCGGCTTCCTTAGTATTTATGCTGCTGTGGCCGTGACGGCAGAGCTGATGTTTCTCTTACGTAATGAGCATAATTTGATCTATCCGGGTTGGTTCCCGTTCGACTGGCGTGCAACTGATTTGAAGTTCTATGCAGCGAATTTTTACCAAATTGTTGGCGTCACTTATCAGTTGCTGCAAAATTTCATTAACAATTGCTTGCCAACTATAGCTTTGGCGCTGCTGTCGGCGCATATCAAACTGCTGGGTATTAGAGTTTCGCAAATTGGCTACGCTGGGGAGAGTCCCGAAGCCAATGAAGAGGAGCTGTTGTGTTGCATCAAAGACCAAGAGCAGCTATATAACATGCTCAGTGTCATTCAAAATATCATCTCGTTGCCGATATTTCTGCAGTTCACCGTTACAGCTGTCAACATATGCTTACCTTTAGCCGCTTTACTGTGCTATGTAGATGCACCGTTCGATCGCTTGTTCTTTGTGGTATATTTATTCGCGGTGCCGTTAGAAATATTTCCCATCTGTTACTATGGCACAACATTTCAATTACTTTTCGATAAGCTGCACGTTGAGATGTTCTTCAGCAACTGGGTGGAGCAAACACACAAATATCGCAAGCATATGATACTCATCTGTGAGCGTTCTTTGAAGAATCAAACAGCCACGGCCGGTGTTATTATACGCATTCATCTGGATACTTTCGTCTCGACCTGTAAGACAGCTTACTCCCTGTTGGCTGTCATCATGAAGATGAATGAATAA

>BdorOR63a-1

ATGTACAGCATAAGTGAAATAAAGGAGTTGAGAACTCGTAATCATTGGCGAATAAGAGAATTGAAGAGAATTTCATATATAATCGGTATTAATCTGAATGCTCAAACGAAGTGCAAAAGATGGTGGCGAATCGTCAATATTCTGTTCATAATTGCAAGTTGTATTGCTCTATATCCGCATTGGTTAATGATAAAACAAGCAGAAGGTGATATTCCATTGATAGCTGAGACTTCTACCACAGCGCTGCAAACGACGACCGGCCTAATTAAAATGGCTTACATGCTGTTCACTCAGCATAGATTTCATAGACTGCTTCGGAAAGCTGAAACACATGAACTGCTGCAAGGAATCGAAATCTTTCAAACAGATATGCCAATCAAAACCTCTCTGAAGAAGGAGATTAACGCAGTGATGGAAATCAATTGGAAACAGGCGAGGGGACAGCTTCTGTTTACTTTAGGAACCTGCATCTGCATTATGTCCAACTATTTCtTTTATGCATTCTTCAAAAACTTATATAATCAtTTGCAAGGCACGCCAAATTATGTATATATTCTGCCTTTCACTGGCTACCCGATGTTTCTGCACAAAGGAATGGCCTCTCCGTATTACGCAATGGACATGTTTTTCGGTGCTTGCTCGCTTCTGGTCGCCGGCATGAGTGCTATTAGCTTCCAGGGTTGCTTTTTGGTGCTTTGTAAGCATTCCTGTGGACTGGTCCAGGTGTTATGTCTACTGTTGAAGAGATCCACGTCAAGTCTGGTTCCAAAACCGCAACGTGTGGAATATTTGCGCTACTGCATAGTACAACATCAACGCACTCTTGAGTTTATCAACGAGGTCAATCAGCCTTTCAGACATATTTGTCTTTCCCAATTTCTTCATAGTCTGGCAATTTACGGCTTTGTACTCTTCGAGATGAACTTCGGCTTGGAATCAAATAAAATTACTTTTATTCGCATGTTAATGTATTTATGTGCTGCTACAACAGGTGATTGCACGCACTACGTCAACGGACAGTTCCTGGCCAACGAGCTGGAAAAGGTTCCGTTAGCTTGTTATAACTGTGAGTGGTACCACGAAACAGATGCCTTCAAAAAGACATTGAGAATGATCATTATGCGCTCAAATAAAAAATTCTGTTTCCAAATATCCTGGTTCACAGTAATGTCCTTGGCTACATTGATGGGTATATTCAAAGCGAGTGGATCATATTTTGTACTACTTCGAGATATTGATGAAACTTAA

>BdorOR63a-2

ATGTACAACGCAGCAGAGTTTCAAGAATTGAAAAACAACAATCGCTTCAAAATAAGAGAACTCAGGAAGGTGTCATACATTTTAGGCATAAACTATGGCTCCGAAACGTCATTGAAGAGATTTCTCCGAGTGCTTAATCTGTTCCTTATCATCATTTGTGCCATATCATTGTATCCACGATGGTTGATGCTAGAAAGGGCTGATGGCAATGTGCCGCTAATTGCAGAGACAATCACTACCATGTTACAAACAACTACAAGCATGGTTAAAATGACATTCTGCCTGTTCATGCAAGGTCAGTGTCGTGCATTGCTTAAGAAGGCTGAAAACTACGAACTACTGCAAGGAATTAAAATCTTCCTGACTGATATGGACATCAAAGCTGAGTTGAAAGTGGAGATTAACGCCATTATGGCAACTATTTGGAAGGAATCAAGGCGACAGCTTTTAAGCTGTCTCATAACTTGTTCGTGTATTCTTAGCAACTACTTTCTCTACGCCTTCTTCACTAACTTGTATCATCAAATAAAGAAGACGCCGAACTATGTGCATATATTACCTTTCACTGGTTACCCCATGTTTTTGGACAAAGGTATGGCCTCGCCTTATTATGCCGTGGAAATGTTCATCGGCGGCTGTTCTCTTCTCACCTGTGGCATGTGTCCCGTCAGTCTTCATTGCATTTTTATGATCCTCTGCAAACACGCTTGTGGTCTAGTTAAGGTCCTTTGCGTCCTTCTGATGCGATCCACCTCACTCCAAGTGCCAGCACACCGGCGTGATGAATATTTGCGTTATTGTGTTATCCAACATCAACAGACTTTGCGGTTTATAAATGACATCAATGACCTTTTCAAGCACATTACCCTTTCACATTTCCTTCACAGTTTGGCAATATATGGACTTGTGCTTTTCGAAATGAACTTTGGACTAGAAACAGATAAAACAACATTTGTTGGTATGCTTATGTACATCGGAGCTGCACTCACTGTCGATTCCATGTATTATGTAAATGGGCAATTTTTGGCCACAGAGTTGGAAAAGATTCCTTTCGTTTGCTACAGCTGCGATTGGTTTAACGAATCAGAGGATTTCAAGAGAACATTGAAGATGATAATTATGCGATCCAATAAAGATTTCTGTTTTCAAATTTCCTGGTTCGGTATAATGTCCTTGACCACATTAATGGGTATATTAAAAGCCAGCTTTTCGTATTTTTTGATTTTTAGAGATATGACGGATGAGACAAACTAA

>BdorOR67c

ATGATGCCGTCATTTAAGAGCAGTGAACCCGCACCGACTGTACCGGATTTTGTAGATATACCATTATTTCAAATAAAATTTATGGGCGCAAAACTTTTCAAATGGACACCGGATGAACCGAGAAGCAAACTGCAAATAACCTTACTAGGAACATTTTGCGCTTTTGCCACCTTCAATTTCACAAGCATGTTGCTCTTCGTCATCAACGACGAGTTAGCGACATCTCTAGATATAACTGAGTTTATACTGTTTTGGGGTTTTGCGCTCAATGCAATGATGAAGGGCGGTACAATGGTCTGCTTTCGACGTGACATCGAATTTGTACTAAAAGGTTTAGTTGCCAGACATCCAAAAACGGAGGAAGAACGCGAGGCTTTTCAATTGGTGCCATACTTTCGAACAATAAATGCATCCAATAAATATTTGTCAATATGGCATTTAAGTATAACATCAATATTTGCTCTTCATCCAATGGTGTCATCGTTACTTCGATATATATGGCGTGACGATACAAATGAAAGTTACGACTTCACGTTTCCCTTCATGATGGCGTATTACTATGACACAAATCAACCGTTAACTTATGCCGTTTCATATTTCATACAATGCTGTGGCGCATTTTATATGTCACTGCTGTTTTTGAGTGGCGATTTATTGCTCATCTCCATGGTACAACTAGTTAATATGCATTTCGGATATTTGATCTATAAAATAGAGAGTTTTCAACCAACCGGAACAGATGCTGATATGAGGACGCTGGGACCGTTGCTGGAATATCACAATGAGATATTGGATTATGCCGAGCGAATCGATAGCACTTTCAGCTTGGCCACATTTCTCAACTATGTCGGCTCATGTTTGGTACTATGTCTCATTGGTCTTCAAATAGTTTTGGGTTCAGAGGCTCTGAGCGTTATTAAATTCATCGGTTTTCTTGTTTCAACCATAGTGCAGGTCTTTTTCGTCTCCTATTTCGGCAATAACCTGAAAGATTTGAGCACTGGTATAAGTGATGCGTTCTACAATCATCCGTGGTACGATGCAAATTACAAGTACATGCGCATGCTAGTTCTGCCCATAGCACGTTCTCAGCGTTACGCTCACTTGACGGCTTTTAAATTCTTCGAAATATCAATGGATAGTTTTAAGTCGCTCTGTACCACTTCTTATCAATTTTTTACACTTCTGCGGACAAGCATGGAAGAAGAGGATAGTTAG

>BdorOR67d

ATGACTATTAAACACATCAGACCCACCGCATCCTTTGCTAAGCTCGTTAAGACGGTTCGCTTTATTTCCAGCTTGGTCGGCGCGGATGTCTCCACGGTGAATTATCAAGTCAACATCATCACTATTATTGTGATAATCTGCATTATCATGTACTTCATCTTCACCGCAACCACAGTGGCCAGTGTCTTCTCGGAGAACTGGACATATCTACTGGAGGCATCATGTATGTTGGGCAGCGTATTGCAAGGCATCACTAAACTTATTTCGGGCATCAGCCGCACCAAAGAAGTCTCCGGCATGCGCTTGGAATTGGAAGAACTTTATCGTGTATATGAGTCCAAAGGAGAATCATATTGTAAGGTAATGAATGCGTGTTGCGAACGTGTGTGGCAGCTCATAAAAATGGTGGGACTTATTTACGGTGCGGCAATTGTTGGAAATCTACTGCTTACAAGCTTTATGCTATTCTTTACAAATCAGAAAATCTATATTATGCACTTCTTCATACCCGGCGTGGACGTTGAGACGTCTTTCGGTTATCTATTGACAACAGCTTTGCATTCGTTGTGTTTTCTAGCTGGTTGTTTTGGCCTCTTTGGAGGTGATTTATTTTTCCTAATTTATCTGGGGCAACCCGAGTTGTTTCGCGACATTTTGATATTGAAAGTGCACGAACTTAACGAAGCGGCCGCTCAAAAGGACAATAAAACTGAAAGCCTATTAATTAGTATTATTGAATGGCATCAATACTACACGGATTACAATGAACGTTGTAATGAGATATTCTATTACATAATCACTATGCAAATTCTGACGTCGGGCGTCTCAATCGTCTTCACAATGTACATCATACTAATGGGCGACTGGCCGGGGGCGTATTTGTACATTTTAATAGCATTAAGTAGCCTCTACCTGTACTGCATTATTGGCACGAATATACAAACGTGTAACGAGACATTCTTTGAGGAACTTTACAACATAAATTGGTACGAATTGGATGTGAAAGAGCGCAAATTGATGATACTAGTTTTAATGAAATCACAAAATCCAAGCGAGATCAAAATTGGAGGCGTCCTGCCACTCTCCGTTCAAACTGCACTTCAAATAACAAAGACAATTTATGGCATTTTCACTATGATGTCGGGATTTTTGGATGAAGAACAGTAA

>BdorOR69a

ATGTCAGAAATCTACTCAATGAAGCATTGCTTGAAGTATCCCTACTTCACCTTGGATCTGGCTGCAACGGAACCCTTCACTTGGAGCGGCGCACGCACGTACAGCTATCGTAGGATCTGGCTTAGGCGCGCGCTCCTCACTTTCGGCGCTATAAATTTGGTTTATCAGAATATCGGTATGCTTATTTATCTCTTCATGCCGCGCGAGAGCAGTGCTCAGTCTACGATCGTGCAGGTAACCGAAACTGGGGGCATTATGGGGCTGACCATGGTCGGAACATCGAATATGCTCGTCATGTTTTGGTATGGAGACCGCATAGCGATGCTTTTAGAGAAGTTTCAGCAGTTTTTTCCTACGGCACGCCTCCAAAGCAAGGGCAAGTTTGCGAAACAATCGCTAAGAGGTGTGGAATTTCCACATCGCATTGAGCATTTTGTGTTGAAGTCCAATAAACTGATGAAGCTCGCGACTACACTTTACATGTTCGCATTTGCTTATTACAATTCCCTGCCGATTGTCGAGTTTTTGTACGAGTGGACGACACCGGGTATCGTTTGGAAATATCGTTACCAGTCCAATACGTGGTATCCCTGGCAAAATGAGCGTAACGCCAAATCATTTGCATCATTTACGCTCGCTTATGTTTGCCAAGTGCAGTCGTCGCTGACCGGTGTCGCCTTCATTATGGCCGCTGAATTTATGCTCTGCTTCTTCACGACACAGTTGCAAATACATTTCGATTACTTGGCCAATGCGCTGGAGACAATCGATGCCGCTGGCGCAAATGCTAACGAGGATTTGAAGTATTTAATAAACTACCACAGTCAGTTGCTGAGCTACTCCAAGGAAACTAATGCTATCTTCAACGTATCATTTATGGTGAATCTATGCACTTCAGCCATAGCGATCTGTTTGATGGGCTTCTCCATGGTCATGATCAGCTTGGCTCATGCCTTCAAATACTCCATTGGTCTGACTTCATTCATTGTTTTCACGTTTTTCATATGTTACACCGGCAAGGAGCTAACGGAAACGAGCGATAAGCTTCTGAACGCGGCTTTTTATGGCAACTGGTATGATGGAAACTTGGCTTACCGAAAAATGATACTATTTTTTATTATGCGCTGTCGCATACCAACTGAATTGAGAGCATACAAATTCACTACAGTCTCGATGCCCACCTTTACCGCGATTTTAAGATCGTCATATAGCTTGTTCACATTTTTTCAAGCCATGGGTCAGTAA

>BdorOR74a

ATGCGTTACTTACCGATAAGCTACCATAAACCTCTACTACCAAATGGGCTCCATCCTCCGATCGATTGGCAACTCTACGGTTTTTTCTGCGCAAATGGTTGGCCACTTGCAGCACATATTACGAAAACCCGTTACATCGCCGATATAATGGTGACCATTATGCAGTTTATGTCCGAAGGCATGGTCCTCATTGGTGAAGCTGTAGTCATGCATGACAATTTAGATAACATCAGCTTTGTCTGTACCGTCTTGGCACCCAATTTAATATTATTTGAAATGATGTTGCGCGCTTATAATATTATCTATAGGCGCAATAGTTTTCGAACACACATTGAGGAGTTTTACAAGAAGATCTATATACAACGTACCTGGAATCCGGAGCTTTTCGAGAAGATACGCCGCCAACAATTGCCCACCAAGTATTCGACCTTCACCTATATTATTACACTGGTGACCTATGTCTATGTGCCGGTAAGCGGTTTAATCAAAAATGAGCGCTTGGTACCGTTTCCGATCAATTTTGGGTTCGATTATACTGTGCCTTGGCCGCGTTATTTAGTATTTCTCACTATGTCGATGTGGACAGGTTTCGCTGTTGTGGGTCCCTTGGTCGCCGAAGCGAATATATTAGCTATGCAAATATTACATTTGAACGGTCGTTATTCATTGTTACTAGAGGATTTACGCAATATTTCTAGGGAATCCATTGCCGAGCATGAGAAATGTAAGCGGAAAGACAATATGTTGGTCACCCAACGCTTTCGTTATCGTTTATACGACATTATTCGTCGAAATGTGGAGCTGAATGACTTCGCCAAATCAATGCAGGAGCAGTATTCTTTCCGTGTGTTCGTTATGTTGGCCTTGAGTGCGACTTTGTTGTGTGTTTTGGGATTTTTAACGGCAACGCTGGGTATAACTGCGCAAAATATTCGATTTGTAAGTTGGATAATTGGAAAGGTGGTTGAATTGCTGATCTTTGGACGCCTGGGCACAACACTTTCGACAACCACAGACAAATTGAGCACATCGTATTATTGCTGTGATTGGGAAGATATTATACTTCATTCAACCAACGCGGAGGAGAACAAGAAATTGATGAAGCTTATAGCCTTGGCGATACATTTAAATAGTAATCCTTTTCGCTTAACTGGTCTTAATTTTTCCGTTGTCAACTATGAAACAGTGGTAGCGATTTTCAGAGGCGCAGGTTCCTACTTTACAGTCATTTATGCCTATAGATAA

>BdorOR88a

ATGGCGCCGCAACAGGAAGTGTTTGGCGCGAAATCGAAACTTTGTGCAATAGAAGATCTCTGCGCTATTGAGCATCCGTATCAACGTTACCTCGGTCTTAAATACGTAGAGTTTAAGCGTGTTAATGGACGTCTAGTAATACCGAAATCAAATATACTAAATTTTTTGCTATTTCTGGCAGTGGTGGACTGCACGGGTAATGTCATTAAGACTGCCATAGCCATCAACGACAGGGATGTCACTAAAGCACAAGAAGTCTTCGCCGTTTTCGGCATGGGCTTGGTAATGACGATGCGTGGTTTTATGTTGGGTCTAAATCGTGGTAAACTCTTGAAAATGTACAATGCTATCGATCGTATATTTCCACGTAGCGAACATCTGCAGCAACATATGGAGGTCGAGAAGGTACATAACTATATAAAAAAGCGTTTCTTCTACCTCCATTGGTTTTTGACGGTTTCCGTGTGCGGCTTCATCTTTATGCCGTTTGTGAAATTTATGGCATTTCACGGTTTCAAATCAGACGCGCCTGTAAGCGAAGAATTTCATGTGTATGCTTCGTGGTTACCGTTTGGTGTCAAAGATAAGGTTTCTACTTATCCCTACATATACGTGTATGAACTGTTTTTAGCTACGGCAGCGTCTCATATGCTAGTCGTTTGGGATCAAATATTTGTGATTTTAATTTCACAGTTATGTATGTATTATGAGTATTTGGGTAAACTTTTAGCGGAAATGAATGTGCCGGATGCTATGGATCCCACAAAATCGGATGCAGTCTTCAAGCAGCTACACGACTACATATATATGCATCAATATCTCAATAATTTGGCCGTCCAATTGAATGACTTGTTTAATTTTTCAATATTATCCTCGGATGCGGGCATTGCTATATCCATATGTTTCAATGTGGTTCTAATTACCGAGGCTAAAAACAATTTGCAAATCATAAATTATACTATCCCGTTATTCGTAGAGGTTTGGTTGATCTACGATGCCTCCAAGTGGGGTCAAATGCTGGAGACAGTGACTGCCCGCATAAATGAAAGAATCTACGAACAGCAGTGGTATGACAGCTCGATACGTTTTGGAAAATATACGCTGATGTGGATACAAAGCACGAATGTACCGTTCAGATTAACTGTTTTCAATCTGTTTTATGTCAATATGAAGCATTTTCAAGATATGATGATATTAGCTTATCAATTGCTAACTTTTTTGAAGGCAAAGGGTTACACAAAATGA

>BdorOR94b

ATGGCGGTCAAAAAGTGGTCACCACGCAACACGTCCTCAATGTCGAGAACCGCTTCAGCGAACATCATTATTGCTGTGCTGAAGTCACTAGGCTATTGGCAATGGACAAGAGACCCACGCCAACCATACATCGAAAAAGTCGAACGTGCATACCGCATTGTGCTGCACACAACGTTTCCATTCACTTTCATTGCGTTAATGTTGACGGGTGTGTTATTATCGCGGGATCTCGATGAGATCGGCAGTATACTACACGTATTGCTAACCGAATTCTCCTTGATCGTAAAGACATTGCATATTTGGCGAAAAGGTGGCGTAGCCTGGCGCTTTATGCACGAAGTGGCCAACGATCCCATATACGATTTGCGCCAACAATCCGAGTGGACCAAATGGCAGCAGGCCCAGCGTTCGTTTGCCATCGTTTCGAACACATATTTTGTGGCCGCGACCACCGTTGTCGTGTTCGCTTGCATCGGTGCCATGATGACACCAGCCGATGTCTATGTTTTGCCAATGAACATTTATGTGCCCTTCGATTGGCATCATCCGCGTAGGTATTGGTATGCATGGACCTATAACACCATTGCCTCATTGATGACAGCCACCGCCAATGCTATGTTGGACTTGGTAAACTGCTACTTTATGTTTCATCTGTCGTTGTTGTACAAATTGATTGGTTGGCGTCTGAGCGCTTTACGGCGAAGTGCAAACGAACCACCAGTGATTGAGCAAATGTCCGAGATCTTTCAAATGCATATGAAAGTAAGAAGATTGACGACTGAGTGCGAGACTTTGGTATCTATTCCGGTTTTTTCGCAAATTATTCTCAGCTCTTTCATACTCTGCTTTTGTGGCTATCGGCTGCAGCAAATGGAGATCATGGAAAATCTTAGCATGCTTTTTAGTACAGTTGAATTCGCCACAGTGATGGCTGTGCAAATCTTTTTGCCCTGCTACTTTGGCAATAAGGTAACCGAGTCCTCAGATGCTTTGACAGATGAGATCTTCAACTCGGATTGGACAACATTTGATGTGCCGACGCGCAGATTTATGATTTTATATATGGAACTTTTGAAAAAGCCAGCTAATTTGATGTCTGTCAACTATTTTATAATTGGCGTGGATATTTTTGCAAAGACCATGAAGAATGCCTACAGCATTTTTGCGTTAGTCCTCAACATGAATAATTAA

>BdorIR40a

ATGGCGTACATTAAGGAAATCACAGGCGACAACGAGATGACAAGGCGTTTACTGCGACAGCAACAACAGCAGCAACGTCAAGGTGAAGTCCAGCAGATGCGCAGTGGCAGAGTGCAGGGATTGGCGGAAATACCAAACAATCTTTTTGATAAATGCATTTGGTTCACAGTGCAACTGTTTCTCAAGCAATCTTGCAAGGAGCTTTATCATGGCTATCGTGCCAAGTTTCTCATGATCGTCTACTGGATTGCGGCCACCTACGTGCCGGCTGATGTCTACTCCGCCCAGCTAACGTCGCAGTTCGCGCGTCCGCCACATGAGGCACCAATTAACACGTTGCAACGTCTGCAGAGGGCCATGCTGCGCGATGGCTATCAACTGTTTGTGGAGAAGGAAAGCTCGTCGCTGGAAATGCTGGAGAATGGCACTGAGGTATTTCGCCAATTGTATGCGCTCATGAAGCTTCAGAATCCGGATATGGAGGGTTATCTCATTGATTCCGTGGAAGCTGGCATTTTATTGATCGCTGACGGACTGGAAAATAAGGCGGTTTTGGGCGGACGAGAGGCACTCTACTTCAATATACAGCAATTTGGTTCCAAGACATTTCAACTCAGCCATAAACTTTATACGCGTTATTCTGCGGTTGCAGTGCAGATAGGTTGCCCTTTCTTGGATAGTCTTAATGATGTCATCATACACCTTTTCGAAGGCGGCATCCTGGACAAAATGACAAATGCCGAATATGCCACTCAATCGCGTATGTTGGGTAAGGAATATAATGCACTACATCTCACGAATCCCTCTGAAACCAACGGTAATAACGAGCCGCCACCGAGCGATGATAATCGCAATGCGAACGGCGGTGGCGATATTAATGGCAAGGGTGAGGAGAACACGGAGGCCACACCGAAATCTCTGGACTCACAAATCATACAACCGCTCAACCTGCGCATGTTGCAAGGCGCCTTCATCGTCCTAATCTGTGGCTACGCGGCGGCAACTGGTATTCTTGTATTGGAGCTGTGTTGTCATCGCCTGAACTCGAATTTTATGGAGCGTACTCAGGCCCGCTTGTTGCGCCGTTATCGCTGGTGTAGTCGGAAAGTCCGTAGAATGACACACATGCTTTTTGTCAGAATTATGCGTTAA

>BdorIR41a

ATGTTGCCAGCTAATTTATTGAACAGTTTCTATTGGTCACGCATGTTTAATGTGATAATGCAAAACTATCTCCTGTCCACCACGACTTGCATCATTTGGCCAGAGAATGCGGATTTTTCCATTAGCTGGCAGCACGCCAAACCGCCAGATGCCGCCATCATAAATATTCGGCTACGTGATTTGAAGCAATCTTTCGCCAAAGACGTTGTAGACTTTGCAGCCAAGCGTGAGGAGCTGCTTAATGATTATGTGGTCTTGAATCCATTCGTGGAGAAATTGACTTTGTCAATAGAAAAGTCACATTGCCAGAACTTTATAGCCTTTCAATTGGATATACCAATTTTCATTGATGCCGTTATAAATGCCAGTCGTTTTTCGATTTGGCGTTCGTCAAATAATAAATTTTTGTTTGTCTACAACAAAGACGATTTGCATGACGAACTTTTCGAGCATCGCTTTTTCGAAGATCAATCTGGCATACTTTTAGTCGAAAGATACATCACTGATCCGTCAATTTTCGATTTGAAAACAAATAAGTTTGTCGGTCCACGAGCTGAGAACCCGAAACAATTGTATCTACTCGATCGTTTCAATGCTGAAAGTAATACATTTCTTCATGGCAATGATCTCTTTCCGGATAAGCTCTCCAATTTACAGGGCCGTGAAGTAATTTTGGCTGCTTTCGATTATAGACCGGATGTGGTTTTGAAATATTATCCTGGCGCTCCATCTCGTGACAGAGCCTTTGCACCCAATGATACAAGTGGCGATGTGGAAATGGATGGCACGGAAGAGCGAATTTTAAAAACATTCTGCGAAAAGCATAACTGTTCTGTAGATATTGATACGTCTGAAGCCGATGATTGGGGTATTGCTTACCGTAATATGACTGGAGAGGCGGCTTTGGGTATGATAGCCAGGGGCAAGGCAGAGGTTGGCATGAGTGCGATGTACACATGGTACGCCGATTACGTTGCGCTGGACATGTCGATGTATATTGGACGTTCGGGTATTACCTGTGTGGTACCGGCGCCCAAGCGCCTGGCAAGCTTGTGGCTGCCGATTGAACCCTTTCAGCCAGCACTTTGGGCTTTCGTTTTCGTTTGTTTGTGTGTGGAGATCATAGCGTTGCTCGTCATAGATCACGCACGTCCGATAATTATAGCATTGAGTGAAAGGATGCGTGCTTCGGAGGAAAAGAAAAATAGTTGGGCTCGCAATTTCGAGTATGCCTTCTCGACGACGATGTTGCTGTTCGTCTCGCAATCGAATAAGGGCACCATGGTGAATTTCACGCCATTGCGATTAATGCTTTTCGCTAGCTTCTTGAATGACATCGTTATAACGAGCATTTATGGTGGTGGACTGTCAAGCATACTCACCGTGCCAAGCTTCGGCCAAGCTGCCGATTCGGTGGAGCGACTTTACGCTTTCCAACTCAAATGGGGCGCGGACTCTGAGGCCTGGGTGGCGGCAATACGTGACGATGAGAGCGAAATCATGAAGGGTCTGCTGCGTAATTTCGATATTTACAGCGCTGAACAATTGATGGTGTTGGCGCAGACGGAAGAGATGGGTTTTACCATCGAACGCCTGCCATTCGGTCACTTCGCCGTACAGGAGCACTTGACACGTAGCGTTTTGGACAAAATGAAAATCATGGTCGAGGATATTTATTTCCAGTACACGGTTGCATTTACAGCGCGCATGTGGCCTTTGCTGGAGAGTTTCAATGAAATGGTAGTCATGTGGCATTCGTCGGGACTGGACAAGTTTTGGGAGTGGCGCATTGTTGCCGACAATTTGGATGGCGCCATACAGAAGGAGCTGATGGCCTCGCAGTATTCGAACTTGGATGACATTGGACCGGTTAAGTTGGGCATGTCGAATTTTGTGGGCATGTTGTTGCTGTGGTTGTTGGGTATAACTTGCGCCTTTTTGGCTTTCTTGGCCGAACTATTGTTGGATCATATGAAGCGTGCCAAGAAAGTGGCTGAGAGTGACGTTATTGAAATAAGAGAGGGAAGTCTTTAA

>BdorIR75d

ATGACTCCACACTTCATTCTACTAATTTGCCATATATTTCTGGTGGCCATTTCAAGCGGCGCTGAGCCCAGCGGTTTCACAGGTGCCGAGCAACTAGGCAACTCAACACCGAAAGAGCCAACTGGAGCAGGTTACGATGCGCAAATTTTCATGGAATATTTTCGTTGGCATGGTGTCCACAACATAATGTTGATTGTCTGTCCGCAGGATGTAGGCACCAGCGAAAGGCATCATAAATTGAAATCGCTACTGCGCCAATTTATCGCTCATGGCTTTCCAACACGCGTATTCAATGGCCAAGACTATGATGACAACGAAAGCACTCGCAGTACGGGGCAAGCGCAAGCGCAAGCTGAAGCCGAGCCATTCACTACGACGCACAACTCAACGGCAGATTCAACAAGCGCCGTACTTGCAATGAACCGCCCCACGTTCGGTCCACCGCGCACATTCCGTAGCGATAACAACACGCGTCGCCCATTGCGTTTGCAGCTGCCAGCATTCACCTATAAATCAGGTATACTGTTGTTACAATTCGCCAGCGCCTGTTCACTCAACGTGCTACGCTGGGCCGCTGCCGCCGAGCACAACTATTTCACCACAAATCGCTTTTGGTTGCTTTTCACCGATGAGCCGACACATATTTCACTGCTCGACGATGGGGACATATTTCTGCCGCCGGATGGCGAGGTGCGCGTCATGTTGCACCAGCCAGGCGCGCAATTTTTCACGTTGGTGGACGTGTATAAGGTAGCCGCAGACAAGCCTTTACGTCGCACCCTGGTGGGTGGTCGGGAATTGCGGGACGCAGAGGATATGTTGCAAGCGCTAGGGAAATTCGGTTCGGCTATTTCTTATCGTCAAAATTTGGAAGGTATCACTTTCAAGACGGGGCTTGTGGTCGCCTTTCCTGATTTGTTTACGAATATCGAGGATTTGTCGCTACGTCATATCGACACTATTTCAAAAGTAAATAATAGGCTAACCCTGGAGTTGGCGAACAAATTAAATTTGCGCTTCAACACGCATCAAGTCGACAACTATGGCTGGCATAAACCAAACGGCTCGTTCGATGGACTAATGGGACGTTTTCAACGATACGAACTGGACTTCGCCCAGATGGCAATTTTCATGCGACTTGATCGCATCGCCATTGTGGATTTTGTAGCTGAAACATATCGGGTACGCGCTGGTATAATGTTTCGCCAACCACCACTATCGGCCGTGGCCAACATCTTTGCCATGCCCTTTGCCAGTGATGTCTGGATTGCTATATTACTGCTTATGATCTTCACTATAGGTATATTCATAGTGGAGTTAGTATATTCGCCACATTTGCACGAGATGGATATATTGGATTGTGTGGTCTTCGTTTGGGGTGCGATGTGTCAGCAGGGTTTCTATGCTAACCTACTCAACCGCTCCGCCCGCGTCATTATTTTCACGACATTTGTATCGACACTTTTCTTGTATACGTCATTTTCGGCGAATATAGTGGCGTTGCTGCAGAGTCCTTCGGAGGCCATACAAACTTTAAGTGATTTAACGCAGTCCCCGTTAGAGGTCGGTGTACAAGATACACAATACAACAAAATATACTTCAATGAGTCCACCGATCCCGTTACCAATCACTTATATCACAAAAAGATTGCACCCAAAGGTGAAAATATTTTCATGCGCCCATCAATAGGCATGGAGAAGATGCGGACGGGTCTCTTCGCTTACCAAGCGGAATTGCAAGCCGGATATCAGATCATAAGCAACACCTTTAGTGAGCCAGAAAAGTGTGGACTCAAAGAGCTGGAGCCATTTCAGCTGCCGATGATTGCAGTTCCAACCCGAAAGAATTTCCCATACAAGGAACTCTTTCGTAGACAATTGCGTTGGCAACGTGAAGTGGGCTTGATGAACCGTGAAGAGCTGAAATGGTTTCCGCAGAAACCGAAATGTGAGGGTGGCATGGGTGGTTTTGTTTCGATCGGCATCACTGAATGTCGCTACGCTTTGGGCATTTTCGGTTTCGGATTACTACTGAGTGCTTTTAGCTTCATTCTGGAATTGGTCGTTAATTATGTGTGGAATTTAGCAAAGAAAATACATCGAAATAAGAAACAGCGGAAGGAAAGTAATGCTGCTGATGCGTATCATGGTAATTTTGTACATTAG

>BdorIR76b

ATGACCGGCTTCGACTTAATACTTTCGGCGGCGCTGTGTCTCACGTGTGCAAATCTAACGGATATACGTCTACCCGAGGGTCTAATAGAGTTAGATGAAAATAATACCGTTGTTACTATATCGCCCGATTTGGCAGTTGACGAGCCATCCTTGGATGATGCACCACTGGAAACGGTAAAAACAATCATCGCTAAGAAGGAGAAAATGGATAAGCTAAGAGAGTGGATAAAGGGTCGCAAATTGGTGATTGCTACGCTGGAGGATTATCCGCTCAGCTATACGGTTATGGAGAATGACACGCGAGTGGGCAAGGGTGTGGCGTTTGAGTTGATCGACTTCTTGCAGGAGCAGATGCAATTCACCTATGAAGTTGTGGTACCGGAAGATAATATCATTGGTTCGAGAGAGGACTATGAGAAGAGTCTTATAAAGATGCTGAATAACTCGGAAGCCGATTTGGCCGCTGCTTTTATACCGACGCTCAGCGAGCAACACAGCTTCGTTTTCTACTCGACAACCACTTTGGACGAGGGTGAATGGATTATGGTGATGCAACGTCCACGTGAATCGGCCACTGGCTCGGGTCTGATGGCACCATTTGATTTTTGGGTTTGGATCTTAATTTTCATTTCGCTATTGGCGGTTGGACCAATTATTTATATGCTCATTATATTGCGTAATCGTTTGACTGGTGATAAGGAGCAGAAGCCTTACTCGCTGGGTCACTGTGCCTGGTTTGTGTATGGTGCATTGATGAAGCAGGGCAGTACACTGTCGCCGATCGCAGATTCAACACGCCTACTATTTGCCACCTGGTGGATATTCATTACGATATTGACTTCATTCTACACCGCAAATTTAACAGCTTTTCTCACGCTATCCAAATTTACTTTACCCTATAATACCGTTAGCGATATACTATATAAGAATAAGCACTTTGTTTCGGCACGCGGCGGCGGCGTGGAATATGCGATAAGGAATACCAACGAAAGCCTTTCCATGCTGACAAATATGATACGCAACAATCACGCCGTATTTTCGAGCAGCTCGAACGACACATTTAATTTGCAGAATTTTGTTGAAAAGGATGGCTACGTATTCGTACGCGACCGCCCGGCTATTAATCATGTGCTTTACGCAGACTATCGCTATCGCAAAACCATTAGCATGAATGATGAGAAGCTACACTGTCCATTCGCTATGGCGAAGGAGCCGTTTTTGAAGAAGAATCGTTCTTTCGCCTATCCGTTGGGCTCCAATTTGAGTGAGCTCTTCGATCCCAAACTACTCAACCTCGTGGAATCTGGCATCATTAAGTACCTGTCGACTAAAGATCTACCAAATGCCGAAATATGCCCGCAGAACTTGGCCGGTACCGAGCGTCAGCTACGAAACACCGATCTCATGATGACTTACTACATTATGTTCGCCGGCTTTGTCACCGCCATGGTTGTTTTCTTCACCGAGCTTATATTCCGCTACCTGAATCAACGTAATGAGGGTAGCAAGTGGGCGCGTCACGGCGTCGGTCGCACTACAAATGGACTTTCGGTGCGTGCACCGCGTTGGTTGCGTCAATTGGAGACGGACAGCGACAAGCAACGTCTGACTGCTTCACCCTCGGGTTCGACCATTACACCACCGCCACCATATCAAAGTATTTTCAGCAGCAATCATCGCCATCACCAGCAGGATGAGGCAGGCTATTTGAGTAAGGAAAGCAGCTTGCATCGTTGGCGACGCGCCGGCCAATTCGGCGCCGGTGGCTCGAATTTTGGCACTTTAGCGGCCGGTGCAGGTAGCGGTGCTGGTGTACTACTGGGCAATGGTCAGTTGCATGATGGTAGCGGCGCGGGTGGTGTACGACGTTTGATTAACGGTCGTGATTATATGGTTTTCCGCAATCCGAACGGGCAGAGCCAATTGGTGCCGGTGCGTGCACCCTCAGCGGCGCTCTTTCAGTATACTTACACGGAGTAA

>BdorIR84a

ATGTCCAAGCAACGTACGGTGACGGCAGCGCTGGCGAATGTGCGTCGCGTGATGACGCCGCTGCTACTGAATATAAGTGCGGACATTACAATCGGTGTCCGCTTGGATGACAACAATATAATACAACTTTTCGACATTTACAAGATACAAAAAGATTGGTTGGATATTGAACCGAAAGGTTATTGGAGCCCCGCAGAAGGTCTCAAACTTAATTTGCGGTTCCATCAAACCTTCGTCAATAGGCGTCGCAATTTTAAGGGTCTACAATTAGTAGGTGGTATTGTGATACGGGAACAGCCTGCCGATATGGCTGACTTGGACTATTTGAACTCCTTGTACCATAAAAATTTTGACCCCATGCAAAGGAAAACTTACCAATTGGTTAAATTAATGGAACCTGTATTTGGCGTAAGCTTTCAACCAGCACTGAGAAAAACTTGGGGTGAGCAGGCGCCGAATGGCAGCTGGGATGGTGTTATGAAATTATTGCTTTCTGGTGAGGCGGAATTTTCACTATGCCCCATGCGTTTTGTACCAAACAGGGTACATTTAATACATTACACGATAGCGGTACATACCGAATTTGTCTTCTTCATATTCCGTCATCCGCATCGCAATGACATTCATAATATATTCTTTGAGCCGTTTGTCGAAGAGGTCTGGTACACTGTTATCGCGATTGTTGCTCTAACCACATTATTGTTGCAACTACATTTGCATCACGAAAATCGCTTCTTCATAAACAAAGATCCACATTTTCAGACGCGTTTCGATTATGCGATTTTCTCCATATTGGAAGCATTTTTCCAGCAAGGACCCTCAACCGACGCTTTCACCGCCACCTCAACGCGTACGCTCATTTTCTCTGTTTGCCTGTTTAGCTTGTTGTTGCAACAGTTTTATGGCGCTTACATTGTGGGCTCACTGCTGTCCGTCTCACCGCGCACAATAACCAATTTGGAGGCACTATATAACAGTAGCCTCGATATCGGTATAGAAAATATACCATATAATATCGATACCTTTGAGAAAACTACAGTGCCGCTAGGTATGGCAATCTACAAAGAGCGTGTTTGCAAGAACCGTGAGAGAAATATTTTATACATCGCGGAGGGCGCTGAACGCATAAAGAAGGGCGGTTTTGCTTTTCATGTATCGGCCAATCGCATGTACTACATACTGAAAGAATTACTAACCGAAAAGGAATTTTGCGATCTGCGCATTGGTATTGGTATAACGAAAAGTTCACCGTTTCGCGAATACTTTACAACAACAATTGCGAAATTTCATACCACCGGGCTCTTGCAACATAACGATAACCAATGGCAGTTGCCACAAATGGACTGTAGTTTAAGTCAAAATTATGAGGTCGAAGTAGATCTGCAGCATTTTCTACCGGCACTACTTTTTTTAGTATCTGCTATGCTACTTAGCTTGGCTGTATTAATTTTGGAGATTATATATTACAACTTGGAGAAAAGCACGAAGTTGGCACGTCTGTGTCCAAGAATAATGCCGAAGCCCAAACTGGAGTTTATAAATTGA

>BdorIR92a

ATGTGGCCGACACACACGCAAACGCACGTCACAACTTTGTTGCAGCTCTTAATACAAAGATACTTCGCACAATTCTCCTCAGTGCTGATTGTCCATGATGGGCGCGTTGACGCAGACAGCGCCTTGCAGCGGGAATACCTCGAGGCGGTGCAGCTGGCGTTCCACAATCTGTCCCAGCAGGGACGCCTAATTGGACTGCAATGGATTGATGTCAGTCAACTGGATGTCGGTGGCGGTGGCAGAGGCAGCTGCCGCAGCAGCAGCATCAATTGCGGAGACAGTTACAACAACACATGCGCTGACAATTTGGCCTACAACGATGAGTTGGAGCTGTGTGTGCTGCGCGCCGTTGACATTGTAACCGAGGGTTTCATCACCATTCTTTCGGACACAGTACGTTTCCTGCACGCTCGGTACTTTGCCACACGCAACGCGGAGTTACGCCTCAAGGATAAATTTTATCTCTTTTTTTGTGAGCATGAACGTCCCGAGGAGTTGCTATCAACCGAGATACTGCAGTTTTACCCGCACCACCTTATGGTGACACCGGAAACGCTCACAGCACAACAAAGCAACAACCAACAAGCAACAACTAAACTAACTAAACCAACAACTACAACAATGCCGTTATCCACAATCGCCACGGCATCTCCAAGCGCGCACCGCGACATCAACATCCAACTGTGGACACAAAAGTTTGTCGGCGCCAGCGGCAATTTGGAAGCGCTGCTGCTGGACGCATTCTTGCCGAACGAAACTTTTGCCCGGAACGCCGAACTTTACCCAAATAAAGTTAACAATCTGCGAGGGCGCACAATCCGTGTCGGCTCCATTACATACATACCATATGTGGTGGCTAATTATGTGCCAGCTGGTATAGGCGATGTTGACGCGTTAAATTCCAGTGATTACAGTCGTACTATCTCCTATTTGGGTTCTGAAGCAGAGCTTATGAAATCCTTCTGCGAAGTGCGCAATTGTCATATACGCTTGGAACCATATGGTGCCGATAACTGGGGATATATTTATGAGAACGAGAGCGCGACCGGTATGTTGGGTGATGTGTACACACAAAATGTCGAAGTGGCCATCGGCTGCATTTACAACTGGTACAACAACATTACGGAAACTTCCAACATCATTGCACGATCATCTGTAGCTATTTTGGGACCAGCGCCTGCACAATTTCCCGCTTGGCGTGCCAACATAATGCCTTTCAGTAACGCCCTCTGGATTTTCTTAATACTGACGATTCTACTTTGTGCCGCCGTTATGTATCTCATTCGTTTTGTAGCCTCGCTGCTGGACAAATGGCTTAGGGGTGTACAATGTGAATTTCAACATCTAACAGCATTTGGACAAGCGACATTGGATATGTTTGCGGTTTTCATACAACAGCCATCCGGGCCGACAAGTCTAAACACGTTTGCCGTCCGTTTCTTTCTGGCGATGATACTCTGTGCCACTATTACCTTAGAGAACACCTATAGCGGTCAATTGAAATCCATACTAACCGTACCGCTATTCACCGAAGCTGTTGATACTATGGAAAAATGGTCAAAAACGGATTGGACTTGGTCGGCGCCATCCATTGTATGGGTCCAAACTATAGACAGCTCAAATATCGAGAAAGAGCAAATAATGGCCGAGAAATTCGAAGTGCGCGACTATGATTTTCTATACAATGCCAGCTTTCGTTCAGACTACGGGCTGGGCATCGAACGTCTTATGAGTGGGTCTTTTTCTTTTGGTGATTATGTTACAGCGCCTGCTTTAGAAACAAAAATTGTATCAAAAGATGATTTATACTTTGATTGGACCCGTGCTGTCTCCATAAGGGGCTGGCCATTGATGCCGCTTTTCGACAAACATATACGCGCGTGCGTTGAAACGGGACTCTTCGTGCACTGGGAAAGAAAGATTGTGGCAAAATACTTGAATCGGCAAACACAGGAAATTATGCTGAACTTGGCCTCGGGGCACATCAACAAATTACCACCACAAAAGCTAACGATAGAAAACATTTCGGGCGCCACATTTACATTGCTCTTCGGCTGCCTGATTGCGAGCTTCGTATTTGTGCTGGAACTGACTGCTCATTACTTTAATAAATTTCAAGGCTTGTGTATTCAAAGAAATGAAAAAAGTGAAAACTAA

>BdorIR100a

ATGAACAATAGCGGCATTGAATGCTTATTTTTGAATCCCTTTCCAAAAATTGGCTCTGAACGTGGTGTGATGACTAGTTTGAGTGATGAAAGCTACAAGAGTATTTTTCTTAATTTTCATGAGTATCCACTCCGCACCTATATTTTCCACTCCGTCTATTCTGATATAGAAATTTTTATGAACGAAACGTCGAAAAAAGTAATTGGAGCAACGGGGGCTGATGCCAAAGTGGCTTATCTATTGGCAAGTAAAATGAACTTCACCATGGATCTTCAATGGCCGGACGATGGCTTTTTCGGCACACGCTCGAAGAATGGCAGTTACAATGGTGCACTTGGACGAATGATACGCTTTGAGACTGATATTATATTAGCTGGATTCTTTATAAAGGACTATCTAACGCGCGATATTGATTTTACTTCAGCCGTATATACTGATGAACTTTGTTGTTATGTGAAAAAAGCTAGTCGTATACCTCAATCCGTACTGCCTTTATTTGCCGTGAATATCGACATTTGGATTTCTTTCATATTCGTCGGAATGCTCACTCCATTTGTATGGATGTTACTAAGACGAGTAAATTTGAGTGTTATGACAACAGGATCTGTCCCATTAAAATTGCAAAAGTTGCAAACACAGGAAAGTCGTCTGCTAATGCAAAAACATAAACTGCAGTATATACGCATTTTTATAGATACGTGGGTGATGTGGGTGCGTGTTAATATTAGGAATTATCCACCCTTTATATCAGAACGAATCTTTATAGCTTCACTCTGCCTGGTGAGTGTGATCTTCGGTGCACTTTTTGAATCAAGTTTGGCAACAGTCTACATACGGCCACTGCACTACAAAGACATCAATACGATGAAAGAGCTAGATGAAGCCAATATAAGAATATATATTAAGCATGGCGCAATGAGAGACGATCTATTTTATGGTCATAGCTCACAAATATATCAGAATTTACAGAAGAAATTACTTTTAATTGGTGAATTAGAAGAGCGTCTTATTCACACGATGGCGAGAGGCGGAAAATTCGCATCTGTTACTCGAGCCTCTTCACTCGAACTCGACGACATTCACTATTTTTTAACAAAGAAAATACATAAAATACCCGAATATCCCAAAAGCTATAATATAGCTTTTTTGCTTCCTAGTCACTCGCCATTGGAAAAGAGCATAAATATATTGTTGCTTAAATTTGTACAAGCCGGTCTTATTGACCATTGGATTGCAGATATGAAGTATCAAGCAAGGATTAAAACACGAAATTTTGCGGGATACCTGGACGAGAGTGGTGACAAATGGAAAGTTCTAACTTTAAATGATCTGCAGTTGTCTTTTTATACCATCATATTTGGCAGTATGTTGGCGACAATTGTGTTGTTCTTAGAATTAATCATACACTGCAAGCAAGTTAGAGTTTTCTGCAAAATTCGCACGACGAAAGTCAAGTAA

>BdorCG11155

ATGGTATTTCTTCAAGAATTTACGAAATGCGACGTGGTTTTTATTACCACGCCAAACCTCAAAACGAAAGATAACATGGAAACGATCAACTTAAAACAGATTAGGCTAAATTCAAACCCCAAACTTAATAATTGTGTATGTTTTAACCTTCGTAGTTTCATAGGATATGAGTCTTTACTTAGCCGCTGTAAGCTATCTGTTTCACACATACTCCTAATCTACTATTTATGCAACTTTTTGTGTGCTGCATCGTTACCAGCAGTAATCCCTTTAGGTGCTATTTTTACAGAAGACCAACGAGATAGTAGTATTGAGTATGCATTTAAATATGCCGTGTATCGTATAAATAAGGACAAATTGCTATTATCAAATACGCAGCTTATTTACGACATTGAGTATGCTGCGCGGGATGATTCATTTCGAACAACTAAGAAAATTTGTCGACAATTGGAATCGGGTGTCCACGTCATTTTTGGGCCATCAGATGCATTGCTTTCGGATCATGTGCAATCAATATGTACATCGTTTGGTATTCCGCACATCGAGTCTCGTATTGATATAGATGAAAATTCTAAGGAATTATCTATTAATCTGTACCCCTCACAGCGACTCATGAATTTGGCTCATAGAGATTTGATGATATTTTTAAATTGGGCGAAAATTGCTGTACTCTATGAAGACAATTTGGGTATTTTCAATCATCAAGATCTGTTGCATGTAACTGCAGACATCCGAACAGAACTATACATTCGTCAGACGTCACCGAAAACTTATCGTCAAGTTCTACGAGCGATACGAATGAAGGATATTTATAAAATTATTGTAGATACAAATCCTAAAACCATAAATGCTTTCTTCCGAGCAATTCTTCAACTACAGATGAATGATCATCGCTATCATTATATGTTTACAACATTTGATATTGAAATTTTTGACCTGGAAGACTTCAAATACAATGGTGTTAACATTACGGCCTTTCGCTTAGTTGACGTGGAAAGCCAACGCTATAAGGAAGTTATCGAACAAATGCAAAAATTACCTCATAGCGGATTGGACTACATAAATGAAAAGCCATATATACAGGCTCAATCAGCTTTAATGTTCGATTCGGTGTACTCCGTCGCAGCTGGTCTTATGGAGCTAGATAAGAAGGATTTATTGAGTTGGCATAATATTTCCTGTAAAAACGAATTGCCATGGAGGGATGGAATGTCGCTATATAGTTATATTAATTCGGCTTCTATGAATGGATTGACAGGCCGTGTACACTTCACTGAGGGACGTCGAAATTTATTTCAAATCGATTTGCTTAAACTAAAGCGAGAGAAAATTCAAAAAGTAGGTTTTTGGAAACCTGAAGTCGGTGTTAACATTACCGATTCGACCGCTTTCTATGATACTTATTCAAGTAATACAACGTTAATTGTTATGACACGCGAAGAAAAACCTTACGTTATGGTGAAAAGTGGCATAAGTCAAACAGGAAATGATCGCTTTGAAGGGTTTTGTATTGATCTACTAAAAGCCATTGCCACTCAAGTGGGATTTCAATATAAGATTGAATTAGTACCTGATAATATGTATGGAGTATTCAACCCTGACACAAAAGTTTGGAACGGAATAGTACGCGAGTTAATGGAAAAGAAATATGCATTTGCCCATGAAAGGGAACCGTTGTGCAGCCGTAGAAACCATTCCAAAGGCTTGCACCGGCATACTGGCGGCAATATCGACCAACGCTCGTTCGAAATGTTTTTAGACCGTGCAAAAAGTTGTATTCAAGCAGAAGGAGACTATTTTGAATAA

>BdorCG5621

ATGAGACATAACATGGCCCGGCCGTGCTTGTCCCAATTTCCGACATTAACTCGTTGCTCACGCATAACTCTAATACTTTTGCTGGCATTAAGTTGCTTAAGTTGCTTGCAAATTGCAGCTTCACAGAAGACAAATGTCGGTCTCATCTATGAAAGCGACAACCCCGATATGGAGAAAATATTTCAAATAGCTATTGACAAAGCGAACGAAGAGAGCGGTGGAGCGCTCGAACTGCACGGCATCGCTGTGGCCATTGAACCTGGCAATGCCTTCGAAACATCAAAGAAACTCTGCAAAATGTTGCGGCAAAATCTGGTGGCCGTATTTGGGCCTACCACAGATCTGGCCGCAAAGCACGCGATGAGCATTTGCGATGCCAAAGAGCTGCCATTCATCGATACACGCTGGGATTTTGCTGTGCAAATGCCAACCGTTAATTTGTATCCGCACGCCTCACAGCTGGCGGTGGCGCTGAAAGACTTGGTGGTGGCGCTCGAATGGACCGACACCTTCACAATCATCTATGAAACGGGTGAATTCCTGCCGACGGTAAACCAATTACTGGAAATGTACGGCACTATGGGGCCTACAATTACCGTGCGCCGCTATGAGCTTGACTTGAATGGCAATTACCGCAATGTGCTGCGACGCATTAAAAATTCTGGTGATTACTCTTTTGTCGTGGTGGGCTCGATGGCTACGCTGCCGGAATTCTTTAAACAGGCGCAACAAGTTGGTCTCATGACTGATGACTATCGTTACATTGTCGGCAATTTAGACTTCCAAACCATGGATTTGGAGCCGTTCCAACATGGTGACACCAACATAACTGGCATACGCTTGGTCTCGCCGGATGAGAAGCTCGTGCAGGACCTTGCCAAAACCTTATACGAAACGGAAGAGCCTTTTCAAAATGTGTCGTGTCCTTTGACCACCAGCATGGCATTGGTGTACGACGGTGTTCAGTTGCTGGCTGAGACATTCAAACATGTTATGTTCCGTGCGGTGCCATTAAATTGTAACGATGCCAGCTCATGGGATAAGGGATACACGCTCGTCAACTACATGAAATCGCTGAGCTTAACTGGACTCACCGGTGAAGTAAAATTCGACTACGAAGGTTTGCGCACCGATTTCGTGCTGGACGTTATTGAGTTGACCATGTCGGGTATGCAGAAAATAGGCGAATGGAAGACGGAGGGCGGCTTCTTCGCTAATCGACCGCCGCCAAAGATAGTGGAGGTGGATCAGCGTTCGCTGGTGAATAAGAGCTTCGTGGTTATCACAGCCATAAGCGAACCGTATGGCATGCTGAAGGAGACGCCTGCTAAACTGGAAGGCAATGATCAATTCGAAGGCTTTGGCATTGAGTTAATCGAGGAGCTGGGCAAGAAATTGGGTTTCACTTATACTTTCCGTCTGCAAGTGGACAACAAATACGGTTCGTTCAATCCGAAAACGGGCAAATATGACGGCATGATGCTGGAAATAATCGAAGGACGAGCTGACATGGGCATCACCGACTTGACCATGACATCCATACGTGAAGAAGGAGTAGATTTCACTATACCGTTTATGAACTTAGGTATCGCCATTCTATTTCGCAAACCGATGAAGGAACCGCCAAAACTCTTCTCATTCATGTCACCATTTTCGGGCACTGTTTGGATGTGGTTGGGCATCGCCTACATGAGTGTCTCACTCACGCTCTTCATTTTGGGACGCATCTCACCGACCGAATGGGATAATCCGTATCCTTGCATTGAAGAGCCCACCGAATTGGAGAACCAATTTAGTTTCCCTAATTGCTTGTGGTTCTCCATCGGTGCACTGTTGCAACAGGGCTCTGAATTAGCGCCAAAAGCATACTCAACGCGCACTGTGGCCTCAATTTGGTGGTTTTTCACGTTGATTTTGGTCTCTTCGTACACCGCAAATTTGGCTGCTTTCCTGACGATAGAATCGCTCAGCAGCCCCATAGAGAATGCTGAGGACTTGGCCAACAACAAGGGTGGCGTTAAATATGGCGCCAAAGTGGGTGGCAGCACGTTCACGTTCTTTCAGGATGCTAAGTATCCAACGTATCAGAAAATGTATGAATTTATGCGCGACCATCCGGAATATATGACCTCAACCAATGCGGAAGGAGTGGATCTAGTGGAAAATGAGAATTACGCATTTATAATGGAGTCAACCACCATTGAATATATCACCGAGCGACGTTGCAGTTTGACACAAGTCGGTTCACTGCTCGATGAGAAGGGTTATGGCATTGCAATGCGAAAGAATTGGCCCTTACGTGATATGATCAGTCAGGCTGTGCTGGAACTGCAGGAGCAAGGCGTCCTTACCAAAATGAAAACGAAATGGTGGAAGGAGAAACGTGGTGGCGGTGCATGTTCGGATACCAGCAGTGAGGGTGGCGCAGTGGCCTTAGAGCTAAGCAACTTAGGTGGCGTTTACTTGGTGTCGATTGTAGGCTCTTGCTTTGGTGTGTTGGTGGCATTCCTTGAAATGGTATTGGGTGTGAAGGAACGCTCCGATGAGAATAAGGTGTCCTTCAAGACCGAACTCATCGAGGAGTTCCGCTTCGTTATGCAATGCTCCGGTAACACAAGACCCGTAAAATATCCAAAGAATTCGAGTCGCAGCCGCAGCCGCAGCTCTCGTTCCCGTTCACATTCTCGTTCCAGTTCGAAGAGTTCTACATTGTCCGTGGATTCTTTGCCGATGGATGAGAGTAAATTACATCACATTAGCGAACACACCAAGCACGCCAAGTAA

>BdorGluRIIC

ATGAATATAAATATGAAATATTGGATTATTGTGGTGTTGACTTGTCTCTGTGCGAAGCATGCACTGACTTTGCCACAATTTAAAATCGGTGCAATCTTCTATGAAAATGAATTTGATTTGGAGCAAGATTTTATCGCAACCGTGGAGAGTATAAACAATGAGGGAGTGAATAACTTCGAAATGTTGCCACTAATAAGGCGCATCAGTGAAACGGCTGGCAGCATGATCTTGCAACGCGAAGCGTGCGATCTAATTGATAATGGTGTGTTAGCTATTTTTGGACCAAGCGCGCAGGCAGACAGCGACATTGTTTCGCTGATCTGCAATGCAACCGGCATACCGCATCTACAGTTCGACATGGGCCATGAGGAGACGAACCGGGAGCGCGTCAATCATCAGATGTCGTTGAATGTCTTCCCCACACAACAGATGCTCTCCAAGGCCTATGCGGATATCGTACAGACCTACGGCTGGTGTAAATTTACCATTATCTACAATGCTGAGGATCCAAAAGCACCGGCGCGCTTGCAGGATCTGTTCCAGCTGCGCGGCATACACAACGATGTGGTGCGCGTGCGCAAATTCAAACGCGATGATGATTACCGCATATTATGGAAGGGTATAAAAGGTGAACGTCGCATTGTGTTGGATTGTGCACCGGAGTTACTGATTGATTTGCTGAATACTTCCATCGAATTCAATTTGATGGGACAGTTTAATAACTTGCTGCTAACCAATCTGGAAACGCATAATGCCAATTTAGAGGAATTGCGGGACAATGTCACCTTTGCGGTCAATATCACAGCGACACGTCTTAAAATGGATGGGAATTCTCCCTATGCGAATATTATGAATGACACAGATCCCTTATTTGGACGCAGACTTCTACAAGATTTACTCTACGACGCCGTGCATGTCTTCGCGAATGCATTACGAAATGTCAGCCATAGTTATCAGATACGAGCACCGCGTGTCCGCTGCGATTTCAATGAGTATGAGCAAATGCAACCGAGGCCCATGGGACGTTACATCTATCGCGTGATGTTAGCGACATCCGGCGTCAATAACACAGATTATCGCACGAGTGACCTGCAATTTGACGAGGAGGGCCAGCGTACTAATTTCGGTATTGAAATATTTGAACCGCTGGAAAATTATGGTATCGCCTTCTGGGATACAAAGGGGCAAATTACGCCGCAACACGTGGAGGTGAACATAACGAAGAAGTTGGTTTATCGTGTGGCAACGCGTATTGGTGAACCATACTTCATGGAGATACCCGAAATGGTGGAGCAGAATGTCACTGGTAATGAACGGTATGAGGGTTACGCAGTCGATTTCATTGCAGAGTTATCGAAACTCATGAATTTCGAATATGTATTCGTGCCAGTTGCGGATAATGGCTACGGAAGATATAATCCAGAGACTAAACAATGGAATGGTATTATCGGCGAGATTGTCAACAATGATGCCCACATGGGTATATGTGACTTGACCATTACACAGGCACGTCGTACTGTTGTCGATTTCACAGTGCCTTTCATGCAGCTCGGCGTTAGCATATTGTCCTACAAAGAGGTAACAGAATCGAAAGCTTTGGCTTTCCTTGATCCTTTAAAAGGCGAAGTGTGGATATGTGTCATTGTTGCCATATTCGTGATTTCCTATCTGCTTGTGATCTCCGCCAGAATCGCCGAAGACGAATGGGAAAATCCACATCCTTGCAACAAAGATCCCGATATGCTGGAGAACAAGTGGGATTTGTTCAATACCTTTTACTTGTCAGCGGCCTCCATAATGCAGGCTGGTTGCGATATGTTGCCTAAAAGTGCGCCATTTCGTACATTCACCGCTATGTGGTGGATAATCGCCGTTATCATACCCAATTGTTATACTGCCAATTTGGCTGCCTTCTTGACGAGCTCCAAAATGGAATCCACCGTTCAGGATCTGAAGGGTCTCGTCGAACAGGTGGATATTAAGTTTGGCACAATAGAAGGCGGTAGTACGTACACGCTGTTTGCCGAATCTAACGAGACCGTGTATCGATTGGCATACAATATGATGAAAAATGAAGATCCCTCCGTATTTACGAAGGATAACAAGGAGGGTGTAGATAGAGTGCTTAAAAATAACGGTTCGTATATGTTTCTTATGGAGACGACGGCTTTGGAATACAATATCGAGCGCAGCTGTCATCTGCGTAGTGTGGGCGATAAATTCGGTGAAAAGCATTACGCTATAGCGGTACCTTTCGGCGCCGAATACCGTTATAATTTAAGCGTTAACATTTTAAAACTAAGCGAGACAGGTAAATTATTCCAACTGAAGAATCATTGGTGGAAGGTTAACGATACGGATTGCGAGGACAATGATGATGATGCGGATAATGATTCCTTGGGTATTTACGAAGTACGTGGCATATTCTATACGCTTTATCTTGGCTTACTAGCTGCTTATCTTATGGGTTTCATTGAATTTTTAATGCATTGCCATAGTAGAGCAAGCGAGGAAGGGTTGCGTTTCAAAGAGATTTTAGTGAACGAAATGCGTTTCGTATTGCGCATATGGAATAATCGGAAGCCGGTTAGTTGCACGCCCACTGCGAGTATAGCGGCTTCATCGCGGCGCTCATCAAATAGAACCGCAAGAACATTAACGAAAAAGGGTTCGCAACAATCCAGCGGTAGCGGCGAAGAGTTAAAGGAGTTGGCAAACAACAAAGTAAAAAAAAACGGCACTATCATTAAAGTAGATGAAATGTAG

>BdorGluRIID

ATGCAAGTACAAAACAACTGGATTTTTGGAATCTTATACACGTTTATTATTTATGTGAAGGGTTTCGCTAATATTGAAGAAAATGAGGGAATCTCAATTGGCATTATATCTGATGACAATATGGAGCCGTTACAGAAGACTTTTAACTATGCTATTACTGTAGCCAACACCGATTTAGGGATACCCTTGAAAGGGTATAACGAACAAATACAATTTGGCAATTCTATAGAAGGACATGCTAAACTTTGCAAATTTATGCAGACTGGCATAGGTGCTATTTTTGGGCCTTCTTCACGACAAACATCAGCGCATCTCTTAACAGTATGTGATGCTAAGGATGTACCTTATATATATCCACATATGAGTGAGAATGTGGAGGGATTCAATTTATACCCAAATCCAATAGATTTGGCACGTATTTTGCACGACATTATCAATTTATTCGAATGGACACATTTTACTTTCCTTTATGAATCTTCCGAATATTTAAGCATTTTAAATGGTTTAATGCCATTCTACGGTAGTGACGGACCAATCATCAATGTATTGCGATATGATCTTAAACTAAGTGGAAATTTCAAAGCAGTTCTGCGACGTGTACGCAAATCGGAAGAAGGACATATTGTAGTTGTAGGCTCTACACCATCTGTAGCTGAACTTTTAAAGCAGGCACAGCAAGTTGGAATAATGAATGATAAATATTCCTATATTATTGGTAATCTCGACTTACAAACTTTTGATCTGGAAGAATATAAATACAGTGAGGCGAATATAACCGGATTTCGAATGTTTTCTCCAACTCAGGCCATCGTTCAAGAATTAATATCACAATTGGAAATGGATTACAATGAAAACAATAATAACCAAATAGCCAATGGTTCCTGTCCAATAACTCTGGAAATGGCATTAACTTATGATGCCGTGCAAGTTTTTGCCGAATCTACAAAAAATTTAGTATATCGTCCACAAGCTTTGAACTGTTCCGAACAAAGCAACCAAGTGCAGGCTGATGGATCAACATTTAAAAACTACATGCGTTCGATTAACATGCAGGAAAAAACCATAACTGGGCCTATATACTTTGATGGAAACATACGAAAAGGTTATAGCCTAGATATTGTAGAGCTGCAAACTAGTGGACTTGTGAAGATTGGCACTTGGGATGAACGTAATAACTTAACAATTCAACGTCCACCTCAATCGGAACTATGGAGTGAGGTTGATGCCAATTCTTTGGTGAACAAAACATTTCGTGTTCTTATTTCAGTGCCAAACAAACCGTACGCCAGTTTAGTGGAGAGTCATAAGAAATTGGTCGGAAACAACCAGTATGAAGGATATAGCATAGACCTAATAAAGGAGTTAGCTGCAAAATTAGGTTTCAATTATACATTTATAGATGGTGGCAGTGATTATGGCAGCTTTAATAAAACAACAAATAAGACTACAGGAATGATGAAAGAGATAAATGAAGGCAGAGCAGATTTGGCAATTACTGATTTGACAATTACATCGGAACGCGAAGAAATAATAGATTTCTCTATTCCATTCATGAATTTAGGCATTGCTATTTTGTTTACTCAACCACAAAAATCGCCCGCTAACAATTTTTCATTCATGGATCCTTTCTCTAGACAAGTTTGGATTTATTTGGGCCTCGTCTATATTGGTGTATCGTTTTGTTTTTTCATATTGGGGCGATTATCGCCAACAGAATGGGACAATCCATATCCATGTATTGAAGAACCTGAAGAACTGGAAAATCAATTTACATTAAATAACTCCTTTTGGTTTACAACAGGGGCATTTTTACAGCAAGGTTCTGAGATAGCACCAAAGTCTCTTTCTACCCGCACTCTTGCATCTATTTGGTGGTTCTTCACCCTGATAATACTATCTTCATATACTGCCAATTTGGCAGCTTTTTTAACTATCGAAAAACCTGTTGGACTTATAAATAATGTCAATGAATTAGCTAGTGATACAAGAGTAAAATATGGTGCTAAAAAGACTGGTTCTACGCGAAGTTTCTTTTCAACCTCTGAGCATGAAACATATAAGAAGATGAATGATTTTATGGTCGAAAACCCGGATTTACTTTTCGAGACAAACCTTGAAGGAGTAAATCGCGTGAAAACCGATAACAATTATGCTTTCTTAATGGAATCCACATCAATTGAGTACCACATTGTGCGCGAATGTAATTTAAAGAAAGTTGGCGAACCACTCGATGAAAAGGGATATGGAATCGCAATGGTAAAAAATTGGCCATACCGCGACAAATTTAACAACGCGTTGTTAGAACTTCAGGAACAAGGCGTTTTGGCGCGACTCAAAAACAAATGGTGGAACGAAGTTGGTGCTGGTGTTTGTAAAAAAAAATCTGACAGCAGTGAAGTAAATCCCCTGGATTTAAAGAGCTTGGGTGGTGTATATCTAGTGCTTGGAGTCGGCAGCGGACTATCTCTAATATATAGTTTAATTATGTGGTGCATATATGTGGCAAGGAAATCCAACTATTATGAGGTACCCTTCGGTGACGCATTTTTGGAAGAACTACGTATTGCAATTGATGTTGCGAATAAGGAACGCATACTGAAGAGCGCACAATCCGTATACTCCCGAAGTCGAAACTCGCTTGTTTCAATCGATTCaATAGACACTGATTCTGAAATTGAAAACAGTTCAAAAATTGATAGAGAATCGGaGaAAACCATTTAG

>BdorGr5a

ATGCATAAATCATACTTAAATTATACACTTCTAAAGCAAATTGTGCGTTACCTAAAAGCAAAGCAAATCACGGAGCTAGAAAATGATGACGGTAGAAAGTACAGTGTTAAGCGTTTTTTAACTGGGCAACCACAGCAGCGCCGAAAGCGACTACAAATAATTAACGGCATCTTACCTGCCCCACGTAAGCTGTCAAAAAGGCAAACCACAATTGCTGTCGATGAGAAGCAGGAGTTAACCAAAGAGTTGTACGAACAAAGTACACATCCGAAAGTCCGCGGCATCAGGCGAGGTACACGCGCGGATTTCATACACAATGGCAGCTTTCATGAAGCCGTCGGCCCTTTGCTCGTGATTGCTCAGTGCTTCTGTCTGATGCCTGTGCGTGGCATACTCGCCGCCAGTCCGAAAGGGTTGTCTTTTCGCTGGAAGAGTTTTCGCACCTGGTACTGCATATTATATACGCTGGTGACCATTGCTGATACGGGCCTCACCATAAACATGGTCGTAAAAGGTGTGCTGGATGTGCGAAACATTGAACCATTAATATTTCATGCCAATATACTGCTGGCCTCAATTGGTTTCCTGCGACTGGCCGCAAAATGGCCGCAGCTGATGCGCAAATGGCAGCGAGTGGAGCGGCATATGCCGCCTTTTCAGTCGTGGCGTGAACGCGAGGCGCTGGCGGTGCGAGTACATAAGGTGACCTTTGTGCTGGTCACATTGTCGCTGACGGAGCATCTATTGAGCACCATTTCGGCCATTCACTTCGCCAACTATTGTCCTTCACGCGTAGATCCGATCGAGTCGTATTTCATGACTGTTGTTTCGCAAATTTTTTTCGTTTTCGACTACTCTACTTGGCTGGCTTGGTTCGGAAAGATATTGAATGTGCTCATGACTTTCGGTTGGAGTTACATGGATGTGTTTCTAATGATAATTGGCATAGGTCTCTCATCTTTGTTCGAACAGGTGCAGCGCAGTTTGGAGCGTGTCAAGGGGCAGGTCATGCCCGAATCTTTTTGGACGCGTACCCGCTTGCAATATCGTCTAATTTGTGATCTCATTGAACAAGTAGATTCCGCGGTATCTGCCATAACTGTGCTCTCTTTCGCTAATAATCTTTACTTTGTTTGTATTCAGTTGCTCAAGAGCATGAACACAATGCCATCTGTAGCACATTTTGTCTATTTCTATGCGTCACTATGTTTTCTGTTGGCACGCACCTTGGCGGTTTCTTTGTATTTGTCCGAGGTGAACGATCGCTCGCGTGAACCTTTAAAAATAATCAAAAAGGTGCCAAAGGAGGGCTTTCATCCTGAAGTTGACCGCTTGGCTCATAAGATTGGCATGGATACGGTAGCCTTGACCGGGTTGAAATTTTTTAACATTACGCGTGGTTTGGTGTTGACGGTTGCTGGTACTATTGTCACCTACGAATTAGTGCTTATACAGTTTCATGAAGATCAGAACTTATGGAATTGTAATTAA

>BdorGr21a

ATGGCCTACTGGGCGATAGCAACGCGCAAAGGTCAATCGCCGCCAATGAAGATCACACCCGTGCTGAATCCCAGTCAGCGTGAATTCCTCGAGGATGAGCTACTCTATCGCGAAAAGCTGGAAATACTTGCCGAAAACAACACGATCAGCACCGATCTGTTTGTgcgTAAGTTCGAGGATATCGACGATctcGTATTGCTAGACAAACACGATTCTTTCTATCACACCACCAAAAGTCTACTGGTGCTGTTCCAGATAATGGGCGTTATGCCAATACATCGCAATCCGCAGAAGCCGGGTATGCCACGCACCGGCTACTCATGGACATCGAAGCAGGTCTTCTGGGCTGTGTGCGTATTTTCGATGCAAACCACGGTTGTGGTGATGGTGTTGCGTGAGCGCGTGAATACGTTTTTGAACGATTCTGATCGGCGTTTCGATGAGGCGATCTACAATGTGATATTCATAAGTTTGTTATTCACGAATTTTCTCTTGCCGGTGGCGAGTTGGCGGCATGGTCCGCAAGTGGCGATTTTCAAAAATATGTGGACGAATTATCAATTGAAGTTTTTGAAAGTGACCGGCTCGCCGATTGTTTTCCCCAATCTGTATCCGCTTACCTGGAGTTTGTGCTTTTTCTCTTGGGGTGTGAGTATTGCGATCAATTTGTCACAATACTATCTGCAGCCGGATTTCAAATTGTGGTACACATTCGCCTATTATCCGATTATAGCGATGTTGAATGGATTCTGCAGCTTGTGGTACATCAACTGTACAGCTTTTGGCACTGCTAGTCGCGCTCTTTCCGCCTCACTGGAACTAACACTGATGAGTGATAAGCCAGCGAAGAAGCTCACCGAATATCGACATCTTTGGGTGGATTTAAGTCACATGATGCAGCAACTAGGTCGCGCCTACTCCAATATGTATGGCATGTACTGTCTGGTAGTTTTTTTCACAACCATCATTGCCACTTACGGTTCACTGAGTGAGATTATGGATCATGGCGCCACTTATAAGGAGGTCGGTCTCTTCGTCATCGTTTTCTATTGCATGAGCCTGCTCTATATCATTTGCAATGAGGCGCATTATGCCACACAAAGTGTCGGTTTGGACTTTCAAACAAAGCTCTTGAATGTCGATCTCACAGCGGTGGATAGCGCTACGCAAAAGGAGGTGGAAATGTTTCTCATGGCTATAACGAAGAATCCGCCGATTATGAATTTAGACGGCTATGCTAATATTAACAGAGAGTTAATCACTTCAAACATTTCCTTTATGGCAACGTACTTGGTTGTGTTGCTGCAATTCAAGATTACCGAACAGCGGAATTACTCGTTGAAGCAGAGCCGTGCAGAGCTGTTAGCATAG

>BdorGr28b

ATGGACGAAGATACGAACGGCATTGAGGCACAGGAAGTGCGCGCCACACGACCACAGCGCGTTAGCGGTTTACGTCGGTTCTTTCAGGCGCAACAGCTATACGAGAGCGTGCAGCCGCTTTTCGTGATCACTTTTTGGCACGGTCTCACGCCGTTCTTCATCAAAAGTGACGGCGCGGGCAATAAGAAATTGAAGGAATCCATTTTCGGTTATATCAACACGTTTCTGCACATCACCATCTATGTGGCATGTTATATGTTGACGCTGATAAATGATTTCGAAACGGTTGCGGGCTATTTCTTTAATAGCGGCGTCTCACGTTTCGGTGATACATTGCAAATTTTCAGCGGTTTGATCGGTGTGACGATCATATATATTACGGCTATGCTACCGAAGCAGCGTCTAGAGTACAGTTTACGCACAGTACAAGATATTGATCTCATGTTGCATAAAGTTGGTGTGAAAATAATCTACACCAAATTGTTGCATTACTCTTATTTTAGCATATTGTTGGTGGTTGCCGTGGACACAGTGTATTCGTGCGGTAATTTTATGCTCTTGAAATTGGCTAACTTAGAACCGTCAACGCCACTTTATGTCGTCTTTACGTTGCAACACACGGTTATCTCGATCGCTACGATGATGTATCACGGATTTGTGAAAATGCTGGAAATGCGACTAACTATGTTGAATGAGGTTTTAAAGAAACTCGCACATCAATGGGACAATAGCATTGTCAAACCAATGCCGAAGCAACGCTCACTACAATGTTTGGACTCATTCTCCATGTACACCATAGTCACAAATAATCCTTGCGAGATTATACAGGAATCTATGGAAATACATCATATGATTTGTGATGCCGCATCAACAGCAAATAAATATTTCACCTATCAACTATTGACCATCATTTCGATTGCATTTTTGCTCATCGTTTTTGATGCCTACTATGTACTCGAGATATTGTTGGGCAAATCGGCGCACGAGGGCAAATTCAAGACTGTGGAATTCGTGACTTTCTTTTCATGTCAAATGATTTTGTATGTCATTGCGATCGTATCCATTGTGGAGGGCAGTAATCGGGCTATTCAAAAGAGCGAAAAAAACCAGTGGCATTGTGCACTCACTACTGAATAA

>BdorGr32a

ATGTCGTCCAACAAAGTGAAGCCATCGCCAATACAGCGCCACCATAATGGTTTAAATCAATTCTTGCGGCAGCCACCAACGCATAACTCATTTTTAAGGTACATGCGAATAACATTATGTGTTTTGAAGGCAACAGGTCTGTTACCTATCTACGAAGAGGTTTCTAGTTATGAAGTTGGACCACCTACAAAGCCAAAAATTTATTATTCATTTTTCATTCGAGGAGTTGTACAAACCTTCACTTTATTCAACCTTTATAACCTTGTAACGCCTGGATCAACTGGACAACTGTTTTACTCCTACAGTGATACCGATAATGTGAATAAATGGATTGAGTATTTATTGTGTATGTTATCTCATTCAGCGACTGTTATAATTTGTGGTAGAAATTCAAAACTGTTTATAAAAATCTTAAATGAAATATTGAAAGTGGATGAAGGTGTCTTCGACCGATTCCGAGAAACTCTGAAAAATAAATGTGGATTCTCACTAAAGTACATTGTGGGCATATGTATTTGCCAATGGTATTTAATCGTATTACGCGTTTTAGCTGTTAAAGATACTCTTAACGTAAACTCATATATATTTCTATTTATCTACGCCGTGCAAAATGGCATGGCAACTATTTTCATTGTTTTTACGGCGGCTTTATTAAGAATCCTAAAGATGCGTTTCGCTCACATAAACACAACCCTTAAAGGTTATACGTATAGCGAGCAACATAAGCTACGACGTATACCAGGACGAGATAGGGATGTTATTACAATGGATTCTTTTCCTGAGGAATCTTTATTTATATACCGTCTGCACAATAAGTTGCTACGGATCTATCGTTCAATTAATGATTGCTGCAGCTTGATTTTGGTGGCATATATGGGTTATGCTTTCTACACTATTACCACTACTACCTATAACCTATTTGTTCAAATAACAACACAGCGCTTATCCTTTAATGTGTTACAAACGTGCTTTGTGTGGCTAGCAATGCATACTTGCGCCTTGGCATTACTGTCAAAAAATTGTGGACAAGCCACAGATGAGGTAAATATGTATATACATAAGTATGTGACTAATTGCTTTGAATTTCGTATTTATTATTTCTCTATAGGCTAA

>BdorGr63a

ATGTTTAACAGCTATAATCGTCGGAAAAAGCATGATACTGTCTTCTTAAATGTCAAGCCCACTTTTAACGGGCAGGGTAACGGGCTACGGAAGTATTCAACTGGTTTGCTGGATAAGGAGGACAATCCGTTTTACGATGTGAATAGTAGCAGGGGAAGTCGAGCATCGGTCGGAACTATAACCACATTGAATGAAAATTTCCGGGCCAATATTTTTTACAATAATATTGCGCCAATACAATGGTTTTTGTATATGCTCGGAGTTTTACCCATAACACGCCGGGAGCCTGGCAAGGCCAAATTTCGCATAAATTCCATTGCTTTCGGCTATTCGTTTGCCTTCTTTATACTTCTATCGGTTTTTGTTACTTATGTTGCTAAAAATCGTATAAGTATTGTGACGTCACTGAGTGGACCTTTTGAGGAAGCCGTAATTGCATATTTATTTCTCGTAAATATTTTACCGCTAATATTGATTCCAATATTATGGTGGGAGGCGCGTAAAATCGCTAAATTGTGGAATGATTGGGATGACTTTGAGATTTTGTATTACCAAATATCAGGTCACAGCATGCCCTTGAATTTGCGTCGTAAAACAACTATGATTGCGGTGGTTTTGCCGATTTTATCCATTTTATCGGTCGTCATAACGCATATAACGATGGCGGATTTTCAAATAATTCAAGTAATTCCATACTGCATCTTAGATAACTTGTCTGCCATGCTAGGAGCTTGGTGGTTCATAATATGCGAAAGTCTCAGCATGACGGCCAATATACTGGGCGAACGCTTTCAAAGGGCACTCCGTCACATAGGTCCCGCCGCCATGGTCGCCGATTATCGCGCCTTGTGGTTACGTTTGAGCAAATTGACACGCGATACCGGCAATGCGACCTGCTATACTTTCACTTTTATCAATTTGTATCTCTTTTTTATTATCACACTCTCCGTTTACGGACTCATGTCACAGCTCTCCGAAGGCTTCGGCATTAAGGATATCGGTTTGGCTATAACGGCTATTTGGAATGTTTTTTTAGACTTTTCTTACATTTGCGATAAAGCTCATTACGCCTCCTTCAATGTGCGCACGAATTTCCAGAAAAAGCTGTTGATGGTCGAACTGAATTGGATGAACTCGGACGCGCAGACGGAGATAAATATGTTCATACGCGCTACGGAAATGAATCCGTCGAATATCAATTGTGGTGGATTCTTCGATGTGAACAGAAATCTTTTCAAAGGCCTGCTCACCACCATGGTCACTTATCTAGTTGTCTTGCTGCAGTTCCAAATTAGCATACCCACCGACACCAGTCGGCACATGAACGTTTCGGTGGCCGAGTTAGCCACCGACATGATGCTGGAAAGTGCTGAGGACGAACTAACCACCACCAGTACAACATCGACAACAACAACCACAACGACTAAGATGCCACCGCCGGCACGTGGCAGGAAGGGATAA

>BdorGr68a

ATGGGGAGTACGGCTGCAGTCTTTACACTTTACTCGCGCGACCAGCACACCGCTTCTACAAAAATGTGGACTAAACGACGCGTCTCCTCCTACAAAAGTATGAGTAACATAAAAATTCGTAAAGCGCCAGAGCCCAGCCCGTCCTCATTCCTTGCAAGCATCAAGTGGAATGTGTTGATATTGAAGTTAGTTGGTCTGGTGCCATTCTATACATCGCCGAACCCTGATGAGATCGCCGCACCCAAAGGGCTACCAATACACATCACTCGAGCCATCTTTTGCGCTAGGACCGCCATGCACCTACTGCATGTCTACGCCTTGTCGTCGCCCTTCATTATGCAGAAACTTTTTCTGCGAAGCAAAACGAACGGCATTGCGAACACACTTAGCGTCTGCTTCTGCATCTTCGGCGATGTGGTCATTAGCTGGTCGTGCGCTCGCAACGGCACCAAAATCATCGCGATAATTAACGGATTTCTGAAAATCGATCGACGTATGAGACAACTGGCCCCGCCGCCAGCACAGCGCCAAAAAATTAGAAATGCGTTCAATATGTGTTTGTTGTTGCTATTTGGCTATCTCTGCGCAATAACAATACCGGTGGTGAAATGTTTCTACGGCACTCTGCCCGCCCACTTATTTCTGGGTATTTCATTCTACCAGGTGGAGAATGTTACCTCATGTGTGTTTGTTCTATTCATTGTGTCGCTGCTGCATCAGCTCACCTTGCGCATACAACAAACCAACAAGTTGATAGCTCAATACGGTGCGGAGAACGCAGCAGCGTCGGCATACAATGTGCAGAATTTCGCACGAAATTCCACAACATTTTACAGTTTACATAACGAGTTGTTGGACTTGCTGCAAATGATCAACAAATTCGCCGGTCTGGGTCTGGTGGTATTCCTCCTCTATGCATGCGCGGGTCTGCTGACCTTCACTTATGCCTGCACTTTGTACGATTTGCGCAATGGCGATGACTGGTTCAATATAATGTGGAACTTATCCTGGATACTGAACTTCGCGACCGTTTTGATATTGCTGGCGTTCCGCTGTGATTGCGTCACCAAAGAGGCTAATAACACAGGGCAGATACTTGCGCGCGTTTACGCCAAGGGCAAGGACTACCAAGATATCGTGGATAAGTTTCTGTCGAAGAGCATTAAGCAGGAAGTGCAGTTCACCGCTTATGGCTTTTTCTCCATTGACAATACAACACTATTCAAGATTTTCTCCGCTGTGACCACTTACCTGGTTATATTGATTCAATTCGAGCAGCTGGAAGACTCAAAGGCCGAAGAGTAG

>BdorSNMP1-1

ATGAAAGTGGAAAGGCATAAACTTTTGATCGCATCTGTGGCGGCGATGCTGTTCGGCGTGATTTTTGGTTGGGTCGGCTTTCCGAAAATACTCAAGATGATGATTAAAAAGCAAGTCTCACTTAAACCCGGCACTGAAATACGCGACCTCTGGACACAGACGCCATTTCCACTACACTTCTACATTTATGTATTCAACATTACCAATCCGGATGAGGTGTTGAACGGCGAAAAGCCCAATTTACAAGAAATCGGACCATTTGTTTTCGATGAATGGAAGGATAAGTACGATCTGGCCGATGATCCATTGGAAGATTCGATCTCGTTCAATATGCGCAACACATTTTATTTCAACGAAAAAGATTCAAAAGGACTAACCGGTGAAGAGCTCATAACAATTCCACATCCATTGCTTGTGCCGATCTCCGTGGTTGTGCAGCGCGATCGTGCCGCCATGTTGGATCTCGTCTCCAAAGCTATAAATATTGTGTTCGCCGGGCAGAAAGCGGTAATCACAGTTAAATTTATGGATGTATTCTTTCGGGGACTTTATGTGGACTGTTCGTCCACGGAATTTGCCGCTAAAGCACTCTGCACGGCTTTCTACACGGGTGAGGTGAAACAAGCGCGACAAGTGAATTCAACACATTTTCTATTCTCGTTTATGGCCAATAACAATCACACCGACGGCGGCCGCTTTACCGTCTGTCGTGGCGTCAAAAATGTAAGCAAGCTTGGCAAAGTCATACGTTTTGGTGATGAGCCGACATTGGATATATGGGCCGGCGAAGAGTGTAACGAATTTATCGGCACAGATTCGACGATTTTTGCACCATTCATGACCAAAGAGCAAGGCTTATGGGCCTTCACGCCCGATGTATGCCGCTCCCTCGGCGCCACCTACAAACGTAAGTCCTCATATCATGGCATGCCAGCAATGCGCTATCACATGGATTTGGGTGACATAAAGGCAGATCCGAGCTTGCATTGCTTCTGCGACGATCCGGAGGATGTTGACTCTTGTCCGCCCAAAGGCACTATGAATTTAGAACCTTGTGTTGGTGCTCCAATCATGGCATCGATGCCACACTTTTTTAATGCGGATCCTAAACTGATGGAGGAAGTGAACGGTTTAAGCCCGAATGAGAAGGATCATGCGGTGTTCATTGATTTCGAATTGACCTCTGGCACGCCTTTTCAAGCCGCCAAACGCTTGCAGTTCAACATGGACATGGAACCGGTTGAGAAAATTGAACCCACCAAGAATTTGCGTAAAATGATTTTTCCACTATTTTGGATTGAGGAAGGCGTGGCACTAAATAAGACCTTCACAAACATGCTAAAATATACACTATTTCTTGGTCTAAAATTCAATTCTGCGTTGCGTTGGTCGCTCATCACCATGGCTTTGGTCGGTCTCATGTCCACCTCATATTTGTATTACAAGAAGAGCGATAGCTTAGATATCACTGTGCCACCGAAAGCCATAAAGGAGCTCGCCAATAAAGTGGAGGATGTAAAGCCATTGCCACCCGAAGATAAAAAGCCCGTACCACCTATTGCACAGGCTGATCTCTCGAACCGTCGTGATGCGATGGATAGATTTTAG

>BdorSNMP1-2

ATGTTTAAGAAAATACTCATCGGTTCGGCTATTGCGCTCGTGTTGGGGATCTTTGTTGGTTTTATTGGTTTTCCAAAGTTATTGAATAAAATGATAAAAGGGCAATTAAATCTAAAACCTGGCAGCGAACCGCGACAAATGTGGGAGAAATTTCCCATCGCACTGAATTTTTCAATTTACGTCTTCAACGTAACAAATCCCGATGAAGTGCAAAATGGCGGAAAACCACGTGTTCAAGAGGTCGGGCCATTTGTATTTGAGGAATGGAAGGACAAATACGATTTGGAGGACTTCGAGGATGAGGACGCGGTGGCCTACAACATGCGTAACACATTCATTTTCAGACCTGATTTGGGCCTAAGCGGCGAGGATTTAATCGTGATGCCACATCCACTTGTACAAATCATGGCTATAGCCGTCAAGCGTGACAAGGAAGCCTTGATCAACATGATATCCGAGGGTTTGCAAGCGCTATTTAAGCCCACCACGCCATTTGTGCACGCACCTTTCATGGACATTTTCTTTCGCGGCATCGATGTAGACTGCTCCATCGATCATTTTGCAGCTAAGGCGATTTGTTTGAATTTTCACACCGGTGCCATCAAGGGAGCGGAAAAAGTAAATTCAACACATTTCAAATTTTCGCTCTTTGGCGGGGCCAACCACACCGACGCCGGTCGCTATAAGGTCGCGCGTGGCGTCAAGGTTAGCCGTGATATTGGACGCGTGCTTGAGTTTGACGACTCTGACGAGCTGAGTGTCTGGGACGGTGATGAGTGCAATCAATTTCGCGGCACCGACACAACCATCTTTGCACCGCTAATGAAGCCCGAAGAGGGATTGTGGTCGTTTGCTGCCGATTTGTGTCGTTCTTTGGGTGCCGAGTTCGAGAAGAAGACCACTTATGCAGGTATACCGGCATACTATTACACAATCGATTTGGGCGATCCAAAGAACGATCCAGATAAGCATTGTTTCTGCAAGGATTATCCCGACGACTGTCCACCCAAAGGTACCATGGATCTCACCTTGTGCAATGAGGCACCCATGATTGTCTCGTTGCCACACTTTTTCAAGGCCGATCCGCAATTGGTTGCGGACGTAGATGGTTTGGATCCAGTGGAAGAAAAGCATGGCGTTTTTATTGTTTTCGAAAGGATATCTGGTACACCCCTCTCTGCCGCTAAGCGCCTGCAATTCAGCCTCTCCGTGATGCCCGTGCCGGAAGTAGAGGTTATGAAGAACTTACGCACACTCACCATGCCGCTCTTTTGGGTCGAAGAAGCGGCCAGTTTGGATAAGACATGGACCGATATGTTGAAGAAAAAGGTTTTCTTAGTCATCAAAATAAATAATGTCTTTAAATGGATGTCGACAATTTTCGGGGCATTGGGTTTAGCAATCAGCCTCTATATGCTATTTGGTCGAAATCAAATCACCACAACCAATGTCACACCCACAACCGAAGCCAGCACGATCGATAAACATTGA

>BdorSNMP2

ATGTTTCATTGGTCATTGTTCGTCAGCTTGTTGGGTTTGCTGACAGCTGCGCTGGGCGCCTATTGCGGCTGGTTCCTGTTTCCCAACATGGTCGATAAGAAAGTGGAAGAGAGCGTAATTATTGCTGATGGCTCTGAACAATACAAGCGATTCGTTCAACTCCCACAGCCGTTGACCTTCAAAGTGTATATTTTTAACGTGACCAATGCACACAAGATACAACAAGGCGCCATACCAATTGTGGAGGAGATCGGACCCTATGTATACAGACAATATCGGCGCAAGAAGGTGAAACACTTCTCACGTGATGGTTCTAAGATTAGCTATGTGCAGGATCAGCACTTTGAATTTGACGAAGAAGCCTCGGCACCTTACACACAATCCGATCACATTGTTGTGCTTAATATGCATATGAATTCGGTGCTGACGATCGCCGAGAATGATCCGGGCTTGTCGTTGTTGCTGGTGCACTTGAATGCCAATCTGAAGGCCGTCTTTAACGATCCCAAGTCGATGTTTCTCGACACCACCGTGCGTGAGTTCCTCTTCGATGGCGTGCGTTTCTGCATAAACACGAATGGTATTGCTAAAGCTATTTGTAATCAAATCAAAGAGGGCGGTTCCAAGACAATACGTGAACTAAGCGACGGCAGCTTGGCATTCTCGTTCTTCAATCACAAAAACGGCACTGGCAATGAAGTGTACGAAGTGCATACGGGCAAAGGCGACGCACAGCGTGTGCTCGAAATTCAGAAACTCGACGATTCACACAATCTTCAGGTGTGGCTGAATGGTTCCGAGGGCGAAACTTCTATGTGCAATCAAATCAATGGCACTGATGCTTCATCGTATCCGCCCTTCCGCAAACGTGGTGATTCTATGTACATTTTTAGTGCGGATATTTGTCGTTCGGTGCAGTTATTCTATCAAAGTGATATACAGTATCAGGGCATACCCGGTTTTCGCTATTCGATCGGTGAGAATTTCATCAACGATATTGGTCCGGAGCACGACAATGAGTGCTTCTGTGTTGACAAGCTGGCGAATGTGATAAAACGGAAGAATGGTTGCCTATATGCGGGCGCACTGGATCTGACAACCTGCTTGGATGCACCGGTTATATTAACATTGCCGCATATGTTGGGCGCCTCCAATGAGTACACGAAAATGATACGCGGACTGCGGCCGGACGCGAAGAAGCATCAAACATTCGTGGATGTACAGCACCTCACCGGTACACCGTTACAAGGCGGCAAACGCGTGCAGTTCAACATGTTCTTGAAGAGCATCAACCGCATCTCAATAACGGAAAATTTAACAACGGTACTTATGCCCGCCATTTGGGTTGAGGAGGGTATTCAACTCAACAGTGAAATGGTCGCCTTTTTCAAAAAGAAACTTATAAACTCGCTCAAAACGCTCAATATCATACACTGGGCGTCGATTTGCGGTGGCATTGGCGTGGCCGCTATATGTCTGATATATTATGTAATACAACGTCGAAAGCCGGAAGCGGAAGTGGCACCGCTAAAATAA
